# Catalytic Diastereo- and Enantioselective Synthesis of Tertiary Trifluoromethyl Carbinols through a Vinylogous Aldol Reaction of Alkylidenepyrazolones with Trifluoromethyl Ketones

**DOI:** 10.1021/acs.joc.1c02817

**Published:** 2022-03-16

**Authors:** Laura Carceller-Ferrer, Aleix González del Campo, Carlos Vila, Gonzalo Blay, M. Carmen Muñoz, José R. Pedro

**Affiliations:** † Departament de Química Orgànica, Facultat de Química, Universitat de València, Dr. Moliner 50, 46100 Burjassot, València, Spain; ‡ Departament de Física Aplicada, Universitat Politècnica de València, Camino de Vera s/n, 46022 València, Spain

## Abstract

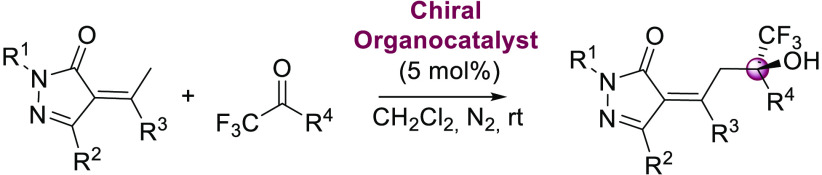

A diastereo- and enantioselective organocatalytic aldol reaction between alkylidenepyrazolones and trifluoromethyl ketones leading to chiral tertiary alcohols bearing a trifluoromethyl group is presented. The methodology is based on the use of a bifunctional organocatalyst in order to activate the γ-hydrogen atoms of the alkylidenepyrazolone nucleophile and the carbonyl group of the trifluoromethylarylketone providing highly functionalized trifluoromethyl alcohols with moderate yields, excellent diastereoselectivity, and moderate to good enantioselectivity. Experiments monitoring the conversion by ^1^H NMR and the enantiomeric excess by HPLC with the reaction time showed that full conversion of the starting materials is not achieved and that the enantiomeric excess decreases upon extended times, probably due to the reversibility of the reaction.

## Introduction

The definition of vinylogy is the transmission of the electronic effects through a conjugate system. Therefore, vinylogy allows the extension of the nucleophilic or electrophilic character of a functional group along to the conjugated π-system of the C=C bond.^1^ This phenomenon has ascertained to be very valuable to expand the range of reactions of different functional groups that can be coupled efficiently through the π-system of a carbon–carbon double bond. In this context, catalytic asymmetric vinylogous reactions are potent and sustainable methodologies for the synthesis of molecules with stereogenic centers at the γ-position or even more remote positions of the functional groups. Of all the enantioselective vinylogous reactions described in the literature, the organocatalytic vinylogous aldol reaction^2^ represents a cornerstone in synthetic organic chemistry and have been used for the synthesis of chiral γ-hydroxyl carbonyl compounds in an efficient and sustainable way.

**Figure 1:**
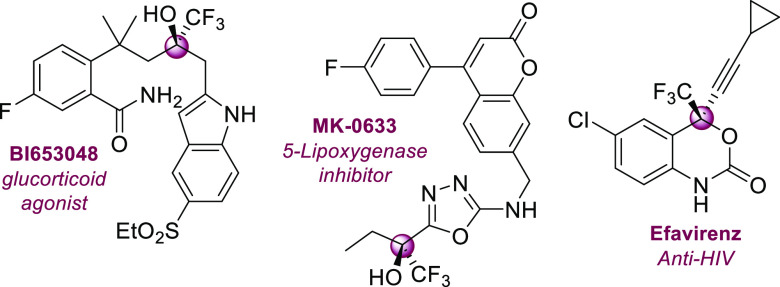
Representative bioactive compounds bearing trifluoromethyl carbinol motifs.

**Scheme 1:**
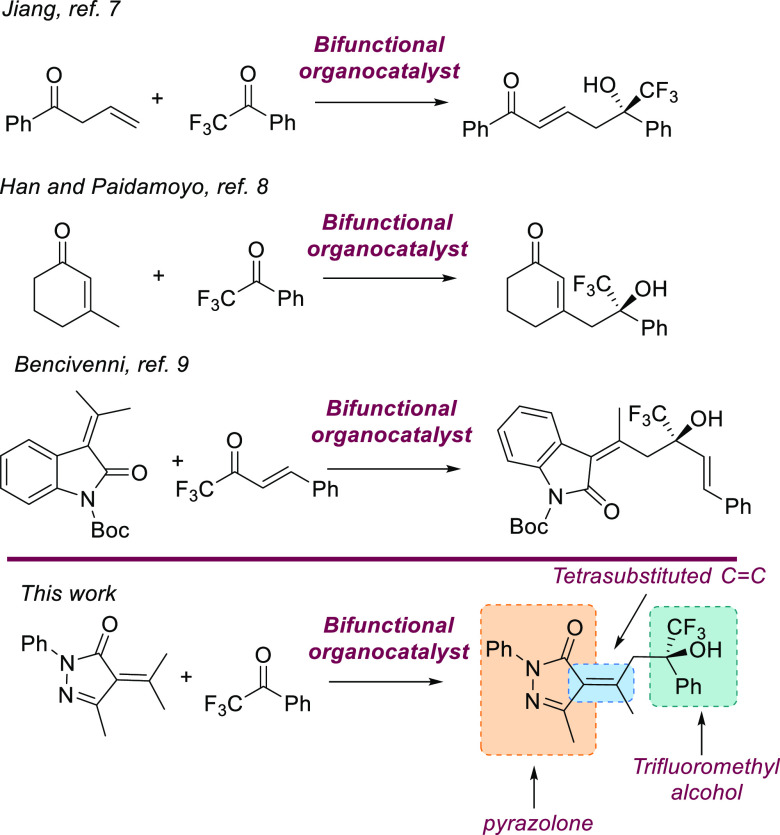
Examples of Asymmetric Synthesis of Tertiary Trifluoromethyl Carbinols through an Organocatalytic Asymmetric Vinylogous Aldol Reaction

Within the different types of chiral alcohols, chiral tertiary trifluoromethyl carbinols^3^ constitute a key structural motif present in a wide range of molecules with important biological activities (Figure 1).^4^ This fact is due to the significant properties of organofluorine compounds that, in general, improves the bioactivities of agrochemical and pharmaceutical compounds. Therefore, several examples of asymmetric synthesis of tertiary trifluoromethyl carbinols have been described. From all the methodologies described, the enantioselective aldol reaction with trifluoromethylketones is one of the most straightforward approaches for the synthesis of this kind of tertiary alcohols.^5^ Nevertheless, the vinylogous aldol reaction with trifluoromethyl ketones have received less attention (Scheme 1), despite the possibilities for the synthesis of highly functionalized chiral trifluoromethyl carbinols. Jiang and co-workers, in 2016,^6^ described the enantioselective vinylogous addition of acyclic allyl ketones to trifluoromethyl ketones using a bifunctional thiourea organocatalyst. Later, Han and Paidamoyo reported the vinylogous aldol reaction of 3-methylcyclohex-2-en-1-one to a wide range of trifluoromethylarylketones with very good results using a diamine-sulfonamide organocatalyst.^7^ Also Bencivenni’s group^8^ presented the vinylogous aldol addition of alkylidene oxindoles to trifluoromethyl-α,β-unsaturated ketones obtaining chiral trifluoromethylated allylic alcohols in moderate yields (48–88% yield) and with excellent enantioselectivities (up to 96% ee). Moreover, several examples of an enantioselective vinylogous aldol-lactonization cascade reaction have been reported in the literature for the preparation of chiral unsaturated δ-lactones bearing a trifluoromethyl group.^9^ For example, Chi described the γ-functionalization of enals^9a^ and α-branched heteroaryl aldehydes^9b^ for the synthesis of lactones using N-heterocyclic carbene (NHC) organocatalysis. While Bencivenni described the synthesis of trifluoromethylated α,β-unsaturated δ-lactones with excellent stereochemical outcomes using alkylidene oxindole and trifluoromethyl ketones as starting materials and a bifunctional thiourea as the catalyst.^9c^ Despite these examples, it is possible to envision other γ-enolizable α,β-unsaturated carbonyl compounds that can be used in vinylogous aldol reactions using trifluoromethyl ketones as electrophiles. As a part of our continuing work in the asymmetric functionalization of pyrazolones,^10,11^ we hypothesized that alkylidenepyrazolones^12−14^ could be a suitable nucleophile to perform a vinylogous aldol reaction using trifluoromethylarylketones. The resulting reaction would lead to a novel synthesis of chiral trifluoromethyl alcohols bearing a tetrasubstituted C–C double bond and a pyrazolone moiety, which represent an important class of nitrogen heteroaromatic framework present in several biological active compounds.^15^ Several asymmetric vinylogous reactions of alkylidenepyrazolones have been described in the literature for the synthesis of chiral pyrazolones. However, these examples are limited to their use in the nucleophilic addition to α,β-unsaturated compounds,^12a−12j^ Morita–Baylis–Hillman carbonates,^13^ and isatin-derived ketimines^14^ as electrophiles. To the best of our knowledge, the corresponding asymmetric nucleophilic 1,2-addition to carbonyl compounds is unprecedented.

## Results and Discussion

**Figure 2:**
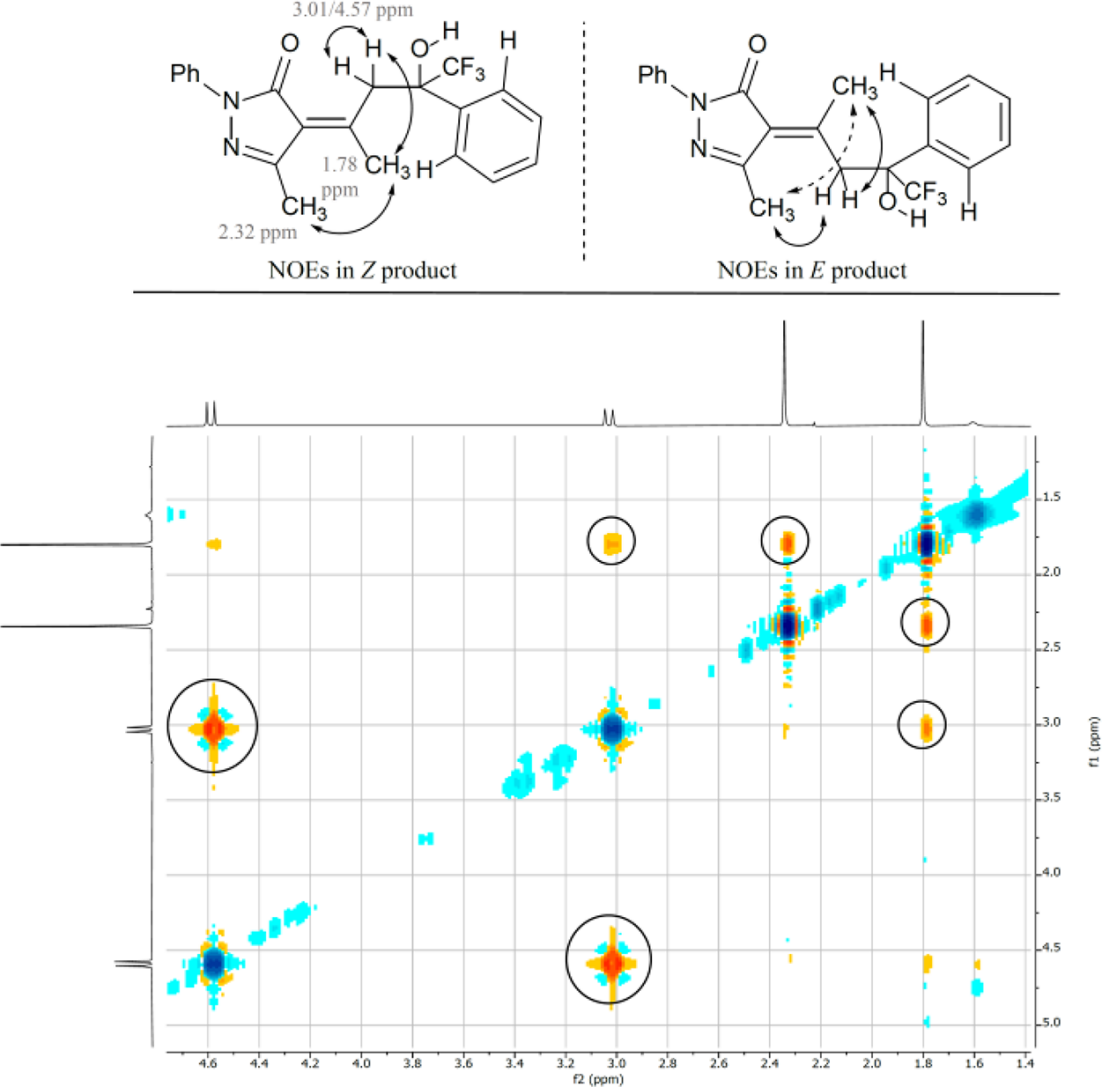
NOESY experiment with compound **3aa** showing the *Z* configuration of the double bond.

We started our studies with the vinylogous aldol reaction of α-isopropylidenepyrazolone (**1a**) with trifluoroacetophenone (**2a**) testing different bifunctional organocatalysts^16^ (Table 1) using CH_2_Cl_2_ as a solvent at room temperature. First we tested quinine (**I**) and cinchonidine (**II**) as catalysts observing low reaction rates. After several days we could isolate product **3aa** as a unique diastereoisomer in 49% yield and a promising 38% ee using quinine as catalysts (entry 1, Table 1), while cinchonidine afforded the chiral alcohol **3aa** in 36% yield and 24% ee (entry 2, Table 1). Cupreine **III** gave inferior yield and enantiomeric excess than **I** (entry 3). When 5 mol % of Takemoto’s thiourea **IV** (entry 4) was used as a catalyst, we observed better conversion and enantioselectivity toward the aldol product **3aa**, which was obtained with 52% yield and 65% ee. Cinchona-derived thioureas **V** and **VI** exhibited higher stereocontrol (77% and 76% ee, respectively); however, the yield of product **3aa** was still moderate (entries 5 and 6). Thiourea **VII**, prepared from dihydroquinine, exhibited lower enantiomeric excess, and product **3aa** was obtained with 50% yield and 52% ee after 3 days (entry 7). Next squaramides **VIII** and **IX** were tested. With organocatalyst **IX** (entry 9), the alcohol **3aa** was obtained with good enantiomeric excess (74% ee) and moderate yield (51%).^17^ In all cases, we only observed one diastereoisomer.^18^ The configuration of the double bond in chiral aldol adduct **3aa** was determined as *Z* using a NOESY experiment (Figure 2). We observed positive NOEs between the two methyls groups attached to the alkene and the heterocycle (2.32 and 1.78 ppm, respectively) indicating that they are close to each other. In order to improve the yield of the reaction, we increased the amount of organocatalyst to 10 mol % **VI** (entry 10) and **IX** (entry 11), noticing lower enantiomeric excesses.

**Table 1:**
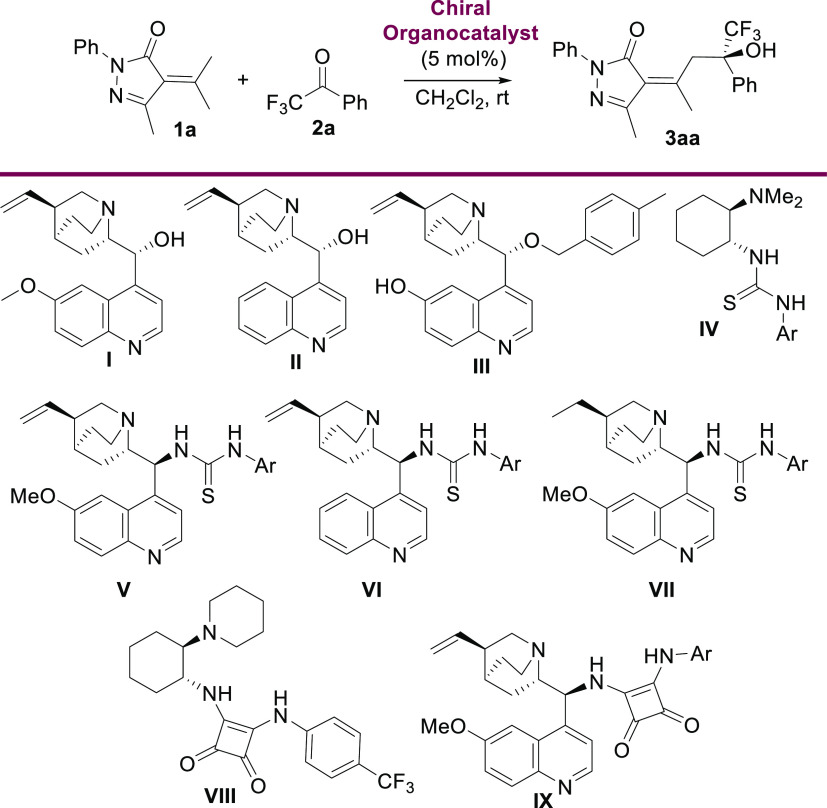
Optimization of the Catalysts^a^

entry	catalyst	*t* (days)	yield of **3aa** (%)^b^	ee of **3aa**^c^
1^d^	**I**	4	49	38
2^d^	**II**	3	36	24
3	**III**	4	19	36
4	**IV**	2	52	65
5	**V**	3	37	77
6	**VI**	4	52	76
7	**VII**	3	50	52
8	**VIII**	5	53	58
9	**IX**	3	51	74
10^d^	**V**	3	51	68
11^d^	**IX**	4	42	49

a Reaction conditions: 0.1 mmol of **1a**, 0.1 mmol of **2a**, 5 mol % of catalyst in 1 mL of CH_2_Cl_2_ at rt.

b Isolated yield of **3aa**.

c Determined by chiral HPLC.

d The reaction was performed using 10 mol % of catalyst.

In view of these results, we decided to choose catalyst **V**, the catalyst with the best enantiomeric excess, to continue further optimization by testing different solvents and additives (Table 2). Consequently, a survey of solvents (entries 1–6, Table 2) was tested for the vinylogous aldol reaction between **1a** and **2a** using 5 mol % of catalyst **V**. First, different chlorinated solvents such CHCl_3_, ClCH_2_CH_2_Cl, and CCl_4_ were evaluated obtaining product **3aa** with lower yields (entry 2) or lower enantioselectivity (entries 3 and 4). The use of other solvents such as diethyl ether or toluene did not improve the results obtained with CH_2_Cl_2_. Next, we evaluated the use of additives in order to increase the yield and the enantioselectivity of the reaction. When molecular sieves 5 Å or CF_3_CH_2_OH^19^ were added to the reaction mixture, the alcohol **3aa** was obtained with lower enantiomeric excess (69% ee, entries 7 and 8). While the use of 1 equiv of K_2_CO_3_ afforded product **3aa** as a racemic mixture, probably caused by a background reaction (entry 9). Finally, when 25 mol % of PhCO_2_H was added to the vinylogous reaction, we could not observe the formation of the alcohol **3aa**, probably due to a deactivation of the bifunctional organocatalyst **V** by protonation of the tertiary amine. The variation in the number of the equivalents of nucleophile (entry 11) or electrophile (entry 12) did not improve the enantioselectivity of the reaction. In view of these results, we decided to reevaluate catalysts **VI** and **IX** but extending the reaction time to 5 days. We could increase slightly the yield of the reaction (entries 15 and 17), maintaining the enantiomeric excesses. Taking into account these results, we decided to use these catalysts to study the scope of the reaction.

**Table 2:**
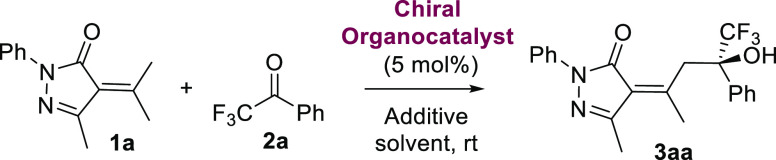
Optimization of the Reaction Conditions^a^

entry	catalyst	solvent	additive	*t* (days)	yield of **3aa** (%)^b^	ee of **3aa**^c^
1	**V**	CH_2_Cl_2_		3	37	77
2	**V**	CHCl_3_		4	33	77
3	**V**	Cl CH_2_CH_2_Cl		3	42	61
4	**V**	CCl_4_		3	49	49
5	**V**	Et_2_O		2	43	64
6	**V**	toluene		2	58	54
7	**V**	CH_2_Cl_2_	MS (5 Å)^d^	4	43	69
8	**V**	CH_2_Cl_2_	CF_3_CH_2_OH^e^	4	47	69
9	**V**	CH_2_Cl_2_	K_2_CO_3_^e^	3	39	0
10	**V**	CH_2_Cl_2_	PhCO_2_H^f^	5		
11^g^	**V**	CH_2_Cl_2_		3	44	71
12^h^	**V**	CH_2_Cl_2_		3	46	67
13	**V**	CH_2_Cl_2_		5	46	74
14	**VI**	CH_2_Cl_2_		4	52	76
15	**VI**	CH_2_Cl_2_		5	61	74
16	**IX**	CH_2_Cl_2_		3	51	74
17	**IX**	CH_2_Cl_2_		5	46	77

a Reaction conditions: 0.1 mmol of **1a**, 0.1 mmol of **2a**, 5 mol % of catalyst in 1 mL of solvent at rt.

b Isolated yield of **3aa**.

c Determined by chiral HPLC.

d 50 mg of MS 5 Å.

e 0.1 mmol of additive was used.

f 0.025 mmol of PhCO_2_H was used.

g 0.12 mmol of **1a**.

h 0.12 mmol of **2a**.

**Scheme 2:**
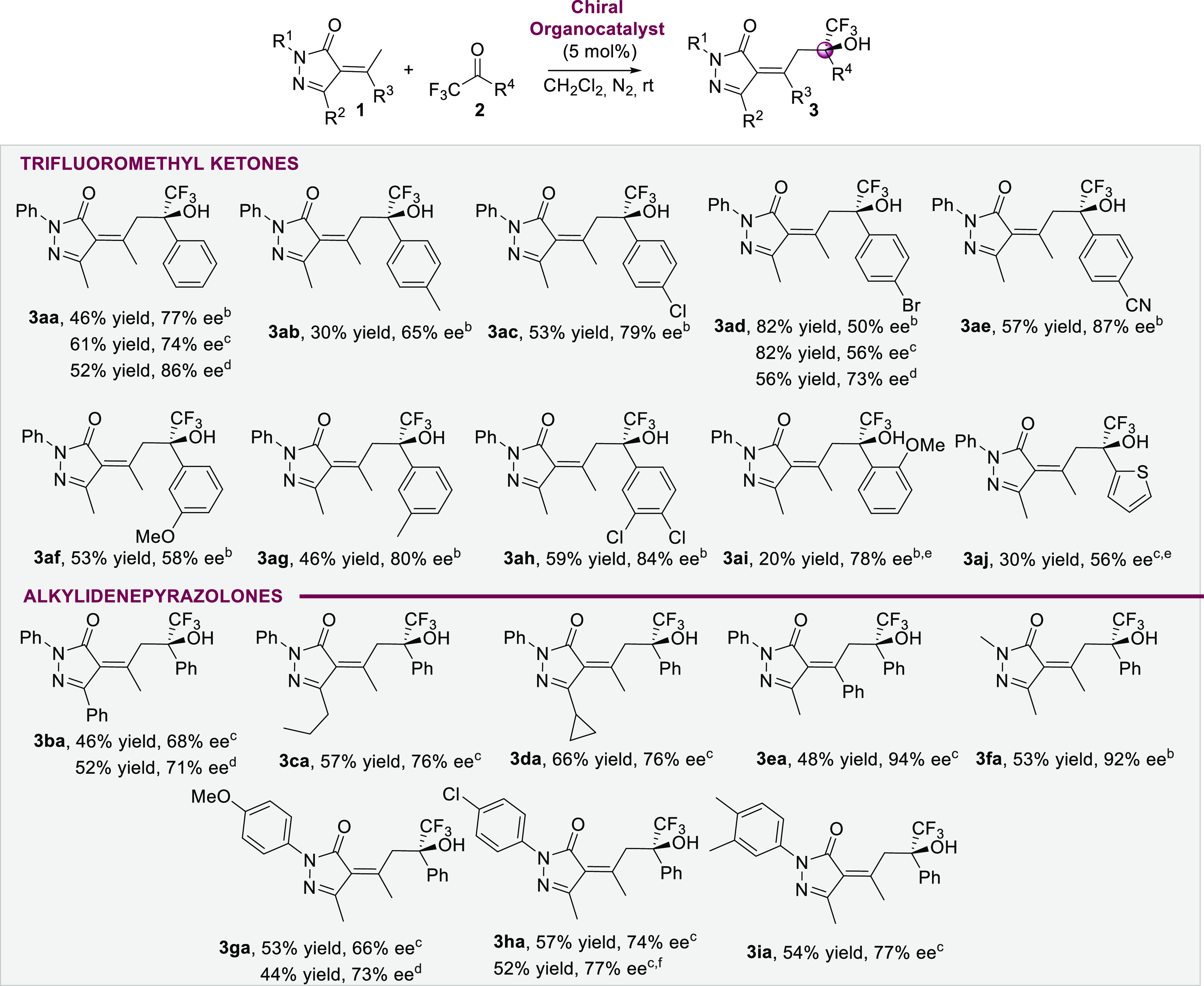
Scope of the Vinylogous Aldol Reaction of Pyrazolones **1** with Trifluoromethylarylketones **2** Reaction conditions: **1** (0.1 mmol), **2** (0.1 mmol), and bifunctional organocatalyst (5 mol %) in 1 mL of CH_2_Cl_2_ at 20 °C. Isolated yields after column chromatography. Enantiomeric excesses were determined by HPLC using a chiral stationary phase. Squaramide **IX** was used as the catalyst. Thiourea **VI** was used as the catalyst. Thiourea **VI** was used as the catalyst in 0.5 mL of CH_2_Cl_2_ at 20 °C. 10 mol % of organocatalyst was used. 1 mmol scale reaction.

First a range of trifluoromethylaryl ketones **2** were evaluated as electrophiles in the asymmetric vinylogous aldol reaction (Scheme 2).^20^ We observed a decrease in the yield and enantiomeric excess when the 4′-methyl-2,2,2-trifluoroacetophenone was used as the electrophile. While, the presence of electron-withdrawing (Cl or CN) in the para position were well tolerated obtaining better yields and enantioselectivities, strong electron-donating group (MeO) at the meta position had a detrimental effect in the reaction obtaining lower enantiomeric excess in product **3af** (58% ee). However, the presence of a methyl group at the meta position has a good influence on the course of the reaction affording product **3ag** with 80% ee. Remarkably, when 3′,4′-dichloro-2,2,2-trifluoroacetophenone was used as an electrophile, the corresponding chiral alcohol **3ah** was obtained with 59% yield and 84% ee. Low yield (20%) of the trifluoromethyl alcohol **3ai** was obtained probably due to the presence of a MeO at the ortho position to the carbonyl group. Moreover, we observed a decrease in the conversion and enantioselectivity when a trifluorometlyl ketone bearing a heteroaromatic substituent was tested.

We next turned our attention to further explore the scope with respect to the alkylidenepyrazolones **1**. Other groups such as phenyl, *n*-propyl, or cyclopropyl at the 5 position of the pyrazolones (**3ba**–**3da**) were well tolerated, obtaining moderate to good yields (46–66%) and good enantioselectivities (68–76% ee). Notably, the best enantioselectivities were obtained when the 2-phenyl-5-methyl alkylidenepyrazolone derived from acetophenone (**1e**) or 2,5-dimethyl alkylidenepyrazolone derived from acetone (**1f**) were used as nucleophiles in the vinylogous aldol reaction. The corresponding products **3ea** and **3fa** were obtained in both cases with an excellent enantioselectivity (94% ee and 92% ee, respectively), although with moderate yields (48 and 53% yield, respectively). Lastly, the reaction was tested using pyrazolones with diverse substituents (MeO, Cl, or Me) on the N-aryl group, obtaining the corresponding tertiary alcohols **3ga**–**3ia** with moderate yields (53–57%) and good enantiomeric excesses (66–77% ee). The reaction could be carried out at the 1 mmol scale obtaining product **3ha** with similar yield (52%) and maintaining the enantioselectivity of the reaction (77% ee).

**Scheme 3:**
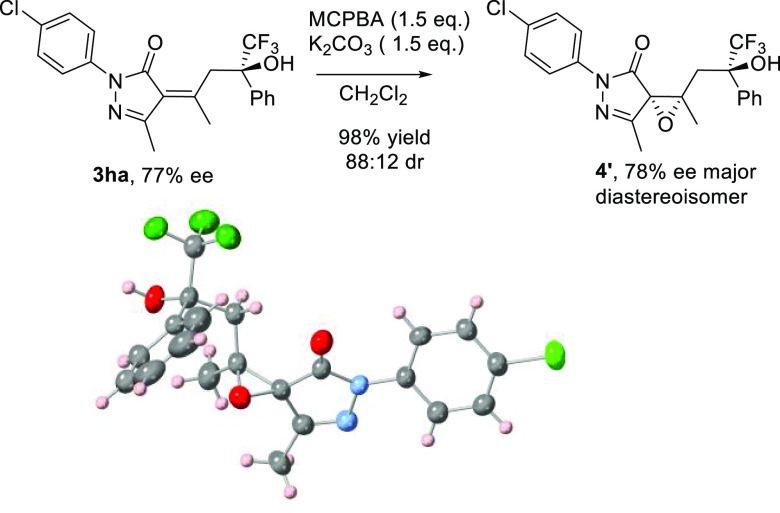
Epoxidation of Compound **3ah** and X-ray Structure of the Major Diastereoisomer

In order to derivatize the chiral trifluoromethyl alcohol **3ha**, we performed the epoxidation with MCPBA affording the spirooxirane **4** (Scheme 3) with three quaternary stereocenters, in 98% yield, good diastereoselectivity (88:12 dr), and maintaining the optical purity. We could obtain crystals of the major diastereoisomer **4′**, which allowed us to determine the absolute configuration of the epoxide and the chiral carbon bearing the trifluoromethyl alcohol.^21^ The absolute configuration of the three stereogenic centers in compound **4′** were determined to be (2*S*,3*S*) in the epoxide, while the configuration of the alcohol was determined as *R* on the basis of X-ray crystallographic analysis. The configuration of the remaining vinylogous aldol products **3** were assigned on the assumption of a uniform mechanistic pathway.

**Scheme 4:**
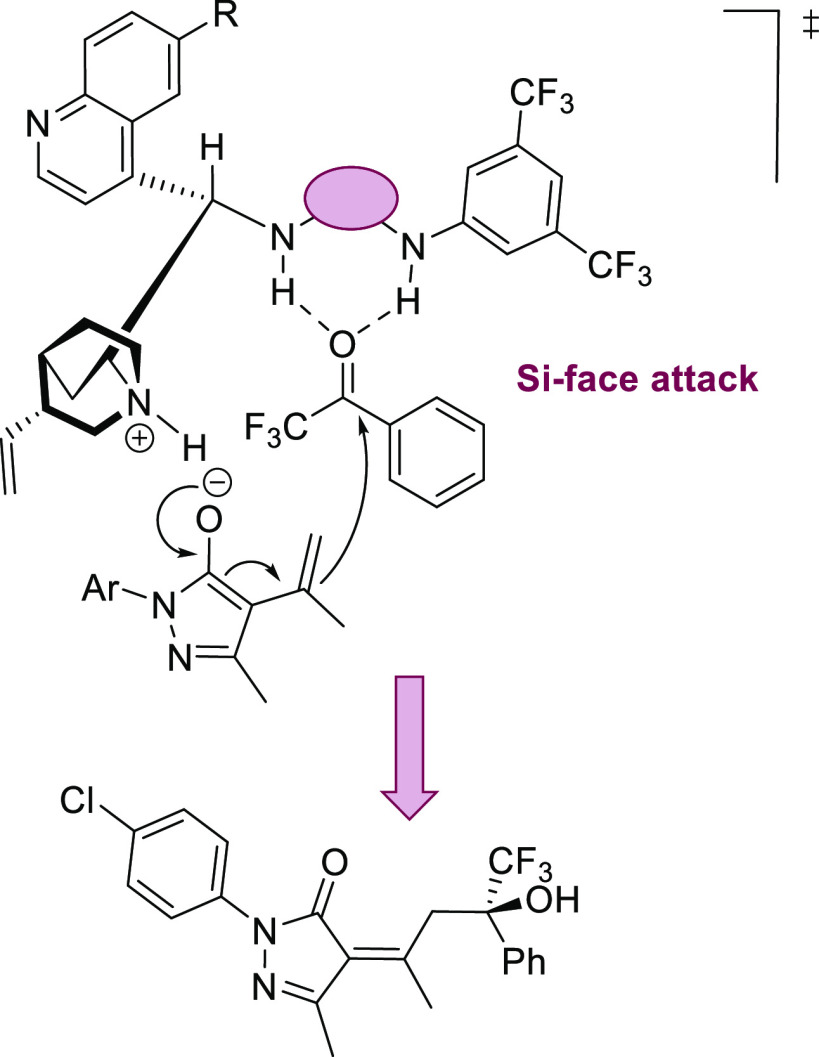
Plausible Mechanism for the Asymmetric Vinylogous Aldol Reaction

A reasonable transition-state model is represented in Scheme 4, where the bifunctional organocatalyst is responsible for the activation and preorientation of the reagents. While the methyl group of alkylidenepyrazolone is first deprotonated by the quinuclidine moiety of the organocatalyst to form the corresponding dienolate, the trifluoromethyl ketone is activated upon formation of hydrogen bonds between the carbonyl group and the thiourea or squaramide moiety of the catalyst. The nucleophile will be directed to the Si-face of the ketone, accordingly accounting for the observed stereoselectivity.

**Figure 3:**
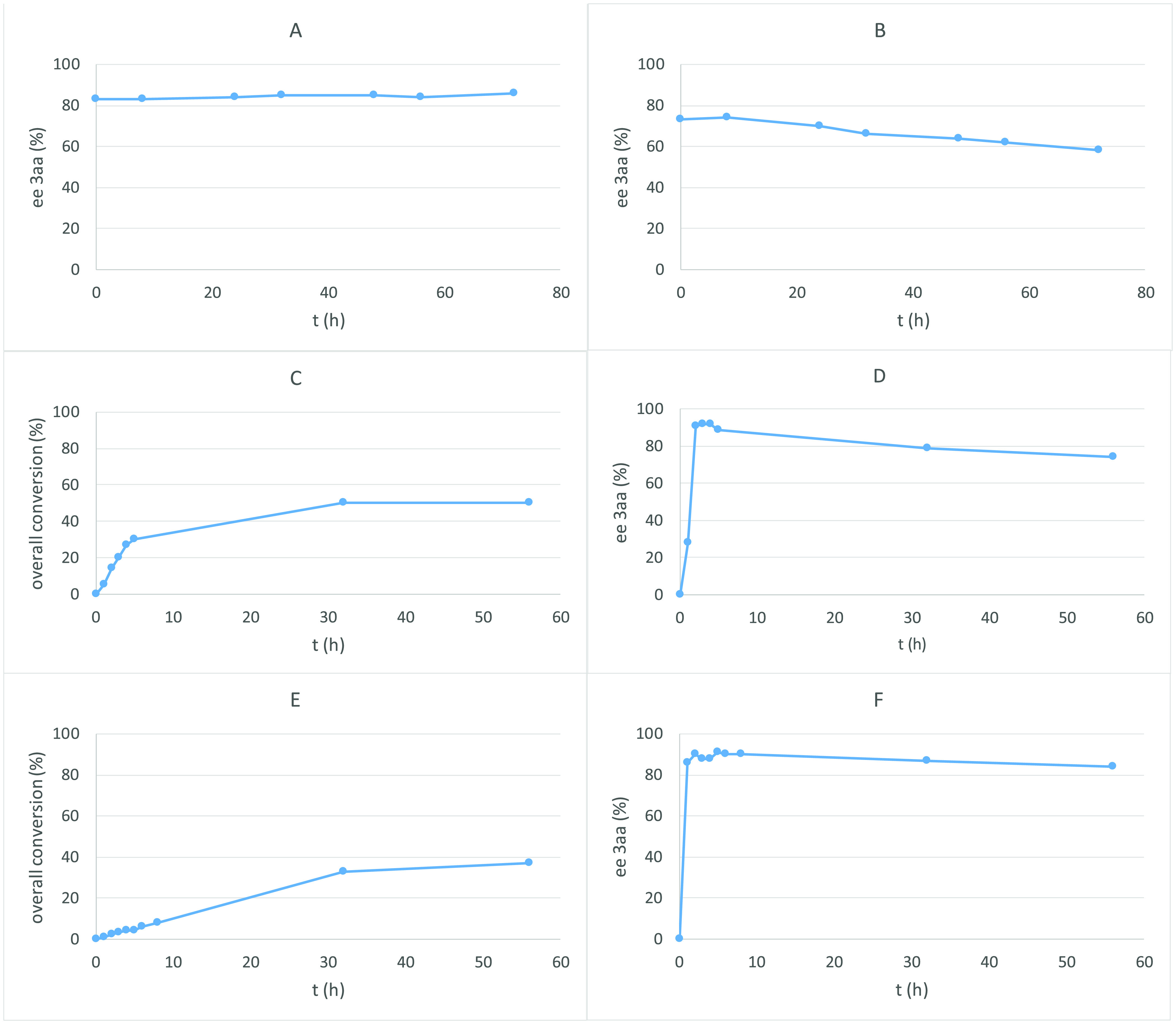
Studies about the stability of the aldol adduct **3aa** and kinetic investigations on the vinylogous aldol reaction: (A) compound **3aa** (83% ee) stirred in CH_2_Cl_2_; (B) compound **3aa** (73% ee) and catalyst **VI** (5 mol %) stirred in CH_2_Cl_2_; (C) conversion of **1a** to obtain **3aa** using **IX** (5 mol %) in CDCl_3_; (D) evolution of the enantiomeric excess of compound **3aaIX** (5 mol %) in CDCl_3_; (E) conversion of **1a** to obtain **3aa** using **VI** (5 mol %) in CDCl_3_; and (F) evolution of the enantiomeric excess of compound **3aa** using **VI** (5 mol %) in CDCl_3_.

To understand the reasons for the moderate yields and enantioselectivities observed in some cases or the alteration of the enantiomeric excesses by slight differences in the reaction conditions, we performed different experiments (Figure 3) according to a related previous report.^22^ We dissolved a sample of **3aa** (83% ee) in CH_2_Cl_2_, and we checked the enantiomeric excess along different times (Figure 3A), observing that the enantiomeric excess was maintained over time and therefore compound **3aa** is stable. However, when we dissolved a sample of **3aa** (73% ee) with 5 mol % of catalyst **VI** in CH_2_Cl_2_ (Figure 3B), a decrease in the enantiomeric excess was observed. This fact probably is caused by a retro-aldol vinylogous reaction, because we observed the presence of compound **1a** in the HPLC traces as well as in the TLC. This experiment prompted us to study the conversion and the enantioselectivity of the reaction between **1a** and **2a** using catalyst **IX** (Figure 3C,D) and **VI** (Figure 3E,F). For this purpose, the conversion of **1a** was monitored by ^1^H NMR and the enantiomeric excess of compound **3aa** by chiral HPLC at different reaction times. As indicated in Figure 3C, when squaramide **IX** was used as the catalyst, the reaction equilibrium was reached after 2 days (50% conversion). The ee of **3aa** reached a maximum after 4 h and then starts to decrease (Figure 3D). When thiourea **VI** was used as the catalyst, a similar trend was observed, although the conversion after 2 days was lower than 40% (Figure 3E), and the decrease in the enantiopurity of compound **3aa** was slower (Figure 3F). These experiments shown that full conversion is not raised in neither of the two catalysts, while a decrease of the enantiopurity of product **3aa** is observed upon prolonged times. These results are similar to those reported in other aldol reactions with trifluoromethylketones^22^ and indicate the possibility of racemization by a retro-aldol reaction induced by the catalyst as the cause of the moderate yields and enantioselectivities observed.

## Conclusion

In conclusion, we have presented an asymmetric synthesis of trifluoromethyl alcohols bearing a pyrazolone moiety with a tetrasubstituted carbon–carbon double bond through an enantioselective organocatalytic vinylogous aldol reaction of alkylidenepyrazolones with trifluoromethyl ketones catalyzed by a bifunctional organocatalyst. This asymmetric catalytic reaction described here is the first diastereo- and enantioselective vinylogous aldol reaction using alkylidenepyrazolones as nucelophiles. In addition, we have performed the diastereoselective epoxidation of the double bond of the corresponding product **3** that led us to determine the absolute configuration of the aldol products. A detailed reaction monitoring (^1^H NMR and HPLC) showed that full conversion of **1a** is not raised being one of the reasons for the moderate yields, while the enantiomeric excess of products decreases probably due to the existence of a vinylogous retro-aldol reaction induced by the catalyst. Investigations to further study the kinetics and thermodynamics of the reactions as well as the extension of the use of alkylidenepyrazolones in vinylogous aldol reactions are currently underway in our laboratory.

## Experimental Section

### General Methods

Reactions were carried out in 5 mL vials under air. Commercial reagents were used as purchased. Reactions were monitored by TLC analysis using Merck Silica Gel 60 F-254 thin layer plates. Flash column chromatography was performed on Merck silica gel 60, 0.040–0.063 mm, and visualized using both a UV lamp (254 nm) and then a CAM solution (an aqueous solution of ceric ammonium molybdate). Melting points were determined in capillary tubes. NMR spectra were run at 300 MHz for ^1^H and 75 MHz for ^13^C using residual nondeuterated solvent as internal standard (CHCl_3_, δ 7.26 and 77.00 ppm, respectively; MeOH, δ 3.34 ppm and δ 49.87 ppm, respectively). Chemical shifts are given in ppm. The carbon type was determined by DEPT experiments. High-resolution mass spectra (ESI) were recorded on a TripleTOF 5600 spectrometer (AB Sciex, Warrington, U.K.) equipped with an electrospray source with a capillary voltage of 4.5 kV (ESI). Specific optical rotations were measured using sodium light (D line 589 nm). Chiral HPLC analyses were performed in a chromatograph equipped with a UV diode-array detector using columns with chiral stationary phases from Daicel. 2,2,2-Trifluoroacetophenones **2** used were commercial and alkylidenpyrazolones **1** were prepared following a reported procedure.^23^

### General Procedure for the Non-Enantioselective Vinylogous Aldol Reaction (i)

In a 5 mL vial, the corresponding alkylidenepyrazolone **1** (0.1 mmol, 1 equiv) and catalyst 3-((3,5-bis(trifluoromethyl)phenyl)amino)-4-((2-(dimethylamino)ethyl)-amino)-ciclobut-3-e-1,2-dione (4.0 mg, 0.01 mmol, 10 mol %) were dissolved in 1 mL of DCM. To this solution, 2,2,2-trifluoroacetophenone **2** (0.1 mmol, 1 equiv) was added and the reaction mixture was left stirring at room temperature for 5 days. Then, the crude was purified by flash column chromatography using hexane–DCM 60:40 to 40:60 as mobile phase affording the final product **3** as a yellow solid.

### General Procedure for the Enantioselective Vinylogous Aldol Reaction (ii)

In a 5 mL vial, the corresponding alkylidenepyrazolone **1** (0.1 mmol, 1 equiv) and the cinchona alkaloid derived thiourea or squaramide catalyst (**VI**^24^ or **IX**,^25^ 0.005 mmol, 5 mol %) were dissolved in DCM (1 mL). To this solution, 2,2,2-trifluoroacetophenone **2** (0.1 mmol, 1 equiv) was added and the reaction mixture was left stirring at room temperature for 5 days. Then, the crude was purified by flash column chromatography using hexane–DCM 60:40 to 40:60 as mobile phase affording the enantiomerically enriched products **3** as a yellow solid.

### General Procedure for the Enantioselective Vinylogous Aldol Reaction at the 1 mmol Reaction Scale (iii)

In a 25 mL round-bottom flask, alkylidenepyrazolone **1h** (1 mmol, 248.7 mg) and catalyst **VI** (5 mol %, 0.05 mmol, 31.0 mg) were dissolved in DCM (10 mL). To this solution, 2,2,2-trifluoroacetophenone **2a** (1 mmol, 140 μL) was added, and the reaction mixture was left stirring at room temperature for 5 days. Then, the crude solid was purified by flash column chromatography using hexane–DCM 60:40 to 40:60 as mobile phase affording 219.9 mg of product **3ha** (0.52 mmol, 52% yield with 77% ee) as a yellow solid.

### Scope of the Enantioselective Vinylogous Aldol Reaction

#### (*R,Z*)-5-Methyl-2-phenyl-4-(5,5,5-trifluoro-4-hydroxy-4-phenylpentan-2-ylidene)-2,4-dihydro-3*H*-pyrazol-3-one (**3aa**)

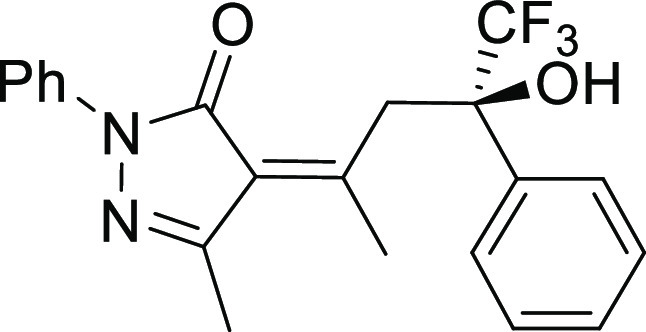



Following general procedure ii and using quinine-derived squaramide **XI** as the catalyst, 17.9 mg of product **3aa** was obtained (46% yield). Enantiomeric excess (77%) was determined by chiral HPLC (Chiralpak ADH *i*PrOH/hexane 10/90, flow rate = 1.0 mL/min, λ = 234 nm), *t*_R_ = 6.79 min (major), *t*_R_ = 5.73 min (minor). [α]_D_^25^ = +314.4 (*c* 0.7, CHCl_3_). Yellow solid, mp = 159–160 °C.

^1^H NMR (300 MHz, CDCl_3_) δ 7.90 (dd, *J* = 8.7, 1.2 Hz, 2H), 7.68 (d, *J* = 6.7 Hz, 2H), 7.49–7.33 (m, 5H), 7.29–7.18 (m, 2H), 6.30 (s, 1H), 4.57 (d, *J* = 12.0 Hz, 1H), 3.01 (d, *J* = 11.9 Hz, 1H), 2.32 (s, 3H), 1.78 (s, 3H). ^19^F NMR (471 MHz, CDCl_3_) δ −79.13. ^13^C {^1^H} NMR (126 MHz, CDCl_3_) δ 165.0 (C), 163.6 (C), 149.1 (C), 137.5 (C), 136.5 (C), 130.1 (C), 128.9 (CH), 128.7 (CH), 128.5 (CH), 126.2 (q, *J*_C–F_ = 1.1 Hz, CH), 125.69 (CH), 125.66 (q, *J*_C–F_ = 287.3 Hz, CF_3_), 119.7 (CH), 78.7 (q, *J*_C–F_ = 28.3 Hz, C), 42.0 (CH_2_), 25.3 (CH_3_), 19.1 (CH_3_). ^19^F NMR (471 MHz, CDCl_3_) δ −79.13 (s, CF_3_). HRMS (ESI/Q-TOF) *m*/*z* [M + H]^+^ C_21_H_20_F_3_N_2_O_2_^+^ calcd for 389.1471; found 389.1458.

#### (*R,Z*)-5-Methyl-2-phenyl-4-(5,5,5-trifluoro-4-hydroxy-4-(*p*-tolyl)pentan-2-ylidene)-2,4-dihydro-3*H*-pyrazol-3-one (**3ab**)

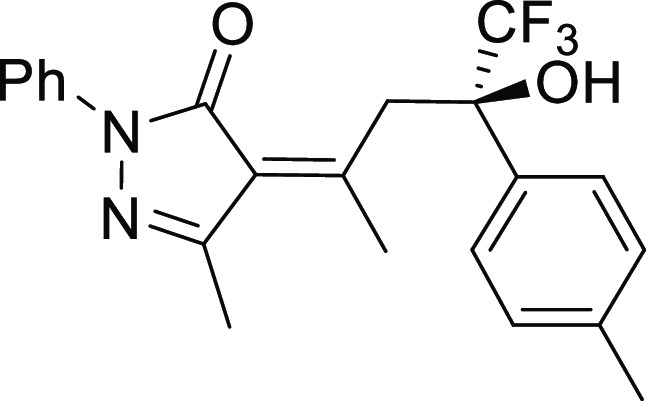



Following general procedure ii and using quinine-derived squaramide **XI** as the catalyst, 12.1 mg of product **3ab** was obtained (30% yield). Enantiomeric excess (65%) was determined by chiral HPLC (Chiralpak ADH *i*PrOH–hexane 10/90, flow rate = 1.0 mL/min, λ = 234 nm), *t*_R_ = 6.62 min (major), *t*_R_ = 5.49 min (minor). [α]_D_^25^ = +310.5 (*c* 0.9, CHCl_3_). Yellow solid, mp = 140–142 °C.

^1^H NMR (300 MHz, CDCl_3_) δ 7.90 (dd, *J* = 8.7, 1.2 Hz, 2H), 7.55 (d, *J* = 8.2 Hz, 2H), 7.47–7.38 (m, 2H), 7.26–7.19 (m, 3H), 6.23 (s, 1H), 4.55 (d, *J* = 11.9 Hz, 1H), 2.99 (d, *J* = 11.9 Hz, 1H), 2.37 (s, 3H), 2.33 (s, 3H), 1.81 (s, 3H). ^13^C {^1^H} NMR (101 MHz, CDCl_3_) δ 165.0 (C), 163.8 (C), 149.1 (C), 138.5 (C), 137.5 (C), 133.5 (C), 130.0 (C), 129.1 (CH), 128.9 (CH), 126.1 (q, *J*_C–F_ = 1.2 Hz, CH), 125.70 (q, *J*_C–F_ = 287.3 Hz, CF_3_), 125.67 (CH), 119.7 (CH), 78.7 (q, *J*_C–F_ = 28.2 Hz, C), 42.0 (CH_2_), 25.5 (CH_3_), 21.1 (CH_3_), 19.1 (CH_3_).^19^F NMR (282 MHz, CDCl_3_) δ −79.34 (s, CF_3_). HRMS (ESI/Q-TOF) *m*/*z* [M + H]^+^ C_22_H_22_F_3_N_2_O_2_^+^ calcd for 403.1628; found 403.1632.

#### (*R,Z*)-4-(4-(4-Chlorophenyl)-5,5,5-trifluoro-4-hydroxypentan-2-ylidene)-5-methyl-2-phenyl-2,4-dihydro-3*H*-pyrazol-3-one (**3ac**)

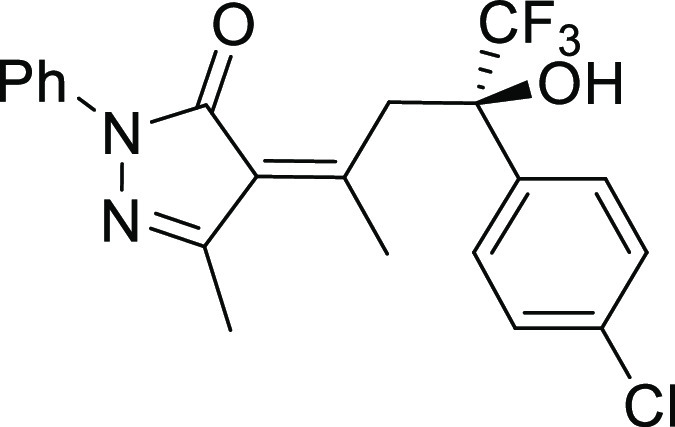



Following the general procedure ii and using as catalyst the quinine-derived squaramide **XI**, 22.4 mg of product **3ac** was obtained (53% yield). Enantiomeric excess (79%) was determined by chiral HPLC (Chiralpak ADH *i*PrOH/hexane 10/90, flow rate = 1.0 mL/min, λ = 234 nm), *t*_R_ = 7.10 min (major), *t*_R_ = 6.55 min (minor). [α]_D_^25^ = +287.7 (*c* 0.9, CHCl_3_). Yellow solid, mp = 142–144 °C.

^1^H NMR (300 MHz, CDCl_3_) δ 7.89 (dd, *J* = 8.7, 1.1 Hz, 2H), 7.63 (d, *J* = 8.6 Hz, 2H), 7.51–7.35 (m, 4H), 7.31–7.19 (m, 1H), 6.41 (s, 1H), 4.54 (d, *J* = 12.1 Hz, 1H), 2.98 (d, *J* = 12.0 Hz, 1H), 2.34 (s, 3H), 1.83 (s, 3H).^13^C {^**1**^H} NMR (75 MHz, CDCl_3_) δ 164.9 (C), 162.8 (C), 149.1 (C), 137.4 (C), 135.2 (C), 134.9 (C), 130.3 (C), 128.9 (CH), 128.7 (CH), 127.8 (q, *J*_C–F_ = 1.5 Hz, CH), 125.8 (CH), 125.4 (q, *J*_C–F_ = 287.3 Hz, CF_3_), 119.7 (CH), 78.5 (q, *J*_C–F_ = 28.4 Hz, C), 41.85 (CH_2_), 25.5 (CH_3_), 19.1 (CH_3_). ^19^F NMR (282 MHz, CDCl_3_) δ −79.24 (s, CF_3_). HRMS (ESI/Q-TOF) *m*/*z* [M + H]^+^ C_21_H_19_ClF_3_N_2_O_2_^+^ calcd for 423.1082; found 423.1084.

#### (*R,Z*)-4-(4-(4-Bromophenyl)-5,5,5-trifluoro-4-hydroxypentan-2-ylidene)-5-methyl-2-phenyl-2,4-dihydro-3*H*-pyrazol-3-one (**3ad**)

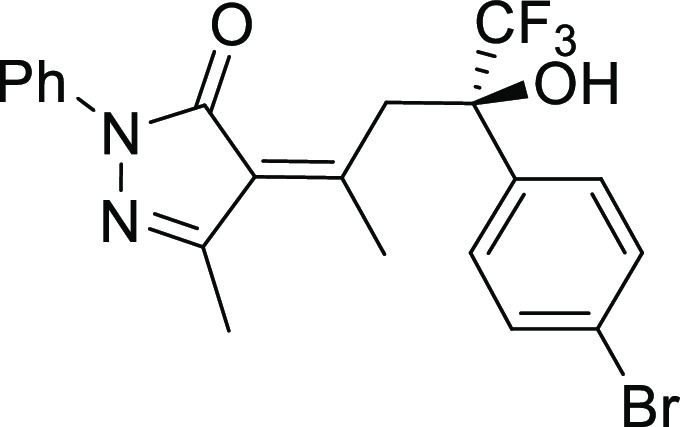



Following the general procedure ii and using the cinchonidine-derived thiourea **VI** as the catalyst, 38.3 mg of product **3ad** was obtained (82% yield). Enantiomeric excess (56%) was determined by chiral HPLC (Chiralpak ADH *i*PrOH/hexane 10/90, flow rate = 1.0 mL/min, λ = 234 nm), *t*_R_ = 7.16 min (major), *t*_R_ = 6.61 min (minor). [α]_D_^25^ = +273.6 (*c* 0.9, CHCl_3_). Yellow solid, mp = 146–147 °C.

^1^H NMR (300 MHz, CDCl_3_) δ 7.89 (dd, *J* = 8.7, 1.2 Hz, 2H), 7.56 (s, 4H), 7.45–7.40 (m, 2H), 7.26–7.20 (m, 1H), 6.41 (s, 1H), 4.53 (d, *J* = 12.1 Hz, 1H), 2.97 (d, *J* = 12.0 Hz, 1H), 2.34 (s, 3H), 1.83 (s, 3H). ^13^C {^1^H} NMR (75 MHz, CDCl_3_) δ 164.9 (C), 162.7 (C), 149.1 (C), 137.4 (C), 135.7 (C), 131.6 (CH), 130.3 (C), 128.9 (CH), 128.1 (q, *J*_C–F_ = 1.2 Hz, CH), 125.8 (CH), 125.4 (q, *J*_C–F_ = 287.4 Hz, CF_3_), 123.1 (C), 119.7 (CH), 78.5 (q, *J*_C–F_ = 28.5 Hz, C), 41.8 (CH_2_), 25.5 (CH_3_), 19.1 (CH_3_). ^19^F NMR (282 MHz, CDCl_3_) δ −79.23 (s, CF_3_). HRMS (ESI/Q-TOF) *m*/*z* [M + H]^+^ C_21_H_19_BrF_3_N_2_O_2_^+^ calcd for 467.0577; found 467.0563.

#### (*R,Z*)-4-(1,1,1-Trifluoro-2-hydroxy-4-(3-methyl-5-oxo-1-phenyl-1,5-dihydro-4*H*-pyrazol-4-ylidene)pentan-2-yl)benzonitrile (**3ae**)

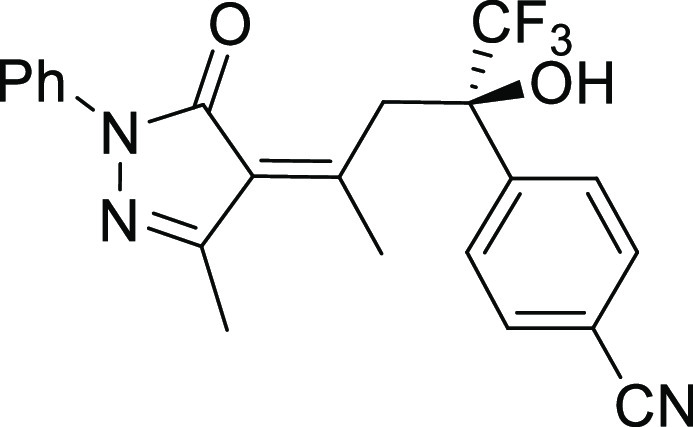



Following general procedure ii and using quinine-derived squaramide **XI** as the catalyst, 23.6 mg of product **3ae** was obtained (57% yield). Enantiomeric excess (87%) was determined by chiral HPLC (Chiralpak ADH *i*PrOH–hexane 10/90, flow rate = 1.0 mL/min, λ = 234 nm), *t*_R_ = 14.21 min (major), *t*_R_ = 12.19 min (minor). [α]_D_^25^ = +193.5 (*c* 0.8, CHCl_3_). Yellow oil.

^1^H NMR (300 MHz, CDCl_3_**)** δ 7.93–7.80 (m, 4H), 7.78–7.67 (m, 2H), 7.47–7.37 (m, 2H), 7.31–7.17 (m, 1H), 6.61 (s, 1H), 4.53 (d, *J* = 12.3 Hz, 1H), 3.03 (d, *J* = 12.2 Hz, 1H), 2.34 (s, 3H), 1.82 (s, 3H). ^13^C {^1^H} NMR (75 MHz, CDCl_3_) δ 164.8 (C), 161.6 (C), 149.0 (C), 142.1 (C), 137.3 (C), 132.2 (CH), 130.6 (C), 128.9 (CH), 127.3 (q, *J*_C–F_ = 1.5 Hz, CH), 125.9 (CH) 125.2 (q, *J*_C–F_ = 287.3 Hz, C), 119.7 (CH), 118.2 (C), 112.9 (C), 78.6 (q, *J*_C–F_ = 28.8 Hz, C), 41.7 (CH_2_), 25.4 (CH_3_), 19.0 (CH_3_). ^19^F NMR (282 MHz, CDCl_3_) δ −78.78 (s, CF_3_). HRMS (ESI/Q-TOF) *m*/*z* [M + H]^+^ C_22_H_19_F_3_N_3_O_2_^+^ calcd for 414.1424; found 414.1419.

#### (*R, Z*)-5-Methyl-2-phenyl-4-(5,5,5-trifluoro-4-hydroxy-4-(3-methoxyphenyl)pentan-2-ylidene)-2,4-dihydro-3*H*-pyrazol-3-one (**3af**)

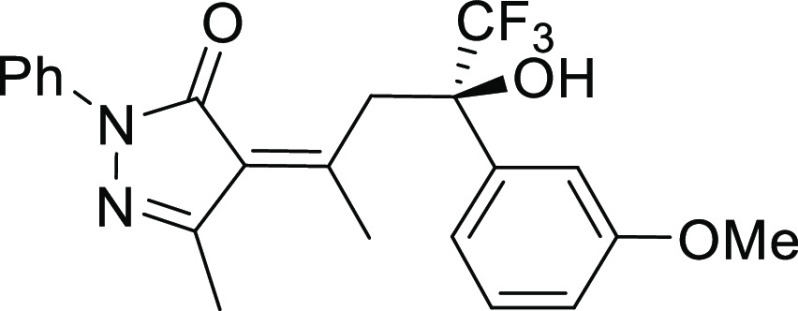



Following general procedure ii and using quinine-derived squaramide **XI** as the catalyst, 22.1 mg of product **3af** was obtained (53% yield). Enantiomeric excess (58%) was determined by chiral HPLC (Chiralpak ADH *i*PrOH–hexane 10/90, flow rate = 1.0 mL/min, λ = 234 nm), *t*_R_ = 9.15 min (major), *t*_R_ = 6.99 min (minor). [α]_D_^25^ = +152.0 (*c* 0.8, CHCl_3_). Yellow oil.

^1^H NMR (300 MHz, CDCl_3_) δ 7.90 (dd, *J* = 8.7, 1.2 Hz, 2H), 7.59 (d, *J* = 8.7 Hz, 2H), 7.46–7.38 (m, 2H), 7.25–7.19 (m, 1H), 6.98–6.91 (m, 2H), 6.23 (s, 1H), 4.55 (d, *J* = 11.9 Hz, 1H), 3.83 (s, 3H), 2.98 (d, *J* = 11.9 Hz, 1H), 2.33 (s, 3H), 1.83 (s, 3H). ^13^C {^1^H} NMR (101 MHz, CDCl_3_) δ 165.0 (C), 163.8 (C), 159.8 (C), 149.1 (C), 137.5 (C), 130.0 (C), 128.9 (CH), 128.4 (C), 127.53 (d, *J* = 298.2 Hz, CF_3_), 127.51 (q, *J*_C–F_ = 1.3 Hz, CH), 125.7 (CH), 119.7 (CH), 113.7 (CH), 78,5 (q, *J*_C–F_ = 28.2 Hz, C), 55.3 (CH_3_), 42.0 (CH_2_), 25.5 (CH_3_), 19.1 (CH_3_). ^19^F NMR (282 MHz, CDCl_3_) δ −79.55 (s, CF_3_). HRMS (ESI/Q-TOF) *m*/*z* [M + H]^+^ C_22_H_22_F_3_N_2_O_3_^+^ calcd for 419.1577; found 419.1581.

#### (*R,Z*)-5-Methyl-2-phenyl-4-(5,5,5-trifluoro-4-hydroxy-4-(*m*-tolyl)pentan-2-ylidene)-2,4-dihydro-3*H*-pyrazol-3-one (**3ag**)

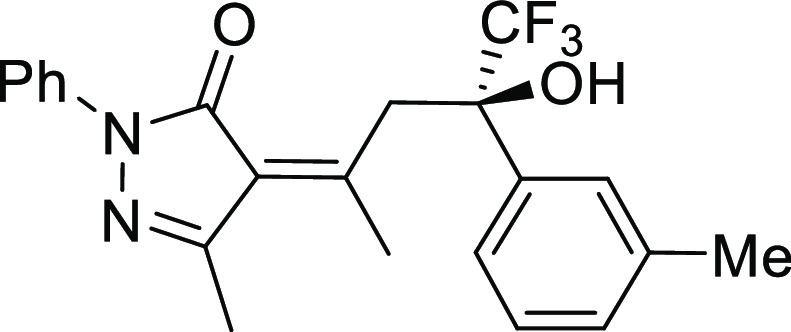



Following general procedure ii and using quinine-derived squaramide **XI** as the catalyst, 18.5 mg of product **3ag** was obtained (46% yield). Enantiomeric excess (80%) was determined by chiral HPLC (Chiralpak ADH *i*PrOH–hexane 10/90, flow rate = 1.0 mL/min, λ = 234 nm), *t*_R_ = 6.41 min (major), *t*_R_ = 5.27 min (minor). [α]_D_^25^ = +293.9 (*c* 0.7, CHCl_3_). Yellow oil.

^1^H NMR (300 MHz, CDCl_3_) δ 7.89 (dd, *J* = 8.7, 1.2 Hz, 2H), 7.53–7.37 (m, 4H), 7.30 (t, *J* = 7.7 Hz, 1H), 7.26–7.15 (m, 2H), 6.26 (s, 1H), 4.55 (d, *J* = 11.9 Hz, 1H), 3.01 (d, *J* = 11.9 Hz, 1H), 2.39 (s, 3H), 2.33 (s, 3H), 1.81 (s, 3H).^13^C {^1^H} NMR (101 MHz, CDCl_3_) δ 165.0 (C), 163.8 (C), 149.1 (C), 138.2 (C), 137.5 (C), 136.4 (C), 130.0 (C), 129.4 (CH), 128.9 (CH), 128.3 (CH), 126.8 (q, *J*_C–F_ = 1.1 Hz, CH), 125.7 (CH), 125.7 (q, *J*_C–F_ = 287.6 Hz, CF_3_), 123.3 (q, *J*_C–F_ = 1.5 Hz, CH), 119.7 (CH), 78.7 (q, *J*_C–F_ = 28.4 Hz, C), 42.0 (CH_2_), 25.4 (CH_3_), 21.6 (CH_3_), 19.1 (CH_3_). ^19^F NMR (282 MHz, CDCl_3_) δ −79.14 (s, CF_3_). HRMS (ESI/Q-TOF) *m*/*z* [M + H]^+^ C_22_H_22_F_3_N_2_O_2_^+^ calcd for 403.1628; found 403.1625.

#### (*R,Z*)-4-(4-(3,4-Dichlorophenyl)-5,5,5-trifluoro-4-hydroxypentan-2-ylidene)-5-methyl-2-phenyl-2,4-dihydro-3*H*-pyrazol-3-one (**3ah**)

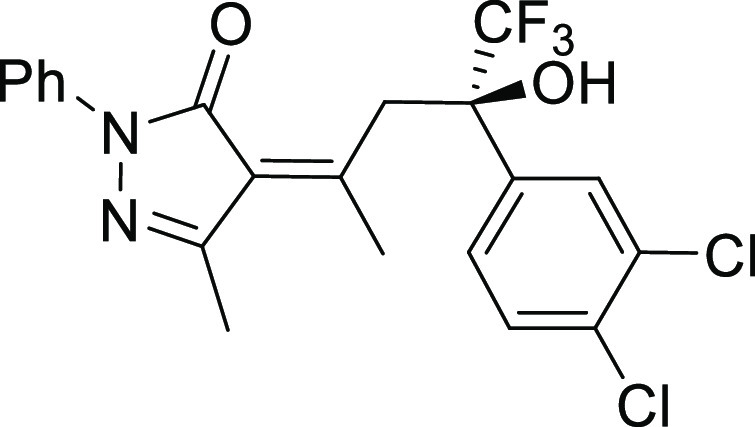



Following general procedure ii and using quinine-derived squaramide **XI** as the catalyst, 27.0 mg of product **3ah** was obtained (59% yield). Enantiomeric excess (84%) was determined by chiral HPLC (Chiralpak ADH *i*PrOH–hexane 10/90, flow rate = 1.0 mL/min, λ = 234 nm), *t*_R_ = 6.95 min (major), *t*_R_ = 6.29 min (minor). [α]_D_^25^ = +234.8 (*c* 0.8, CHCl_3_). Yellow oil.

^1^H NMR (300 MHz, CDCl_3_) δ 7.89 (dd, *J* = 8.7, 1.1 Hz, 2H), 7.81 (s, 1H), 7.51 (s, 2H), 7.47–7.38 (m, 2H), 7.28–7.20 (m, 1H), 6.55 (s, 1H), 4.49 (d, *J* = 12.2 Hz, 1H), 2.99 (d, *J* = 12.2 Hz, 1H), 2.35 (s, 3H), 1.89 (s, 3H). ^13^C {^1^H} NMR (75 MHz, CDCl_3_) δ 164.9 (C), 162.0 (C), 149.1 (C), 137.3 (C), 137.1 (C), 133.2 (C), 133.0 (C), 130.5 (C), 130.4 (CH), 128.9 (CH), 128.8 (q, *J*_C–F_ = 1.0 Hz, CH), 125.9 (CH), 125.7 (q, *J*_C–F_ = 1.5 Hz, CH), 125.2 (q, *J*_C–F_ = 287.3 Hz, CF_3_), 119.73 (CH), 78.2 (q, *J*_C–F_ = 28.8 Hz, C), 41.7 (CH_2_), 25.6 (CH_3_), 19.1 (CH_3_). ^19^F NMR (282 MHz, CDCl_3_) δ −79.16 (s, CF_3_). HRMS (ESI/Q-TOF) *m*/*z* [M + H]^+^ C_21_H_18_Cl_2_F_3_N_2_O_2_^+^ calcd for 457.0692; found 457.0686.

#### (*R,Z*)-5-Methyl-2-phenyl-4-(5,5,5-trifluoro-4-hydroxy-4-(2-methoxyphenyl)pentan-2-ylidene)-2,4-dihydro-3*H*-pyrazol-3-one (**3ai**)

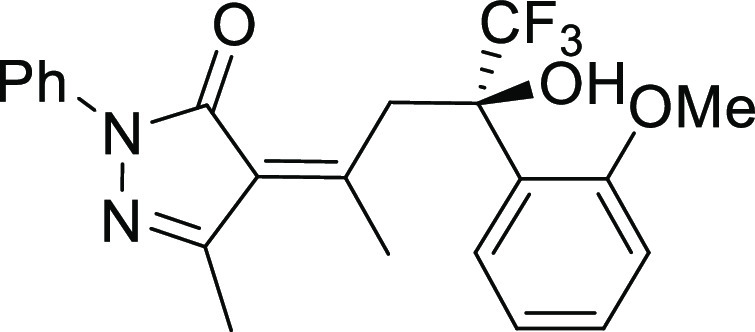



Following general procedure ii and using cinchonidine-derived thiourea **XI** (10 mol %) as the catalyst, 8.4 mg of product **3ai** was obtained (20% yield). Enantiomeric excess (78%) was determined by chiral HPLC (Chiralpak ADH *i*PrOH–hexane 10/90, flow rate = 1.0 mL/min, λ = 234 nm), *t*_R_ = 7.23 min (major), *t*_R_ = 5.88 min (minor). [α]_D_^25^ = +224.9 (*c* 0.4, CHCl_3_). Yellow oil.

^1^H NMR (300 MHz, CDCl_3_) δ 7.91 (dd, *J* = 8.7, 1.2 Hz, 2H), 7.73 (dd, *J* = 7.9, 1.7 Hz, 1H), 7.46–7.32 (m, 3H), 7.27–7.15 (m, 1H), 7.06–6.94 (m, 2H), 6.39 (s, 1H), 4.19 (s, 2H), 3.93 (s, 3H), 2.33 (s, 3H), 2.06 (s, 3H). ^13^C {^1^H} NMR (101 MHz, CDCl_3_) δ 165.7 (C), 164.7 (C), 157.1 (C), 149.0 (C), 140.9 (C), 137.7 (C), 130.4 (CH), 130.0 (CH), 129.4 (C), 128.8 (CH), 125.5 (q, *J*_*C–F*_ = 293.6 Hz, CF_3_), 125.4 (CH), 121.4 (CH), 119.6 (CH), 112.0 (CH), 78.8 (q, *J*_C–F_ = 29.9 Hz, C), 55.7 (CH_3_), 37.6 (CH_2_), 23.6 (CH_3_), 19.3 (CH_3_). ^19^F NMR (282 MHz, CDCl_3_) δ −78.94 (s, CF_3_). HRMS (ESI/Q-TOF) *m*/*z* [M + H]^+^ C_22_H_22_F_3_N_2_O_3_^+^ calcd for 419.1577; found 419.1578.

#### (*S,Z*)-5-Methyl-2-phenyl-4-(5,5,5-trifluoro-4-hydroxy-4-(thiophen-2-yl)pentan-2-ylidene)-2,4-dihydro-3*H*-pyrazol-3-one (**3aj**)

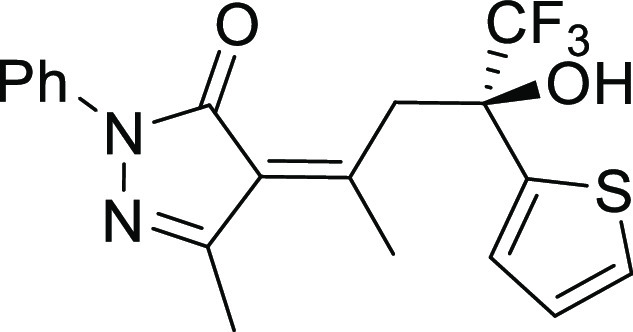



Following general procedure ii and using cinchonidine-derived thiourea **XI** (10 mol %) as the catalyst, 11.8 mg of product **3aj** was obtained (30% yield). Enantiomeric excess (56%) was determined by chiral HPLC (Chiralpak ADH *i*PrOH–hexane 10/90, flow rate = 1.0 mL/min, λ = 234 nm), *t*_R_ = 6.80 min (major), *t*_R_ = 6.15 min (minor). [α]_D_^25^ = +76.8 (*c* 0.6, CHCl_3_). Yellow solid, mp = 167–169 °C.

^1^H NMR (300 MHz, CDCl_3_) δ 7.89 (dd, *J* = 8.7, 1.2 Hz, 2H), 7.48–7.37 (m, 2H), 7.34 (dd, *J* = 5.1, 1.2 Hz, 1H), 7.26–7.17 (m, 2H), 7.07 (dd, *J* = 5.1, 3.7 Hz, 1H), 6.69 (s, 1H), 4.55 (d, *J* = 11.9 Hz, 1H), 2.94 (d, *J* = 11.9 Hz, 1H), 2.37 (s, 3H), 1.89 (s, 3H). ^13^C {^1^H} NMR (75 MHz, CDCl_3_) δ 165.0 (C), 163.2 (C), 149.2 (C), 141.1 (C), 137.4 (C), 130.2 (C), 128.9 (CH), 127.4 (CH), 126.1 (CH), 125.8 (CH), 125.0 (q, *J*_C–F_ = 287.3 Hz, CF_3_), 124.7 (q, *J*_C–F_ = 1.6 Hz, CH), 119.7 (CH), 78.6 (q, *J*_C–F_ = 29.9 Hz, C), 43.0 (CH_2_), 25.2 (CH_3_), 19.0 (CH_3_). ^19^F NMR (282 MHz, CDCl_3_) δ −80.97 (s, CF_3_). HRMS (ESI/Q-TOF) *m*/*z* [M + H]^+^ C_19_H_18_F_3_N_2_O_2_S^+^ calcd for 395.1036; found 395.1028.

#### (*R,Z*)-2,5-diphenyl-4-(5,5,5-trifluoro-4-hydroxy-4-phenylpentan-2-ylidene)-2,4-dihydro-3*H*-pyrazol-3-one (**3ba**)

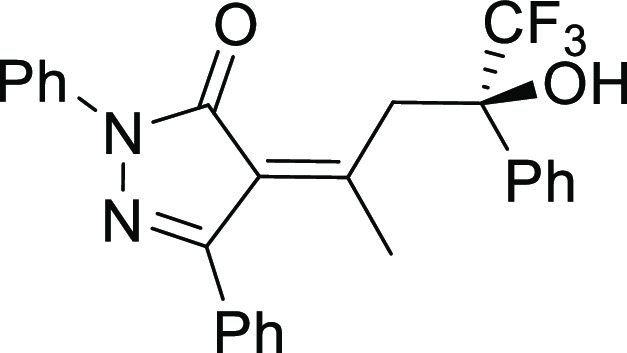



Following general procedure ii and using cinchonidine-derived thiourea **VI** as the catalyst, 20.7 mg of product **3ba** was obtained (46% yield). Enantiomeric excess (68%) was determined by chiral HPLC (Chiralpak ADH *i*PrOH–hexane 10/90, flow rate = 1.0 mL/min, λ = 234 nm), *t*_R_ = 6.81 min (major), *t*_R_ = 5.75 min (minor). [α]_D_^25^ = +170.9 (*c* 0.7, CHCl_3_). Yellow oil.

^1^H NMR (300 MHz, CDCl_3_) δ 7.96 (dd, *J* = 8.8, 1.2 Hz, 2H), 7.71 (d, *J* = 6.9 Hz, 2H), 7.49–7.29 (m, 10H), 7.29–7.21 (m, 1H), 6.36 (s, 1H), 4.52 (d, *J* = 12.1 Hz, 1H), 3.03 (d, *J* = 12.1 Hz, 1H), 1.40 (s, 3H). ^13^C {^1^H} NMR (75 MHz, CDCl_3_) δ 165.3 (C), 164.9 (C), 152.2 (C), 137.6 (C), 136.6 (C), 133.2 (C), 129.6 (CH), 129.2 (C), 128.9 (CH), 128.8 (CH), 128.7 (CH), 128.5 (CH), 128.4 (CH), 126.2 (q, *J*_C–F_ = 1.2 Hz, CH), 126.0 (CH), 125.7 (d, *J*_C–F_ = 287.1 Hz, CF3), 120.0 (CH), 78.52 (q, *J*_C–F_ = 28.5 Hz, C), 42.4 (CH_2_), 27.5 (CH_3_). ^19^F NMR (282 MHz, CDCl_3_) δ −78.86 (s). HRMS (ESI/Q-TOF) *m*/*z* [M + H]^+^ C_26_H_22_F_3_N_2_O_2_^+^ calcd for 451.1628; found 451.1624.

#### (*R,Z*)-2-Phenyl-5-propyl-4-(5,5,5-trifluoro-4-hydroxy-4-phenylpentan-2-ylidene)-2,4-dihydro-3*H*-pyrazol-3-one (**3ca**)

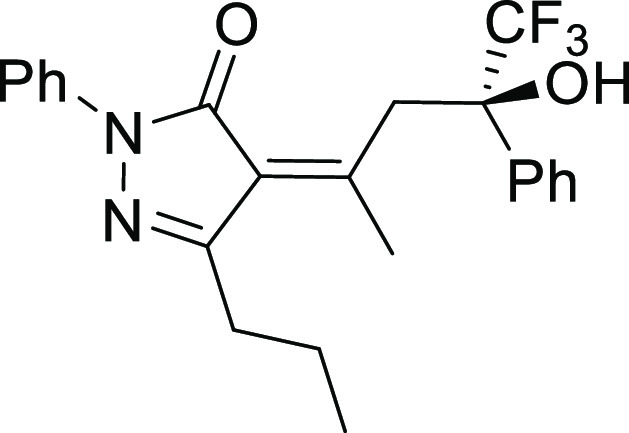



Following general procedure ii and using cinchonidine-derived thiourea **VI** as the catalyst, 23.7 mg of product **3ca** was obtained (57% yield). Enantiomeric excess (76%) was determined by chiral HPLC (Chiralpak ADH *i*PrOH–hexane 10/90, flow rate = 1.0 mL/min, λ = 234 nm), *t*_R_ = 5.15 min (major), *t*_R_ = 4.69 min (minor). [α]_D_^25^ = +429.5 (*c* 0.8, CHCl_3_). Yellow solid, mp = 150–151 °C.

^1^H NMR (300 MHz, CDCl_3_) δ 7.92 (dd, *J* = 8.7, 1.2 Hz, 2H), 7.68 (d, *J* = 6.5 Hz, 2H), 7.55–7.37 (m, 5H), 7.26–7.19 (m, 1H), 6.33 (s, 1H), 4.58 (d, *J* = 12.0 Hz, 1H), 2.98 (d, *J* = 12.0 Hz, 1H), 2.73–2.50 (m, 2H), 1.75 (s, 3H), 1.73–1.35 (m, 2H), 1.00 (t, *J* = 7.4 Hz, 3H). ^13^C {^1^H} NMR (75 MHz, CDCl_3_) δ 165.2 (C), 162.9 (C), 152.4 (C), 137.6 (C), 136.6 (C), 129.7 (C), 128.8 (CH), 128.7 (CH), 128.4 (CH), 126.2 (q, *J* = 1.7 Hz, CH), 125.7 (q, *J* = 286.9 Hz, CF_3_), 125.6 (CH), 119.7 (CH), 78.7 (q, *J* = 28.2 Hz, C), 42.1 (CH_2_), 34.1 (CH_2_), 25.5 (CH_3_), 20.0 (CH_2_), 13.8 (CH_3_). ^19^F NMR (282 MHz, CDCl_3_**)** δ −78.97. HRMS (ESI/Q-TOF) *m*/*z* [M + H]^+^ C_23_H_24_F_3_N_2_O_2_^+^ calcd for 417.1784; found 417.1789.

#### (*R,Z*)-5-Cyclopropyl-2-phenyl-4-(5,5,5-trifluoro-4-hydroxy-4-phenylpentan-2-ylidene)-2,4-dihydro-3*H*-pyrazol-3-one (**3da**)

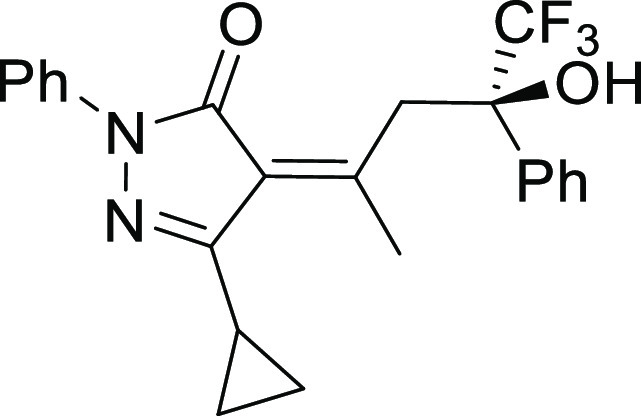



Following general procedure ii and using cinchonidine-derived thiourea **VI** as the catalyst, 27.6 mg of product **3da** was obtained (66% yield). Enantiomeric excess (76%) was determined by chiral HPLC (Chiralpak ADH *i*PrOH–hexane 10/90, flow rate = 1.0 mL/min, λ = 234 nm), *t*_R_ = 4.83 min (major), *t*_R_ = 5.40 min (minor). [α]_D_^25^ = +316.7 (*c* 0.8, CHCl_3_). Yellow solid, mp = 145–146 °C.

^1^H NMR (300 MHz, CDCl_3_) δ 7.90 (dd, *J* = 8.8, 1.1 Hz, 2H), 7.69 (d, *J* = 6.8 Hz, 2H), 7.55–7.35 (m, 5H), 7.28–7.07 (m, 1H), 6.33 (s, 1H), 4.57 (d, *J* = 12.0 Hz, 1H), 3.03 (d, *J* = 11.9 Hz, 1H), 1.96 (s, 3H), 1.78–1.64 (m, 1H), 1.28–1.09 (m, 1H), 0.95–0.82 (m, 3H). ^13^C {^**1**^H} NMR (75 MHz, CDCl_3_) δ 165.1 (C), 163.7 (C), 153.1 (C), 137.6 (C), 136.6 (C), 130.0 (C), 128.8 (CH), 128.7 (CH), 128.4 (CH), 126.2 (q, *J* = 1.7 Hz, CH), 125.7 (q, *J* = 287.5 Hz, CF_3_), 125.6 (CH), 119.6 (CH), 78.7 (q, *J* = 28.2 Hz, C), 42.2 (CH_2_), 25.6 (CH_3_), 12.7 (CH), 7.5 (CH_2_), 6.1 (CH_2_). ^19^F NMR (282 MHz, CDCl_3_) δ −79.05. HRMS (ESI/Q-TOF) *m*/*z* [M + H]^+^ C_23_H_22_F_3_N_2_O_2_^+^ calcd for 415.1628; found 415.1625.

#### (*R,E*)-5-Methyl-2-phenyl-4-(4,4,4-trifluoro-3-hydroxy-1,3-diphenylbutylidene)-2,4-dihydro-3*H*-pyrazol-3-one (**3ea**)

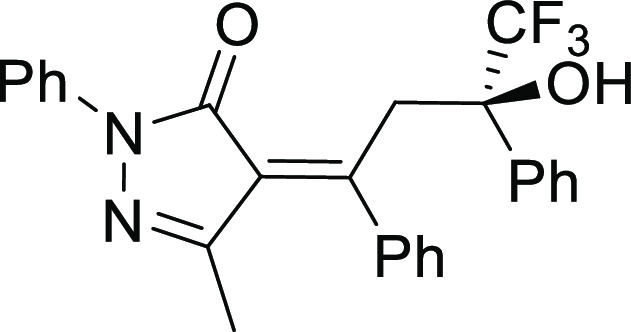



Following general procedure ii and using cinchonidine-derived thiourea **VI** as the catalyst, 21.6 mg of product **3ea** was obtained (48% yield). Enantiomeric excess (94%) was determined by chiral HPLC (Chiralpak IC *i*PrOH–hexane 10/90, flow rate = 1.0 mL/min, λ = 234 nm), *t*_R_ = 5.17 min (major), *t*_R_ = 6.94 min (minor). [α]_D_^25^ = +699.0 (*c* 0.5, CHCl_3_). Yellow solid, mp = 154–155 °C.

^1^H NMR (300 MHz, CDCl_3_) δ 7.93 (dd, *J* = 8.7, 1.2 Hz, 2H), 7.53–7.39 (m, 2H), 7.33 (t, *J* = 7.6 Hz, 1H), 7.27–7.23 (m, 3H), 7.19 (tt, *J* = 7.5, 1.1 Hz, 1H), 7.13 (d, *J* = 7.7 Hz, 1H), 7.09–7.03 (m, 1H), 6.97 (t, *J* = 7.3 Hz, 2H), 6.80 (t, *J* = 7.6 Hz, 1H), 6.69 (s, 1H, OH), 6.20 (d, *J* = 7.8 Hz, 1H), 4.86 (d, *J* = 11.9 Hz, 1H), 3.42 (d, *J* = 11.8 Hz, 1H), 1.42 (s, 3H). ^13^C {^1^H} NMR (75 MHz, CDCl_3_) δ 165.0 (C), 163.5 (C), 149.7 (C), 140.0 (C), 137.5 (C), 134.9 (C), 130.7 (C), 129.2 (CH), 128.9 (CH), 128.3 (CH), 128.0 (CH), 127.6 (CH), 126.5 (q, *J* = 1.7 Hz, CH), 125.8 (CH), 125.52 (q, *J* = 287.5 Hz, CF_3_), 125.5 (CH), 119.7 (CH), 79.0 (q, *J* = 28.2 Hz, C), 41.4 (CH_2_), 17.4 (CH_3_). ^19^F NMR (282 MHz, CDCl_3_) δ −80.52. HRMS (ESI/Q-TOF) *m*/*z* [M + H]^+^ C_26_H_22_F_3_N_2_O_2_^+^ calcd for 451.1628; found 451.1631.

#### (*R,Z*)-2,5-Dimethyl-4-(5,5,5-trifluoro-4-hydroxy-4-phenylpentan-2-ylidene)-2,4-dihydro-3*H*-pyrazol-3-one (**3fa**)

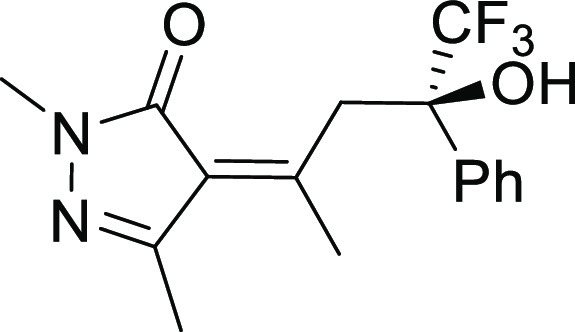



Following general procedure ii and using quinine-derived squaramide **XI** as the catalyst, 17.3 mg of product **3fa** was obtained (53% yield). Enantiomeric excess (92%) was determined by chiral HPLC (Chiralpak ADH *i*PrOH–hexane 10/90, flow rate = 1.0 mL/min, λ = 234 nm), *t*_R_ = 7.05 min (major), *t*_R_ = 5.99 min (minor). [α]_D_^25^ = +223.0 (*c* 0.6, CHCl_3_). Yellow oil.

^1^H NMR (300 MHz, CDCl_3_) δ 7.66 (dd, J = 7.5, 1.4 Hz, 2H), 7.50–7.28 (m, 3H), 6.61 (s, 1H), 4.47 (d, *J* = 11.9 Hz, 1H), 3.36 (s, 3H), 2.94 (d, *J* = 11.9 Hz, 1H), 2.21 (s, 3H), 1.72 (s, 3H). ^13^C {^1^H} NMR (75 MHz, CDCl_3_) δ ^13^C NMR (75 MHz, CDCl_3_) δ 166.1 (C), 162.8 (C), 147.9 (C), 136.6 (C), 129.4 (C), 128.6 (CH), 128.4 (CH), 126.2 (q, *J*_C–F_ = 1.7 Hz, CH), 125.7 (q, *J*_C–F_ = 287.5 Hz, C), 78.8 (q, *J*_C–F_ = 28.2 Hz, C), 42.1 (CH_2_), 31.5 (CH_3_), 25.3 (CH_3_), 19.0 (CH_3_). ^19^F NMR (282 MHz, CDCl_3_) δ −79.21 (s, CF_3_). HRMS (ESI/Q-TOF) *m*/*z* [M + H]^+^ C_16_H_18_F_3_N_2_O_2_^+^ calcd for 327.1315; found 327.1325.

#### (*R,Z*)-2-(4-Methoxyphenyl)-5-methyl-4-(5,5,5-trifluoro-4-hydroxy-4-phenylpentan-2-ylidene)-2,4-dihydro-3*H*-pyrazol-3-one (**3ga**)

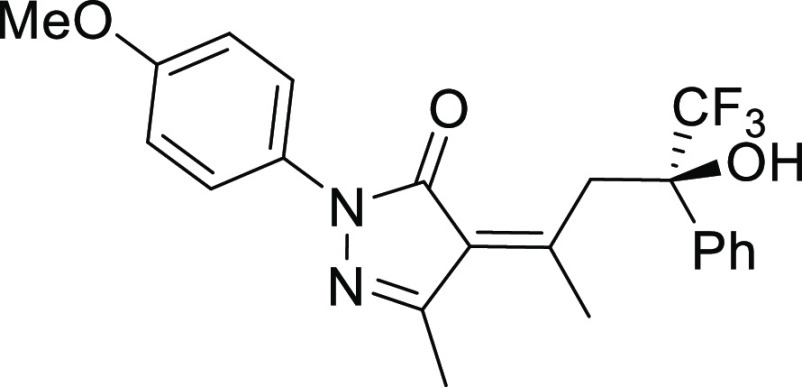



Following general procedure ii and using cinchonidine-derived thiourea **VI** as the catalyst, 22.3 mg of product **3ga** was obtained (53% yield). Enantiomeric excess (66%) was determined by chiral HPLC (Chiralpak ADH *i*PrOH–hexane 10/90, flow rate = 1.0 mL/min, λ = 234 nm), *t*_R_ = 10.81 min (major), *t*_R_ = 8.64 min (minor). [α]_D_^25^ = +328.2 (*c* 0,7, CHCl_3_). Yellow solid, mp = 165–167 °C.

^1^H NMR (300 MHz, CDCl_3_) δ 7.69 (d, *J* = 9.2 Hz, 2H), 7.61 (d, *J* = 6.6 Hz, 2H), 7.40–7.27 (m, 3H), 6.88 (d, *J* = 9.2 Hz, 2H), 6.34 (s, 1H), 4.49 (d, *J* = 12.1 Hz, 1H), 3.76 (s, 3H), 2.93 (d, *J* = 11.9 Hz, 1H), 2.24 (s, 3H), 1.71 (s, 3H). ^13^C {^1^H} NMR (75 MHz, CDCl_3_) δ 164.6 (C), 163.4 (C), 157.5 (C), 148.9 (C), 136.6 (C), 130.8 (C), 130.1 (C), 128.7 (CH), 128.4 (CH), 126.2 (q, *J* = 1.7 Hz, CH), 125.7 (q, *J* = 287.5 Hz, CF_3_), 121.7 (CH), 114.1 (CH), 78.7 (q, *J* = 28.2 Hz, C), 55.5 (CH_3_), 42.0 (CH_2_), 25.3 (CH_3_), 19.0 (CH_3_). ^19^F NMR (282 MHz, CDCl_3_) δ −79.11. HRMS (ESI/Q-TOF) *m*/*z* [M + H]^+^ C_22_H_22_F_3_N_2_O_3_^+^ calcd for 419.1577; found 419.1574.

#### (*R,Z*)-2-(4-Chlorophenyl)-5-methyl-4-(5,5,5-trifluoro-4-hydroxy-4-phenylpentan-2-ylidene)-2,4-dihydro-3*H*-pyrazol-3-one (**3ha**)

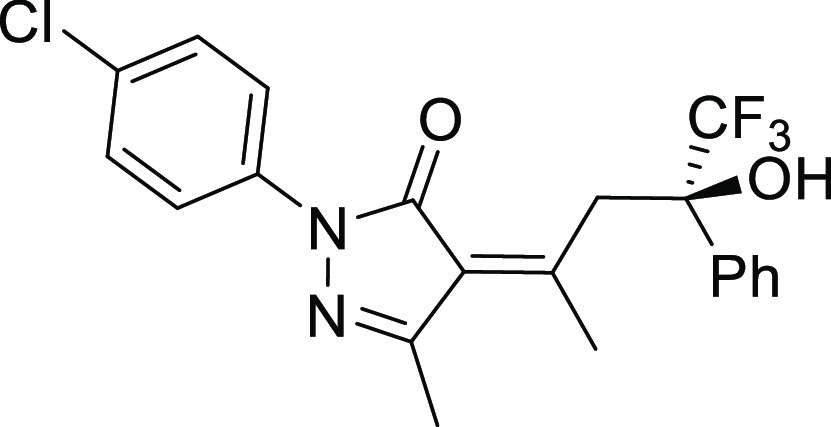



Following general procedure ii and using cinchonidine-derived thiourea **VI** as the catalyst, 24.1 mg of product **3ha** was obtained (57% yield). Enantiomeric excess (74%) was determined by chiral HPLC (Chiralpak ADH *i*PrOH–hexane 10/90, flow rate = 1.0 mL/min, λ = 234 nm), *t*_R_ = 9.16 min (major), *t*_R_ = 6.85 min (minor). [α]_D_^25^ = +337.5 (*c* 0.7, CHCl_3_). Yellow solid, mp = 166–167 °C.

^1^H NMR (300 MHz, CDCl_3_) δ 7.90 (d, *J* = 9.1 Hz, 2H), 7.67 (d, *J* = 6.4 Hz, 2H), 7.51–7.30 (m, 5H), 6.16 (s, 1H), 4.55 (d, *J* = 11.9 Hz, 1H), 3.01 (d, *J* = 11.9 Hz, 1H), 2.32 (s, 3H), 1.78 (s, 3H). ^13^C {^1^H} NMR (75 MHz, CDCl_3_) δ 164.9 (C), 164.2 (C), 149.4 (C), 136.4 (C), 136.1 (C), 130.8 (C), 129.9 (C), 128.9 (CH), 128.8 (CH), 128.5 (CH), 126.15 (d, *J* = 1.1 Hz, CH), 125.61 (q, *J* = 287.5 Hz, CF_3_), 120.6 (CH), 78.7 (q, *J* = 28.5 Hz, C), 42.0 (CH_2_), 25.4 (CH_3_), 19.1 (CH_3_). ^19^F NMR (282 MHz, CDCl_3_) δ −79.18. HRMS (ESI/Q-TOF) *m*/*z* [M + H]^+^ C_21_H_19_ClF_3_N_2_O_2_^+^ calcd for 423.1082; found 423.1086.

#### (*R, Z*)-2-(3,4-Dimethylphenyl)-5-methyl-4-(5,5,5-trifluoro-4-hydroxy-4-phenylpentan-2-ylidene)-2,4-dihydro-3*H*-pyrazol-3-one (**3ia**)

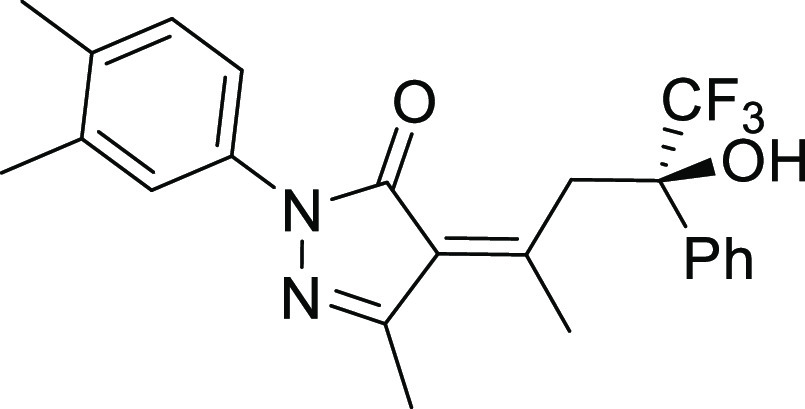



Following general procedure ii and using cinchonidine-derived thiourea **VI** as the catalyst, 22.5 mg of product **3ia** was obtained (54% yield). Enantiomeric excess (77%) was determined by chiral HPLC (Chiralpak ADH *i*PrOH–hexane 10/90, flow rate = 1.0 mL/min, λ = 234 nm), *t*_R_ = 7.49 min (major), *t*_R_ = 5.93 min (minor). [α]_D_^25^ = +346.6 (*c* 0.8, CHCl_3_). Yellow solid, mp = 184–185 °C.

^1^H NMR (300 MHz, CDCl_3_) δ 7.68 (d, *J* = 7.1 Hz, 2H), 7.64–7.56 (m, 2H), 7.48–7.36 (m, 3H), 7.17 (d, *J* = 8.2 Hz, 1H), 6.41 (s, 1H), 4.55 (d, *J* = 11.9 Hz, 1H), 3.00 (d, *J* = 11.9 Hz, 1H), 2.31 (m, 6H), 2.27 (s, 3H), 1.77 (s, 3H). ^13^C {^1^H} NMR (75 MHz, CDCl_3_) δ 164.7 (C), 163.2 (C), 148.9 (C), 137.2 (C), 136.6 (C), 135.2 (C), 134.3 (C), 131.4 (C), 129.9 (CH), 128.7 (CH), 128.4 (CH), 126.2 (q, *J* = 1.1 Hz, CH), 125.7 (q, *J* = 287.5 Hz, CF_3_), 121.1 (CH), 117.5 (CH), 78.7 (q, *J* = 28.5 Hz, C), 42.0 (CH_2_), 25.3 (CH_3_), 20.0 (CH_3_), 19.3 (CH_3_), 19.0 (CH_3_). ^19^F NMR (282 MHz, CDCl_3_) δ −79.08. HRMS (ESI/Q-TOF) *m*/*z* [M + H]^+^ C_23_H_24_F_3_N_2_O_2_^+^ calcd for 417.1784; found 417.1788.

### Procedure and Characterization Data for Compounds **4**

A solution of **3ha** (29.6 mg, 0.07 mmol, 1 equiv) in DCM (1 mL) was cooled to 0 °C in an ice-bath, and mCPBA (22.6 mg, 0.105 mmol, 1.5 equiv) was added dropwise followed by the slow addition of K_2_CO_3_ (14.5 mg, 0.105 mmol, 1.5 equiv). The reaction mixture was stirred at 0 °C for 2 h and then extracted with 10 mL of H_2_O and 3 × 20 mL of DCM. The combined organic layers were dried over MgSO_4_ (anhydrous), and solvent was removed under reduced pressure. The residue was purified by column chromatography being eluted with hexane–DCM 60:40 to hexane–DCM 40:60, obtaining 30.3 mg of product **4** (both diastereoisomers) (98% yield) as yellow solids.

### Major Diastereoisomer **4′**

#### (2*S*,3*S*)-5-(4-Chlorophenyl)-2,7-dimethyl-2-((*R*)-3,3,3-trifluoro-2-hydroxy-2-phenylpropyl)-1-oxa-5,6-diazaspiro[2.4]hept-6-en-4-one

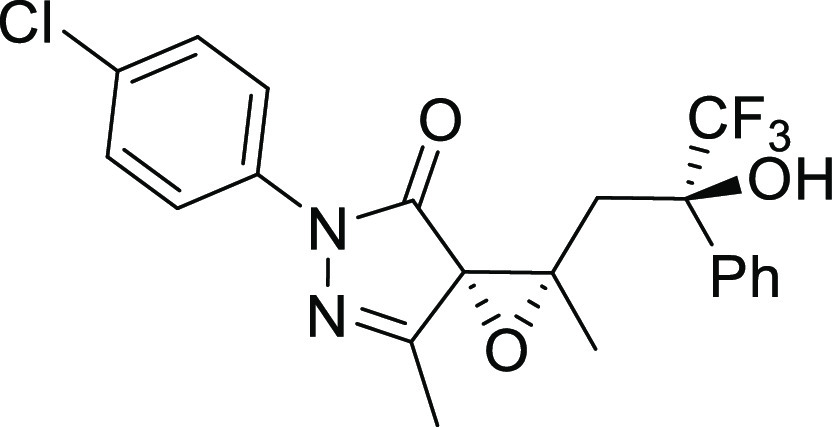



Enantiomeric excess (ee_major_ = 78%) was determined by chiral HPLC (Chiralpak ADH connected in series with Phenomenex Amylose-1 *i*PrOH–hexane 10/90, flow rate = 1.0 mL/min, λ = 234 nm), *t*_R_ = 38.70 min (major diastereoisomer, major enantiomer), *t*_R_ = 54.24 min (major diastereoisomer minor enantiomer), [α]_D_^25^_major_ = +155.0 (*c* 0.9, CHCl_3_). Yellow solid, mp_major_ = 194–195 °C.

^1^H NMR (300 MHz, CDCl_3_) δ 7.88 (d, *J* = 9.2 Hz, 2H), 7.61 (d, *J* = 7.2 Hz, 2H), 7.48–7.32 (m, 5H), 3.35–3.23 (m, 2H), 2.93 (d, *J* = 14.7 Hz, 1H), 2.11 (s, 3H), 1.25 (s, 3H). ^13^C {^1^H} NMR (75 MHz, CDCl_3_) δ 168.8 (C), 156.7 (C), 136.4 (C), 136.1 (C), 130.9 (C), 129.0 (CH), 128.8 (CH), 128.4 (CH), 126.20 (q, *J* = 1.1 Hz, CH), 120.0 (CH), 76.41 (q, *J* = 28.7 Hz, C), 69.1 (C), 65.5 (C), 37.6 (CH_2_), 22.2 (CH_3_), 16.9 (CH_3_). ^19^F NMR (282 MHz, CDCl_3_**)** δ −79.75. HRMS (ESI/Q-TOF) *m*/*z* [M + H]^+^ C_21_H_19_ClF_3_N_2_O_3_^+^ calcd for 439.1031; found 439.1038.

### Minor Diastereoisomer **4′**

#### (2*R*,3*R*)-5-(4-Chlorophenyl)-2,7-dimethyl-2-((*R*)-3,3,3-trifluoro-2-hydroxy-2-phenylpropyl)-1-oxa-5,6-diazaspiro[2.4]hept-6-en-4-one

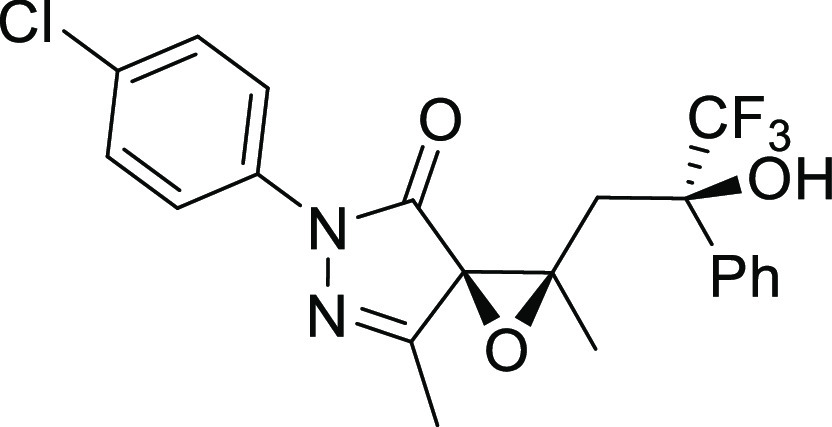



Enantiomeric excess (ee_minor_ = 76%) was determined by chiral HPLC (Chiralpak ADH connected in series with Phenomenex Amylose-1 *i*PrOH–hexane 10/90, flow rate = 1.0 mL/min, λ = 234 nm), *t*_R_ = 46.81 min (minor diastereoisomer major enantiomer), *t*_R_ = 42.58 min (minor diastereoisomer minor enantiomer). [α]_D_^25^_minor_ = −15.8 (*c* 0.2, CHCl_3_).

^1^H NMR (300 MHz, CDCl_3_) δ 7.79 (d, *J* = 9.2 Hz, 2H), 7.65 (d, *J* = 7.6 Hz, 2H), 7.39–7.23 (m, 5H), 4.75 (s, 1H), 2.99 (d, *J* = 15.1 Hz, 1H), 2.71 (d, *J* = 15.2 Hz, 1H), 2.01 (s, 3H), 1.34 (s, 3H). ^19^F NMR (282 MHz, CDCl_3_) δ −80.27.

## Supporting Information Available

^1^H and ^13^C NMR spectra, HPLC chromatograms for all compounds, and crystallographic data for epoxide **4** major diastereoisomer (PDF)
FAIR data, including the primary NMR FID files, for compounds **3aa**–**3aj**, **3ba**–**3ia**, epoxide **4** major diastereoisomer, and epoxide **4** minor diastereoisomer (ZIP)

## Accession Codes

CCDC 2122953 contains the supplementary crystallographic data for this paper. These data can be obtained free of charge via www.ccdc.cam.ac.uk/data_request/cif, or by emailing data_request@ccdc.cam.ac.uk, or by contacting The Cambridge Crystallographic Data Centre, 12 Union Road, Cambridge CB2 1EZ, UK; fax: +44 1223 336033.
