# Supplementary material for: Catalytic Diastereo- and Enantioselective Synthesis of Tertiary Trifluoromethyl Carbinols through a Vinylogous Aldol Reaction of Alkylidenepyrazolones with Trifluoromethyl Ketones
Source: J Org Chem. 2022 Mar 16;87(7):4538–49. doi: 10.1021/acs.joc.1c02817 (PMC8981347; doi:10.1021/acs.joc.1c02817)
Supplement: Supplementary file 1 — jo1c02817_si_001.pdf [file jo1c02817_si_001.pdf]

Supporting information

Catalytic Diastereo- and Enantioselective Synthesis of Tertiary Trifluoromethyl Carbinols through a Vinylogous Aldol Reaction of Alkylidenepyrazolones with Trifluoromethyl ketones.

Laura Carceller-Ferrer,[a] Aleix González del Campo,[a] Carlos Vila,[a] Gonzalo Blay,[a]* M. Carmen Muñoz[b] and José R. Pedro[a]*

a. Departament de Química Orgànica, Facultat de Química, Universitat de València, Dr. Moliner 50, 46100 Burjassot, València (Spain).E-mail: carlos.vila@uv.es, jose.r.pedro@uv.es

b. Departament de Física Aplicada, Universitat Politècnica de València, Camino de Vera s/n, 46022 València (Spain)

Contents

1. Catalysts Screening………………………………………………………………………… S1
2. NOESY experiments with product **3aa**………………………………………………… S2
3. NMR of the crude reaction mixture……………………………………………………….. S4
4. Studies about stability of the aldol adduct **3aa**…………………………………….. S5
5. Conversion of **1a** to obtain **3aa** and ee of **3aa** using **IX** (5 mol%) in $CDCl_3$. S6
6. Conversion of **1a** to obtain **3aa** and ee of **3aa** using **VI** (5 mol%) in $CDCl_3$. S7
7. NMR data ………………………………………………………………………………………….S8
8. HPLC data ………………………………………………………………………………………….S47

1. Catalysts Screening

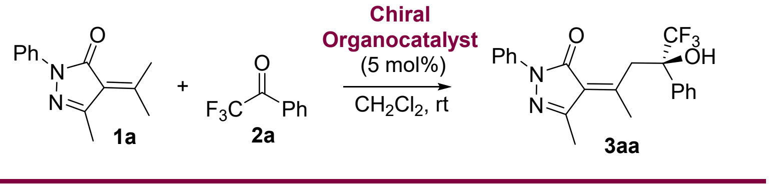

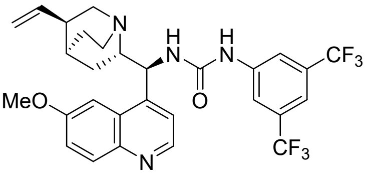

4 days, 58% yield, 36% ee

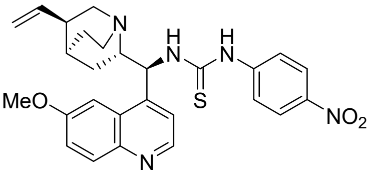

3 days, 46% yield, 67% ee

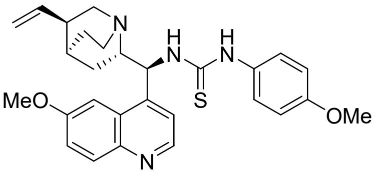

4 days, 29% yield, 81% ee

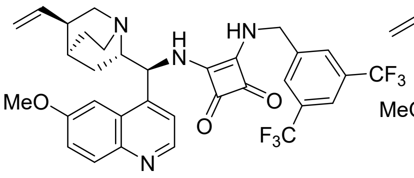

4 days, 52% yield, 50% ee

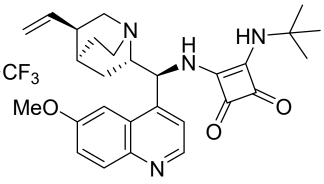

4 days, 44% yield, 60% ee

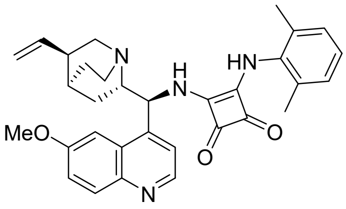

5 days, 24% yield, 82% ee

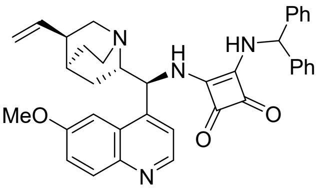

3 days, 38% yield, 65% ee

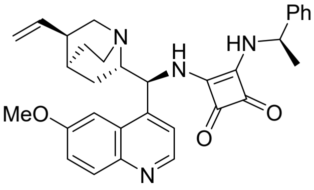

3 days, 34% yield, 65% ee

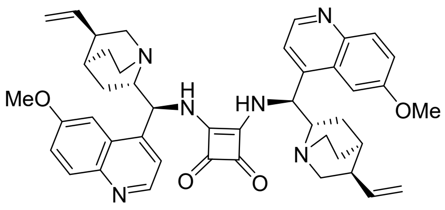

5 days, 45% yield, 59% ee

2. NOESY experiments with product 3aa

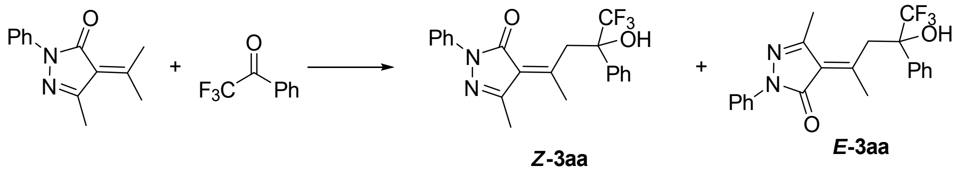

Scheme S1. Possible Z and E isomers in the vinylogous aldol reaction

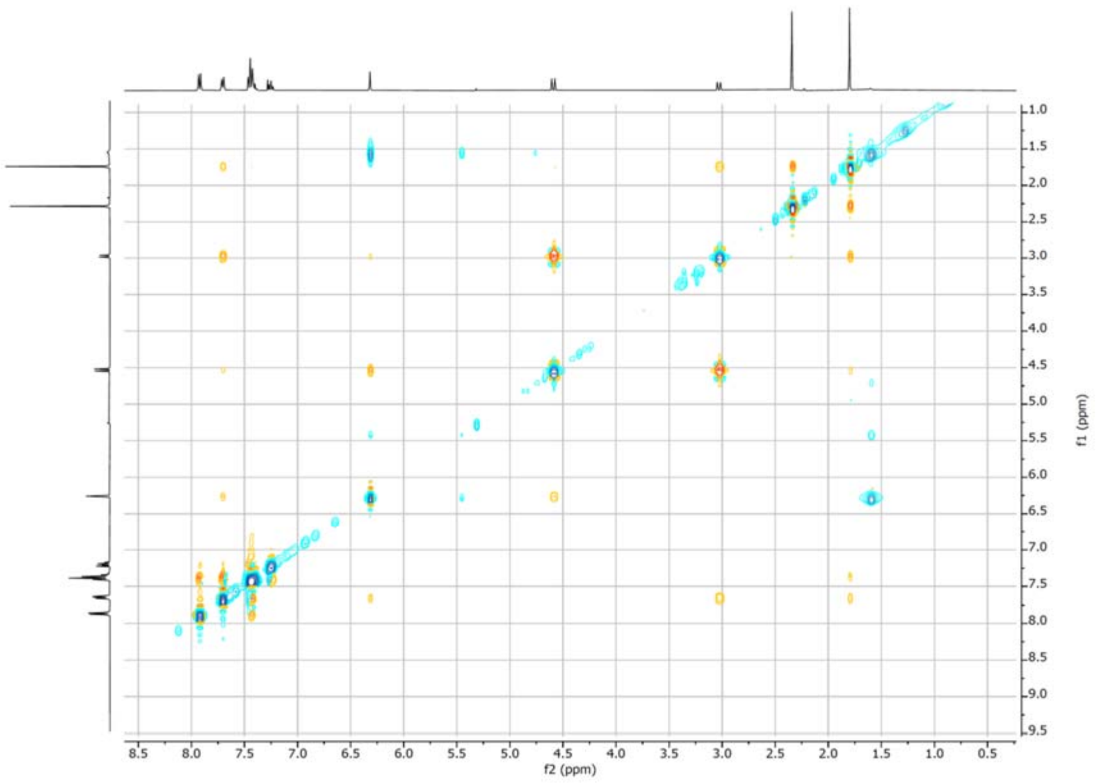

Figure S1. NOESY experiment of product **3aa**

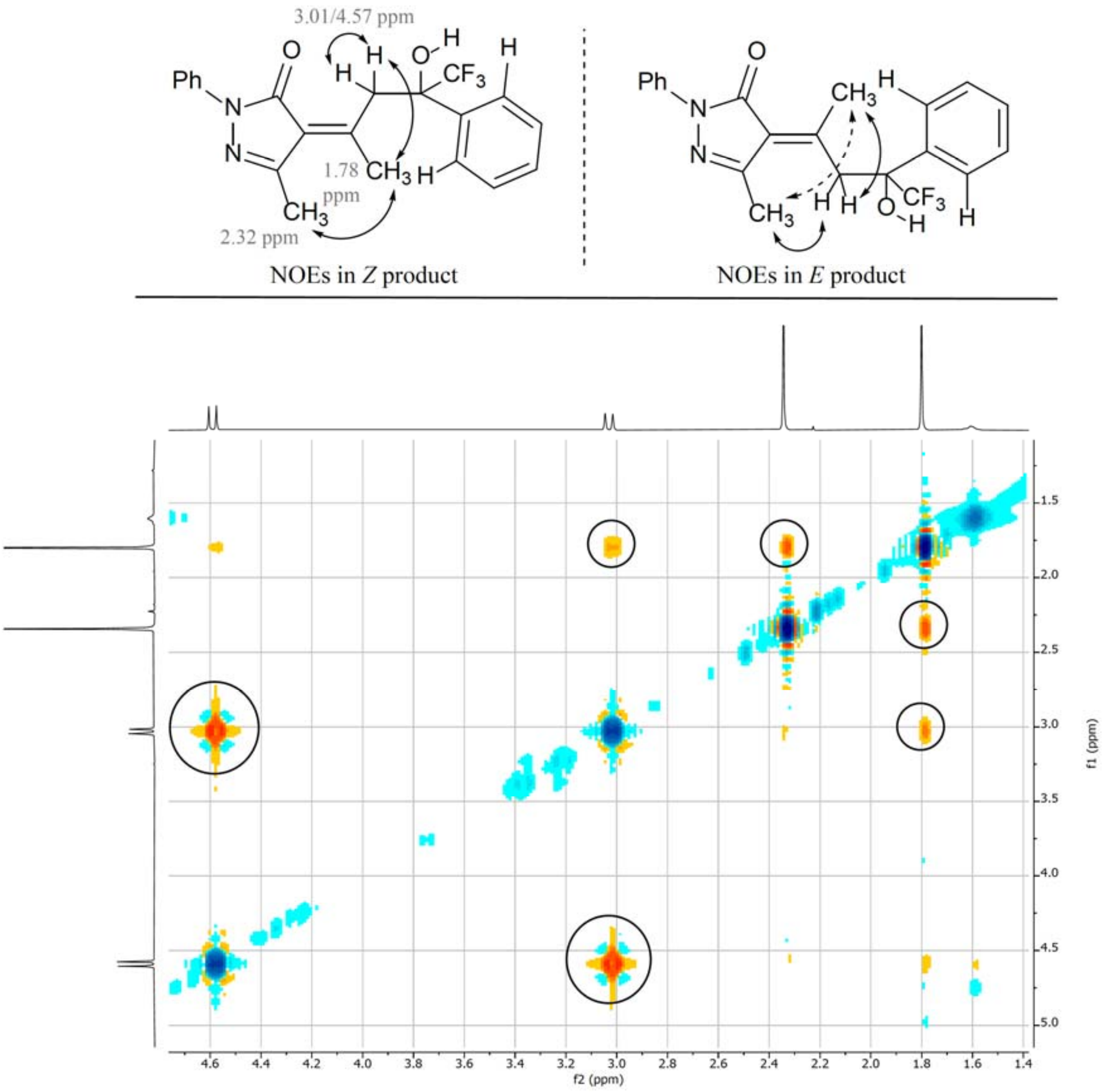

Figure S2. Noesy experiment of product **3aa**

3. NMR of the crude reaction mixture

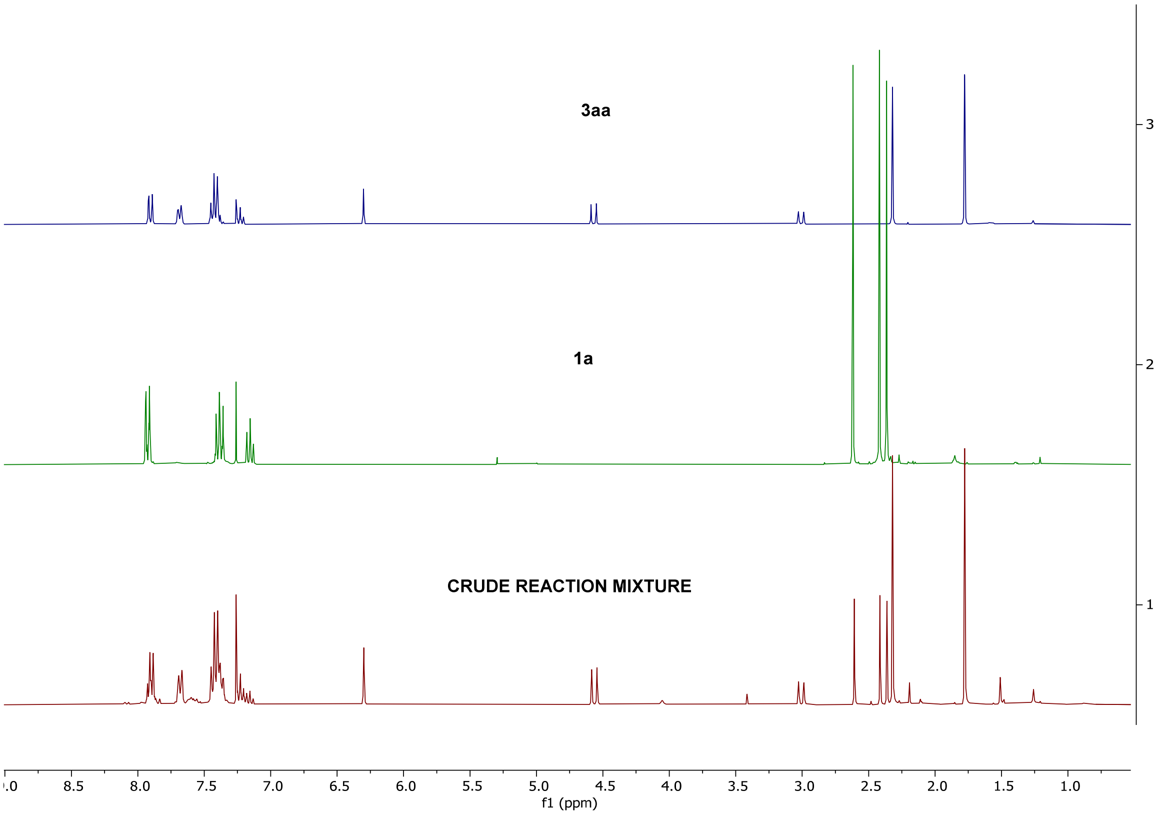

Figure S3. ^{1}H-NMR comparison of the crude reaction mixture, alcohol **3aa** and α-isopropylidenepyrazolone (**1a**).

4. Studies about stability of the aldol adduct 3aa.

4.1 Sample of 3aa (83% ee) stirred in CH_2Cl_2

t(h)	ee
0	83
8	83
24	84
32	85
48	85
56	84
72	86

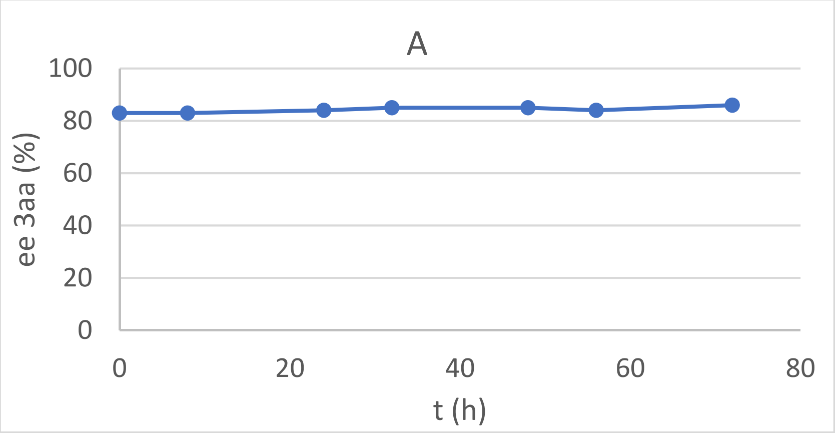

4.2 Sample of **3aa** (73% ee) with 5 mol% of catalyst VI stirred in CH_2Cl_2

t(h)	ee
0	73
8	74
24	70
32	66
48	64
56	62
72	58

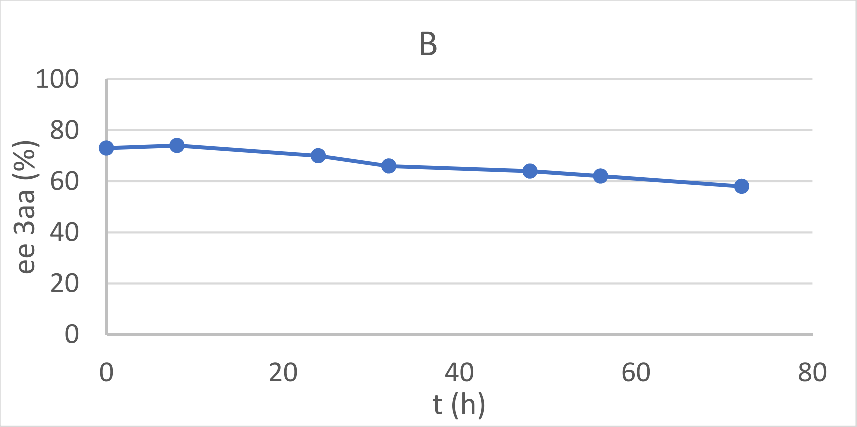

5. Conversion of 1a to obtain 3aa and ee of 3aa using IX (5 mol%) in $CDCl_3$.

t(h)	Conversión (%)	Ee
0	0	0
1	5	28
2	14	91
3	20	92
4	27	92
5	30	89
32	50	79
56	50	74

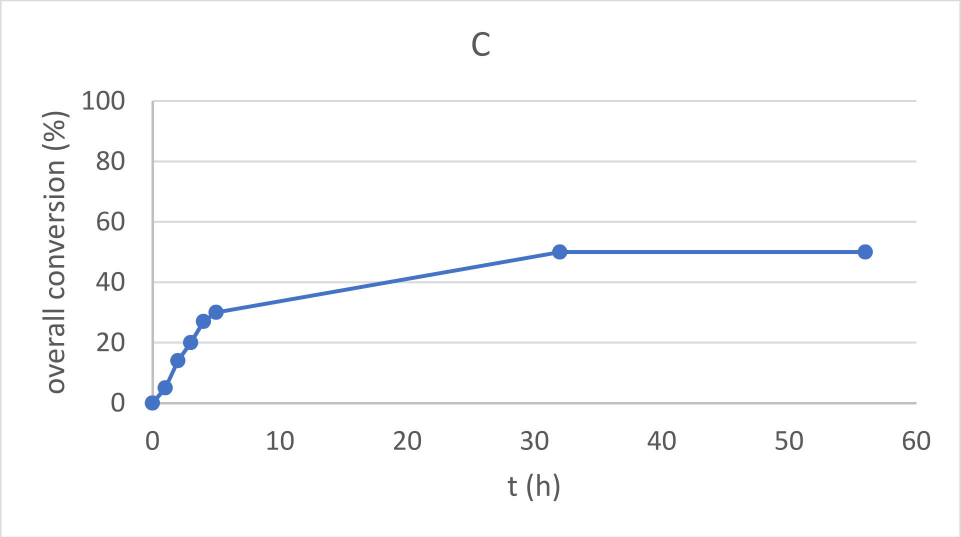

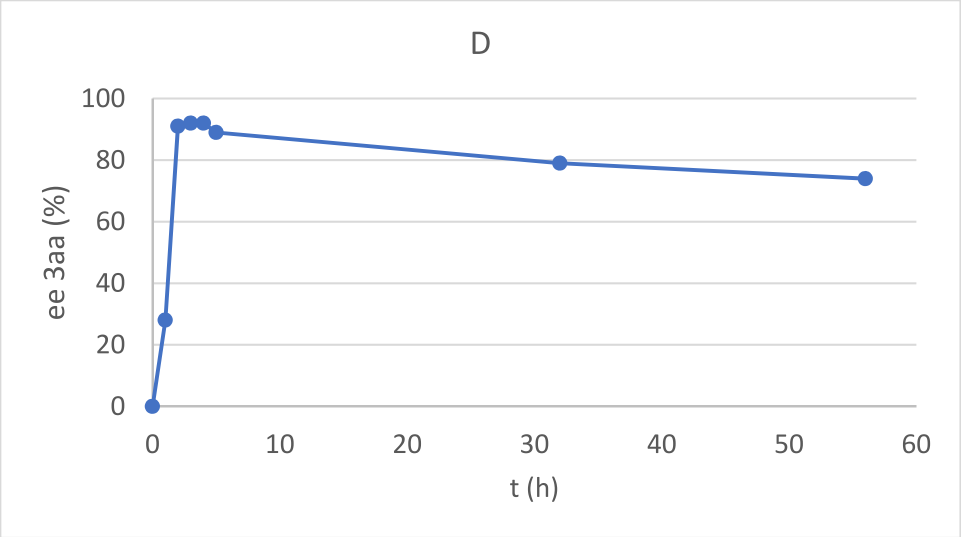

6. Conversion of 1a to obtain 3aa and ee of 3aa using VI (5 mol%) in $CDCl_3$

t(h)	Conversión (%)	ee
0	0	0
1	1	86
2	2	90
3	3	88
4	4	88
5	4	91
6	6	90
8	8	90
32	33	87
56	37	84

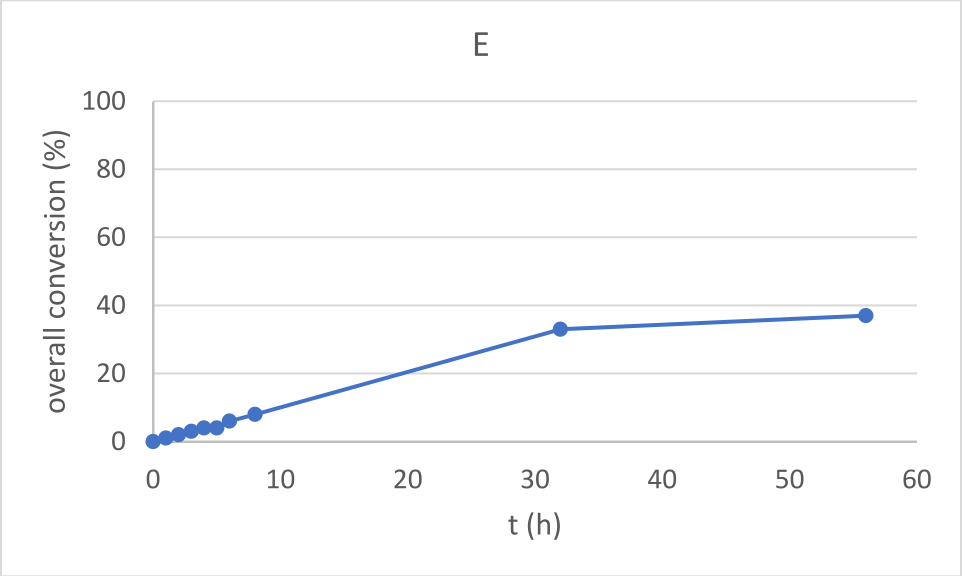

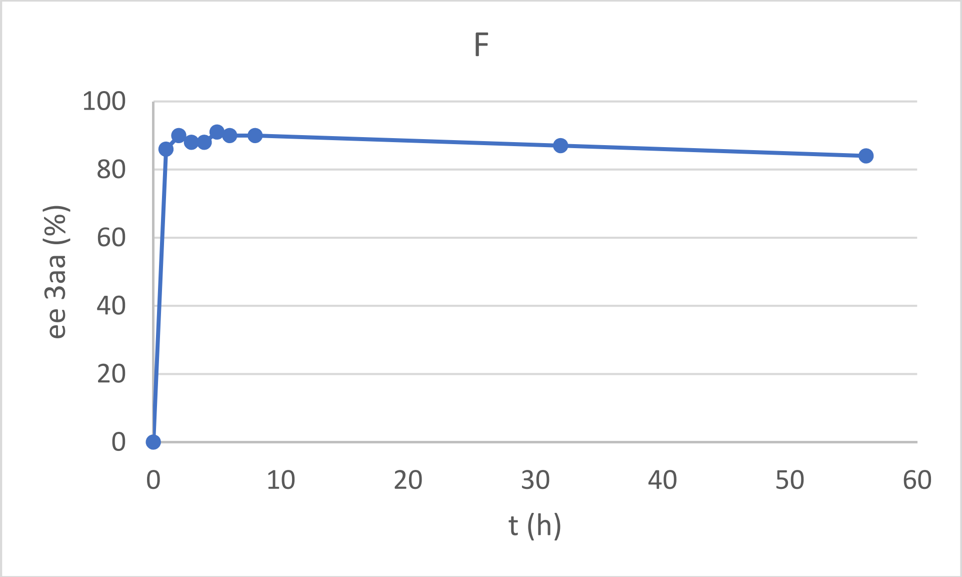

11

7. NMR Data

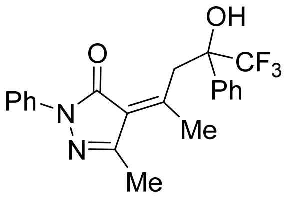

3aa

^{1}H NMR (300 MHz, $CDCl_3$)

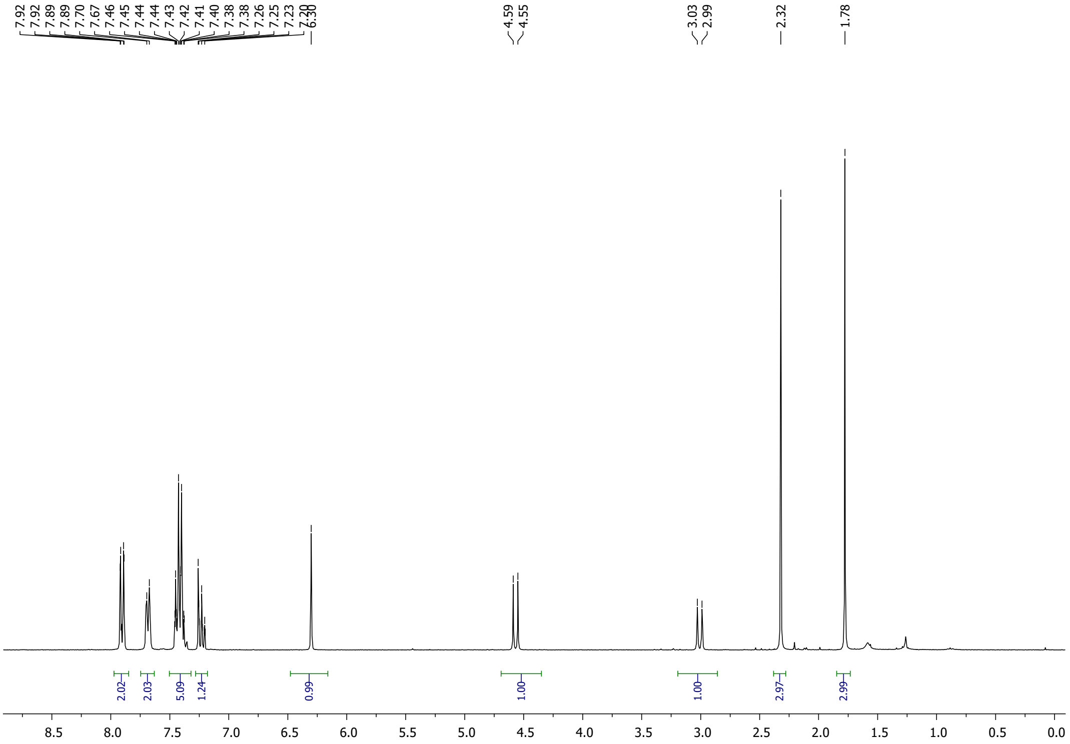

^{13}C {^{1}H} NMR (126 MHz, $CDCl_3$)

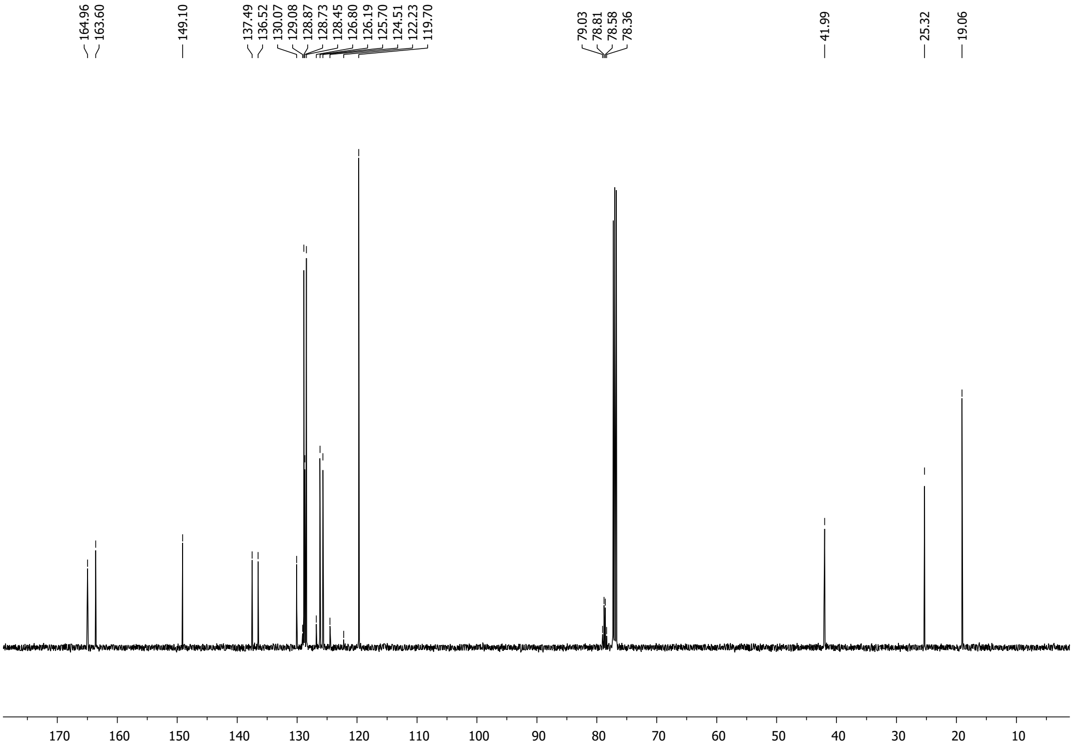

^{19}F NMR (471 MHz, $CDCl_3$)

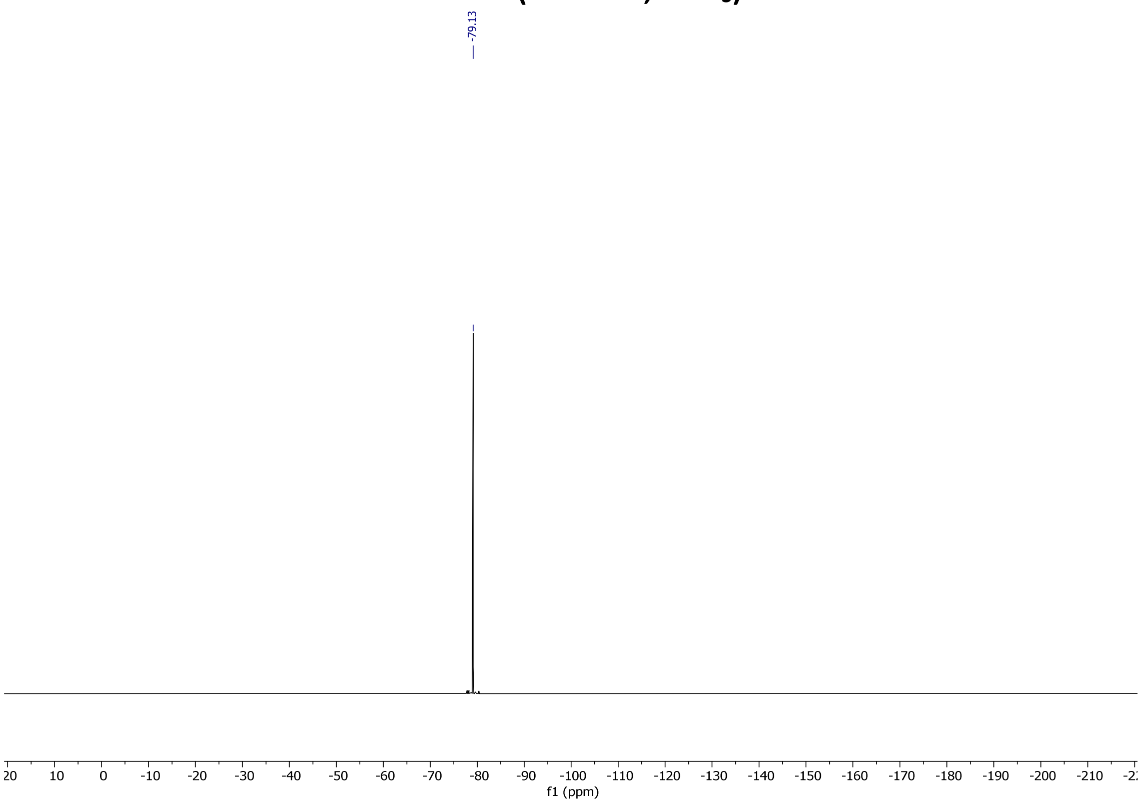

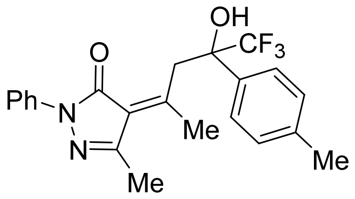

3ab

^{1}H NMR (300 MHz, CDCl$_3$)

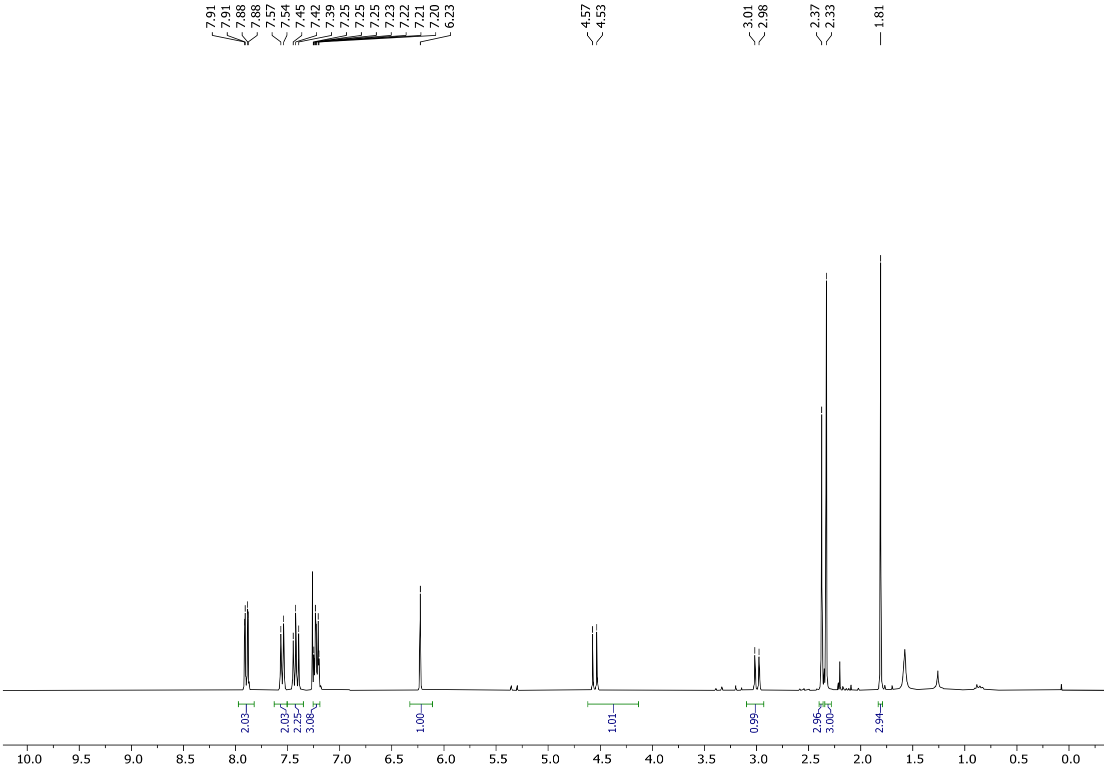

^{13}C {^{1}H} NMR (101 MHz, CDCl$_3$)

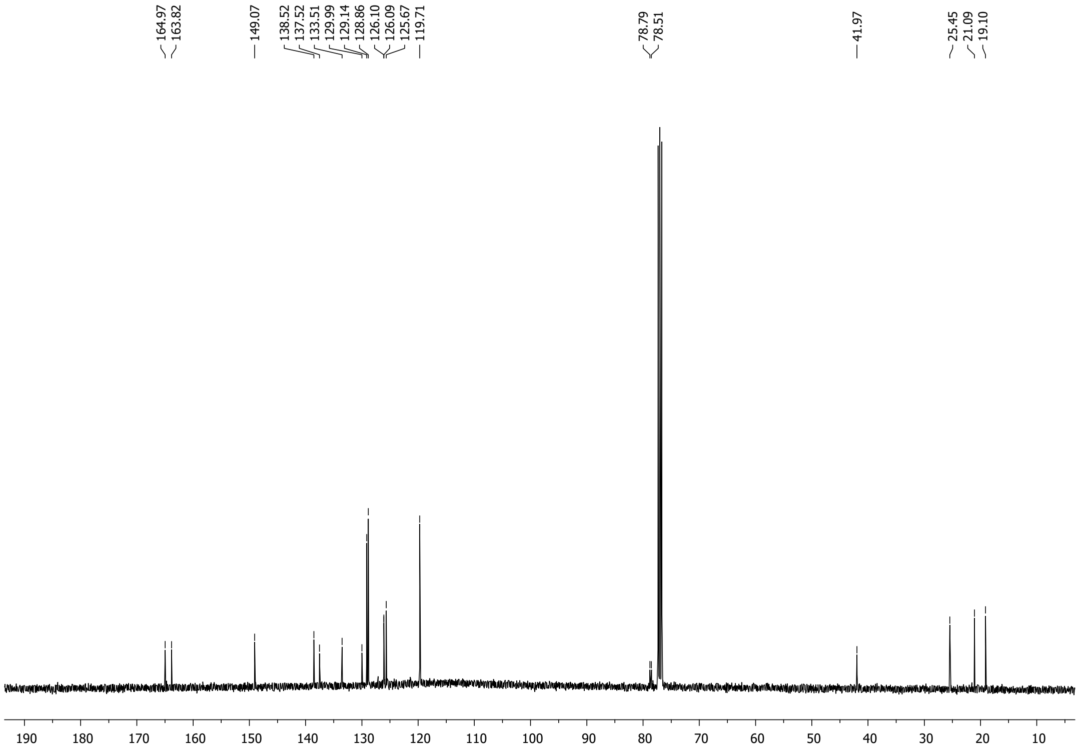

^{19}F NMR (282 MHz, $CDCl_3$)

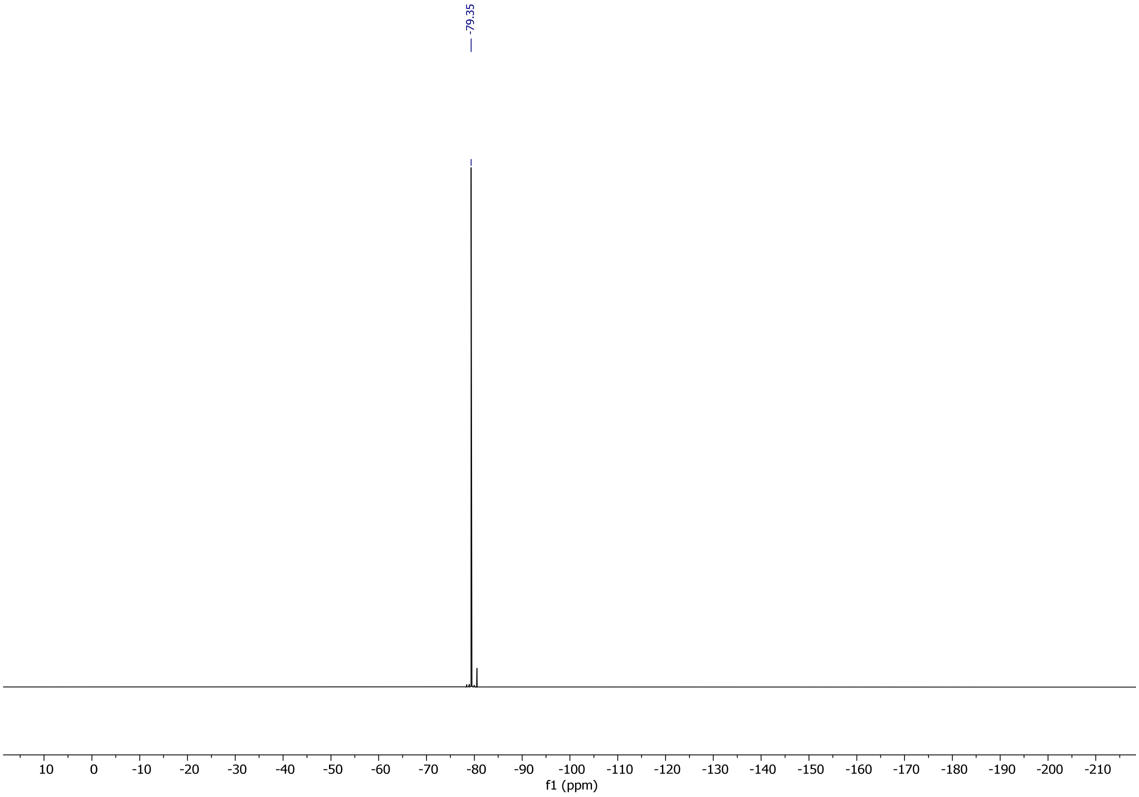

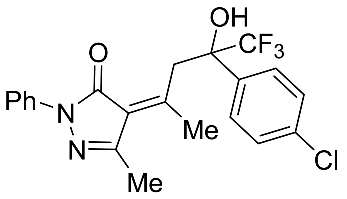

3ac

^{1}H NMR (300 MHz, $CDCl_3$)

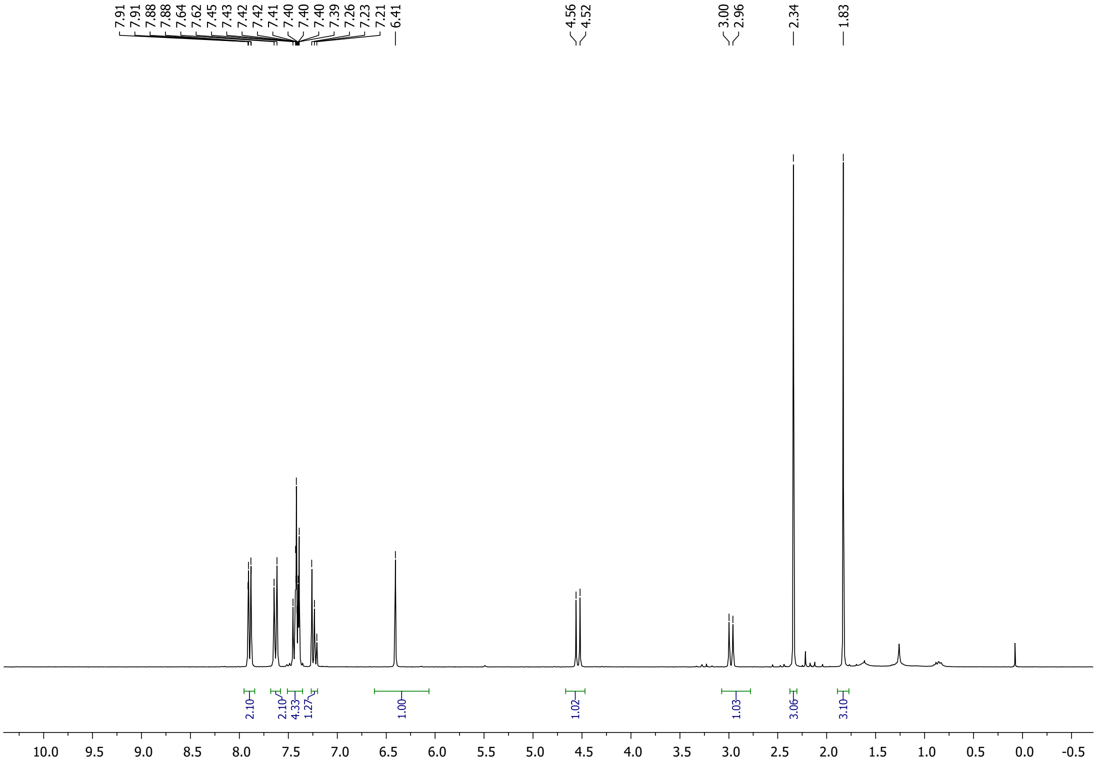

^{13}C {^{1}H} NMR (75 MHz, $CDCl_3$)

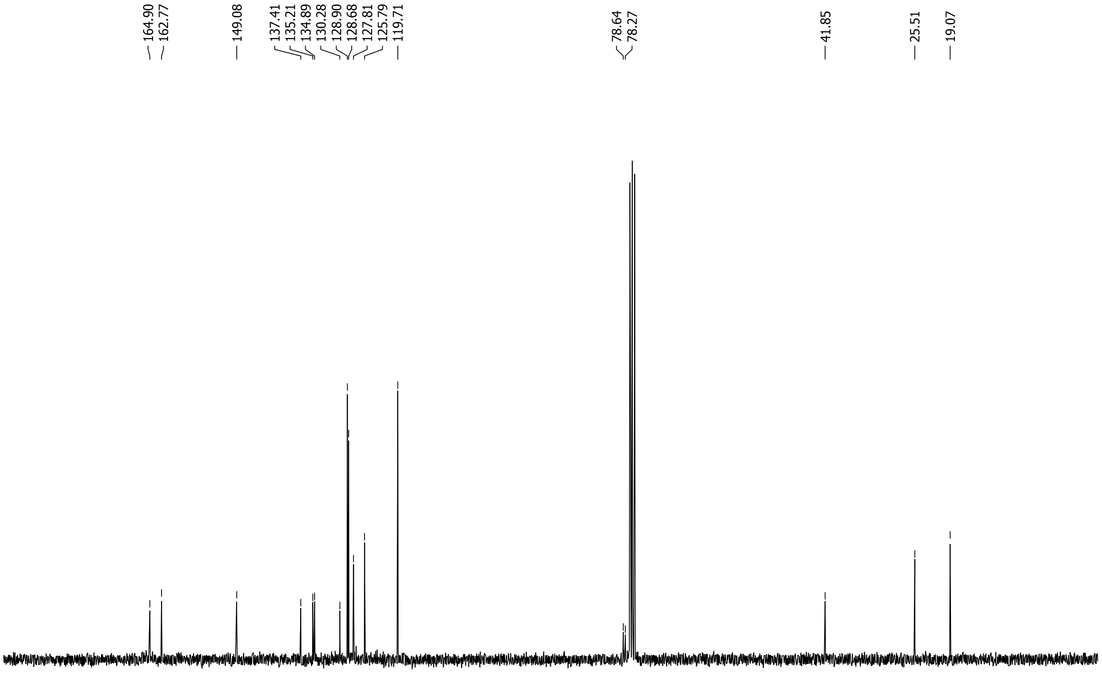

^{19}F NMR (282 MHz, $CDCl_3$)

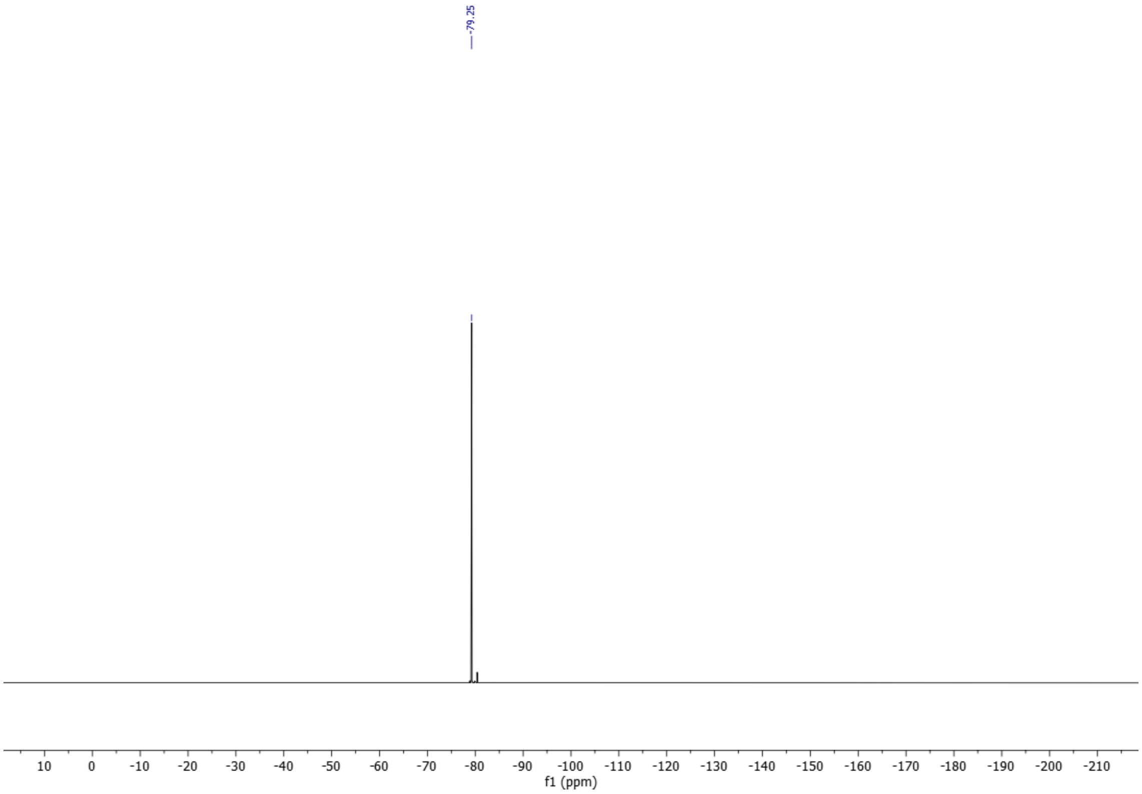

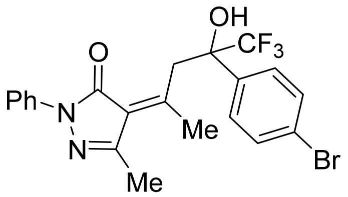

3ad

^{1}H NMR (300 MHz, CDCl$_3$)

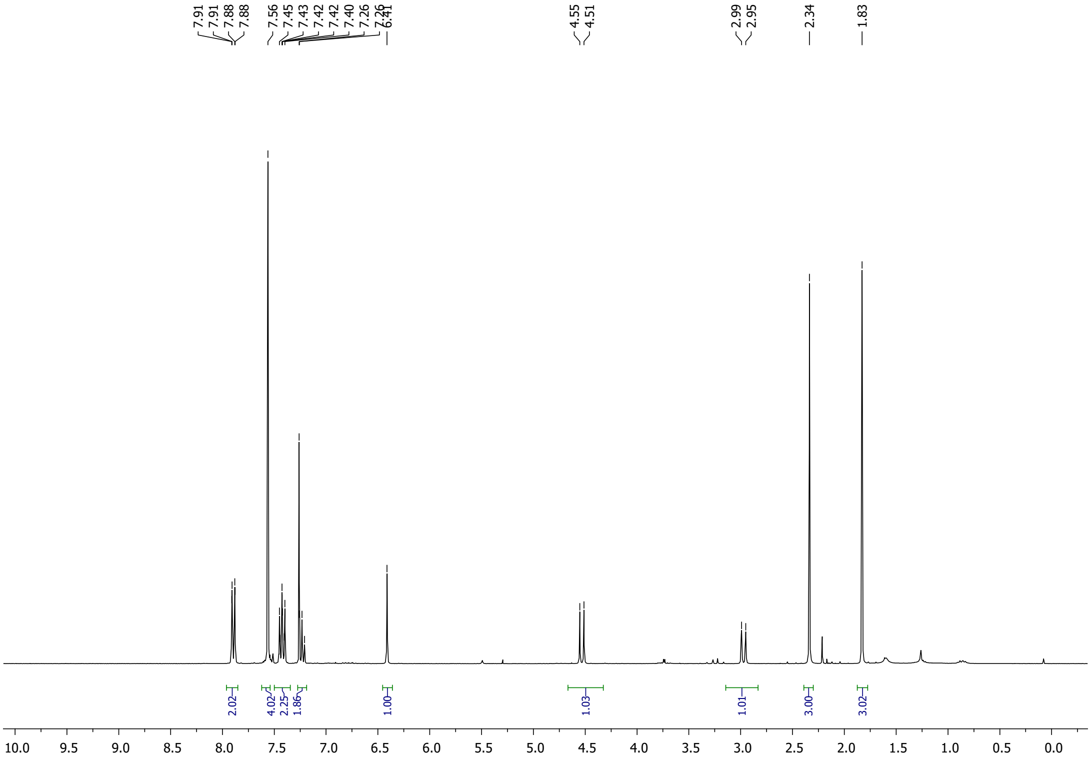

^{13}C {^{1}H} NMR (75 MHz, CDCl$_3$)

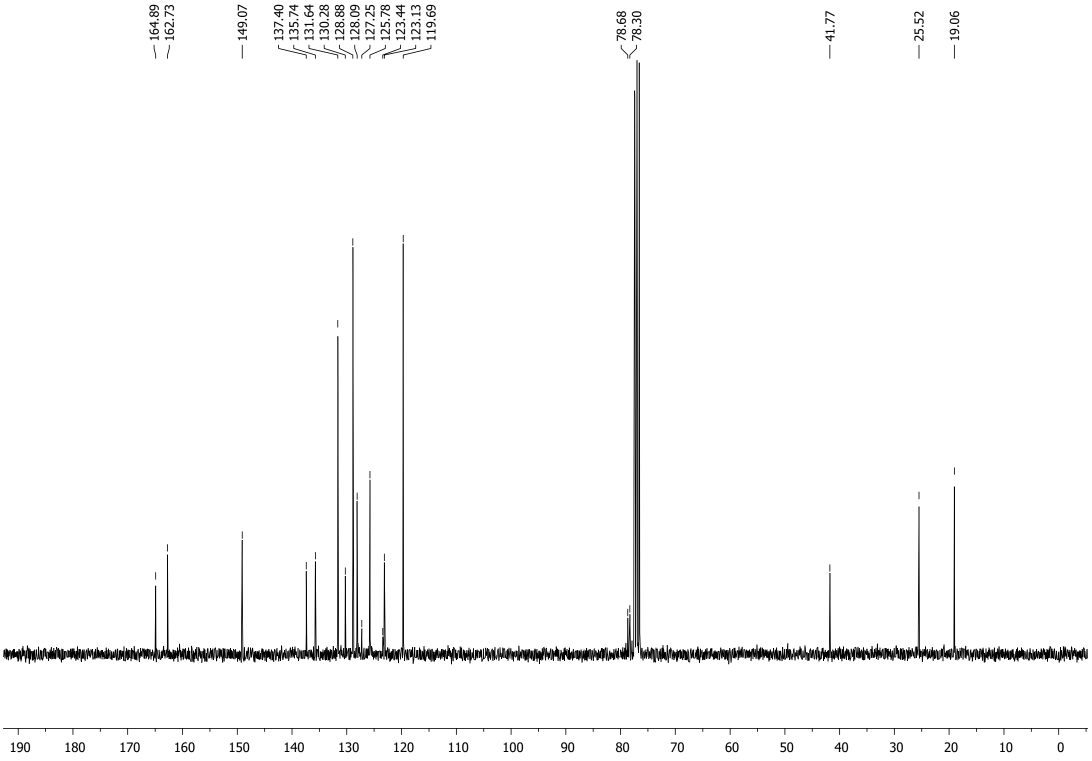

^{19}F NMR (282 MHz, $CDCl_3$)

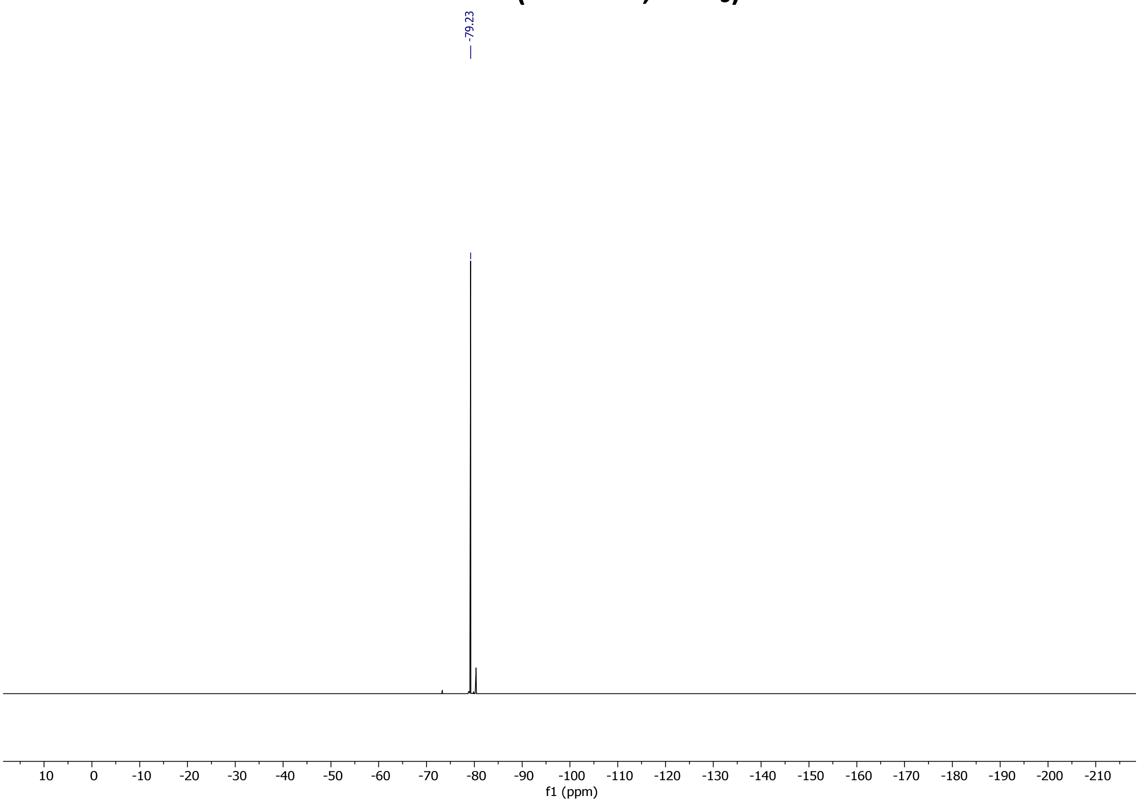

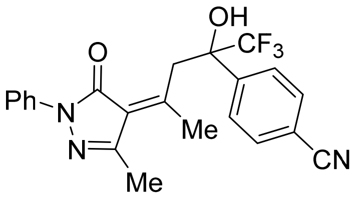

3ae

^{1}H NMR (300 MHz, CDCl$_3$)

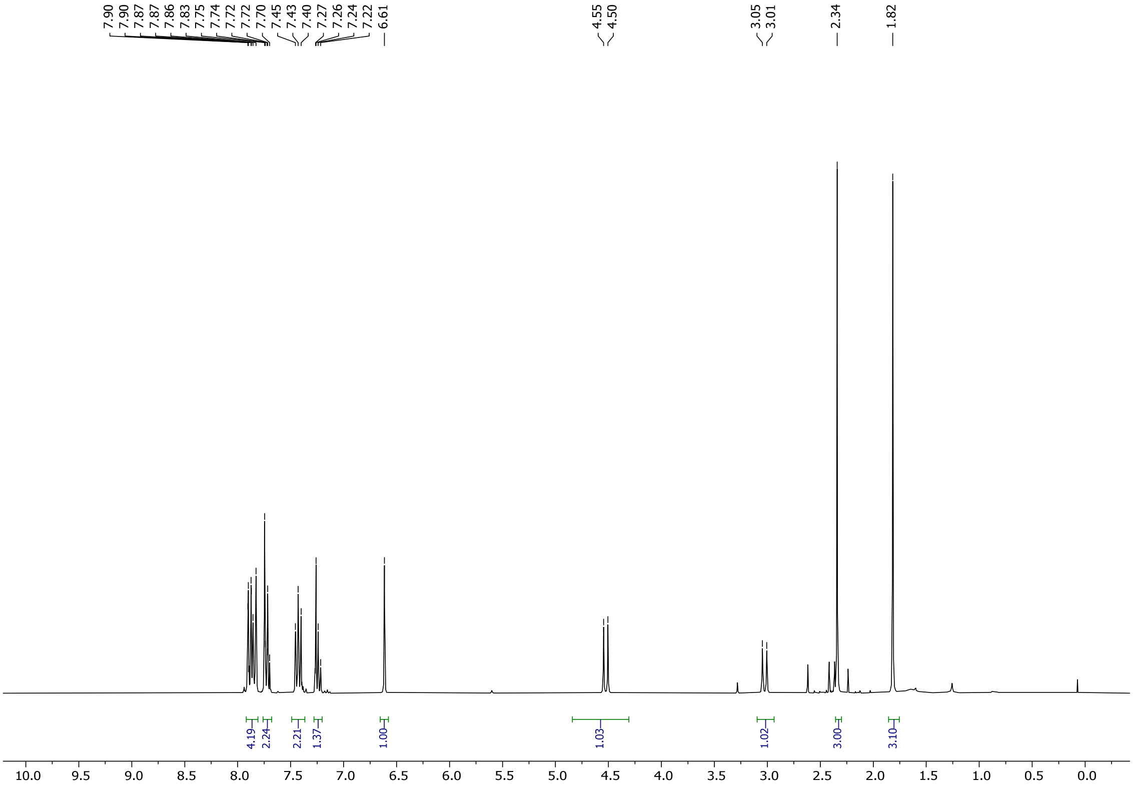

^{13}C {^{1}H} NMR (75 MHz, CDCl$_3$)

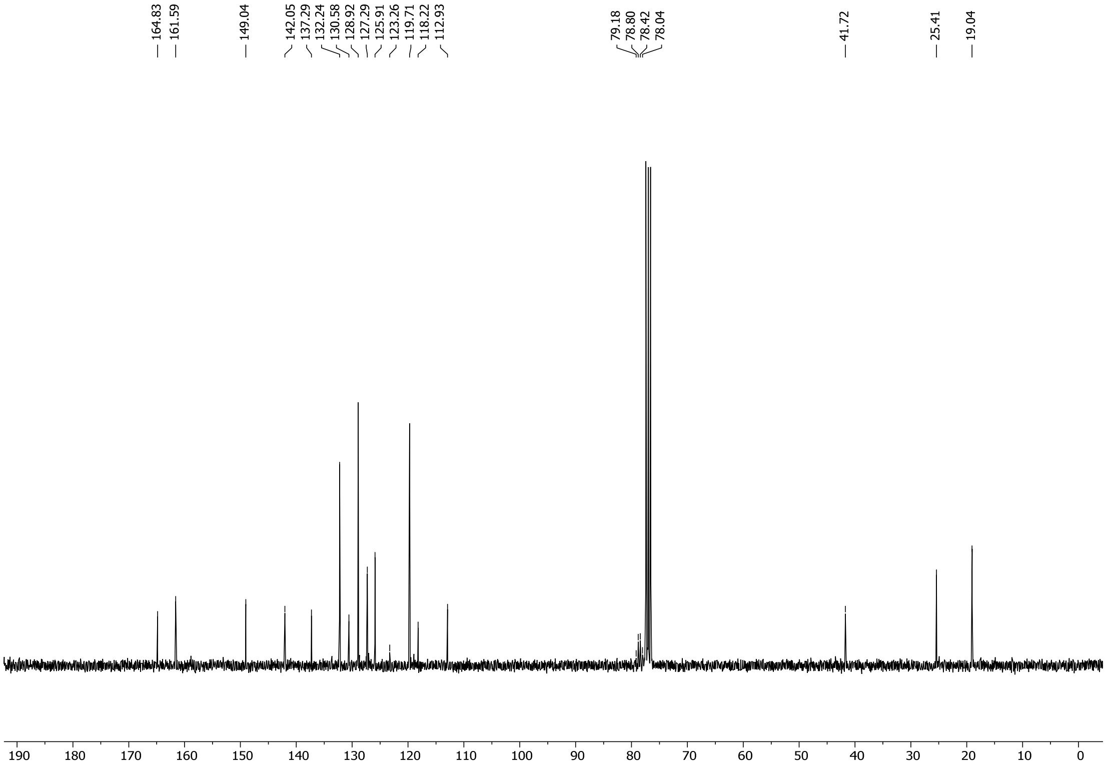

^{19}F NMR (282 MHz, $CDCl_3$)

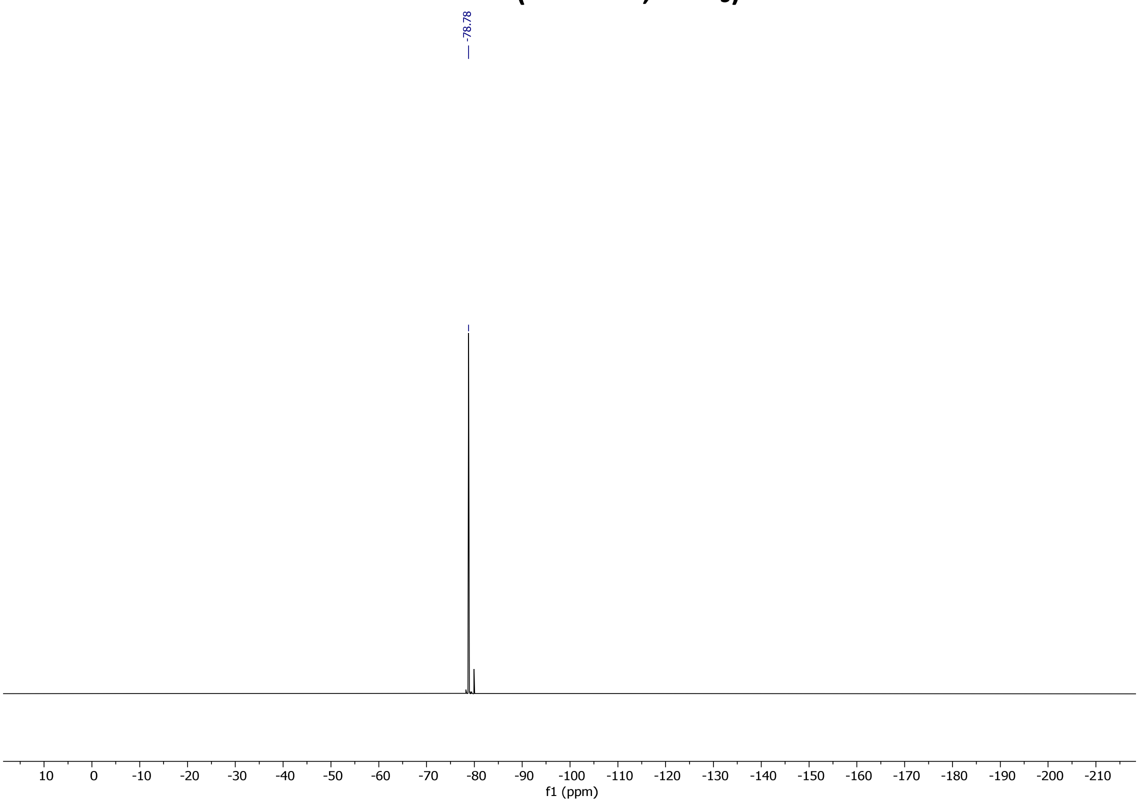

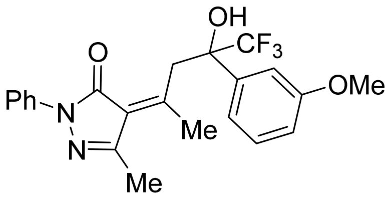
OH
CF3
O
OMe
Ph
N
N
Me
Me

3af

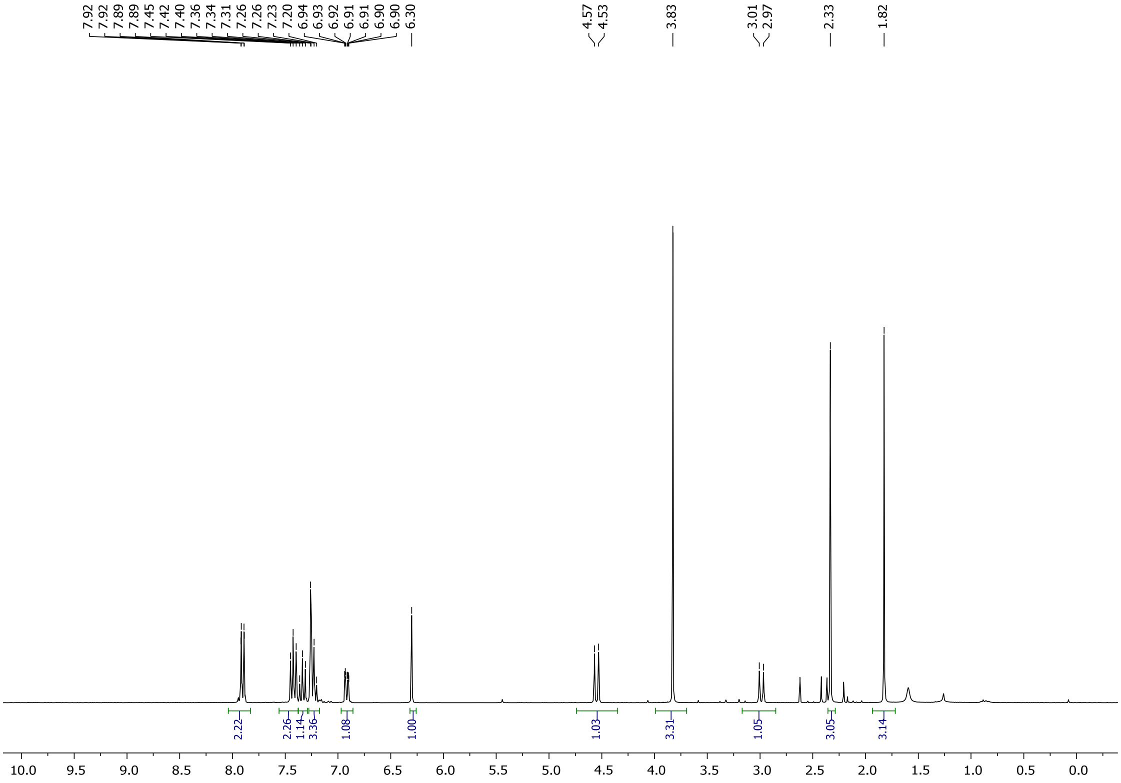
7.92 7.92 7.89 7.89 7.45 7.42 7.40 7.36 7.34 7.31 7.26 7.26 7.23 7.20 6.94 6.93 6.92 6.91 6.91 6.90 6.90 6.30
4.57 4.53
3.83
3.01 2.97
2.33
1.82
2.22
2.26
1.14
3.36
1.08
1.00
1.03
3.31
1.05
3.05
3.14
10.0 9.5 9.0 8.5 8.0 7.5 7.0 6.5 6.0 5.5 5.0 4.5 4.0 3.5 3.0 2.5 2.0 1.5 1.0 0.5 0.0

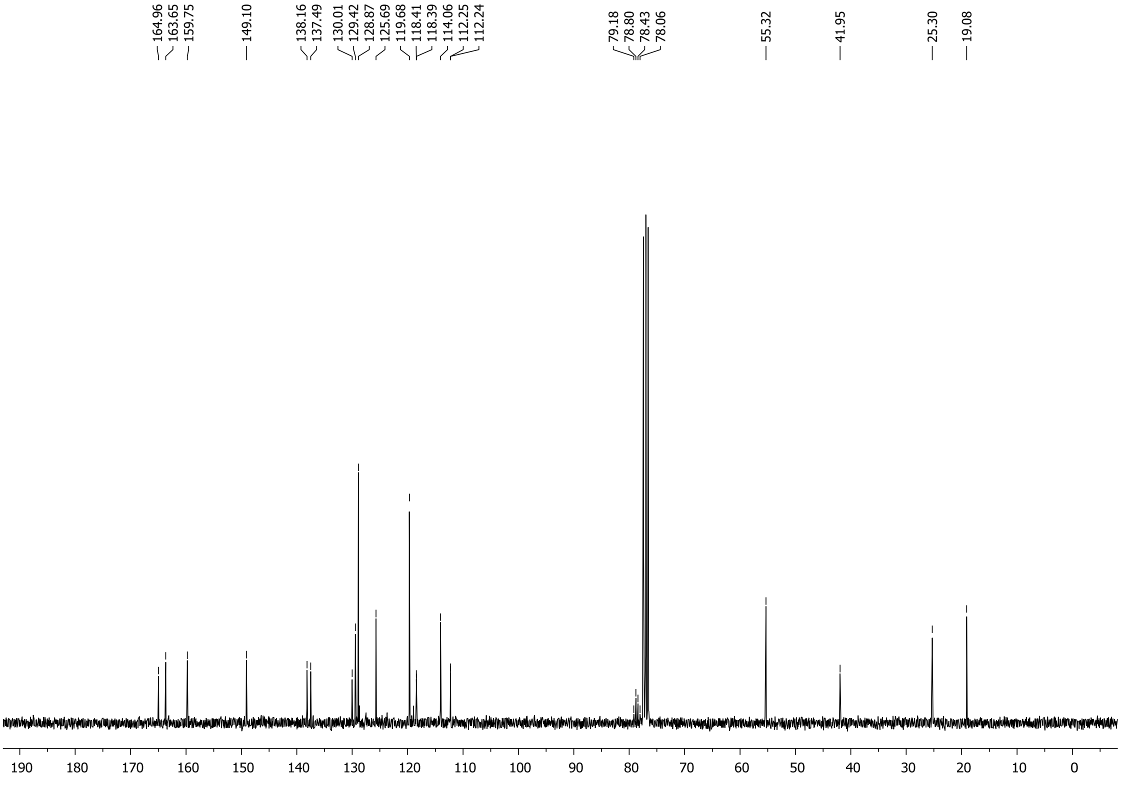
164.96 163.65 159.75
149.10
138.16 137.49
130.01 129.42 128.87 125.69 119.68 118.41 118.39 114.06 112.25 112.24
79.18 78.80 78.43 78.06
55.32
41.95
25.30
19.08
190 180 170 160 150 140 130 120 110 100 90 80 70 60 50 40 30 20 10 0

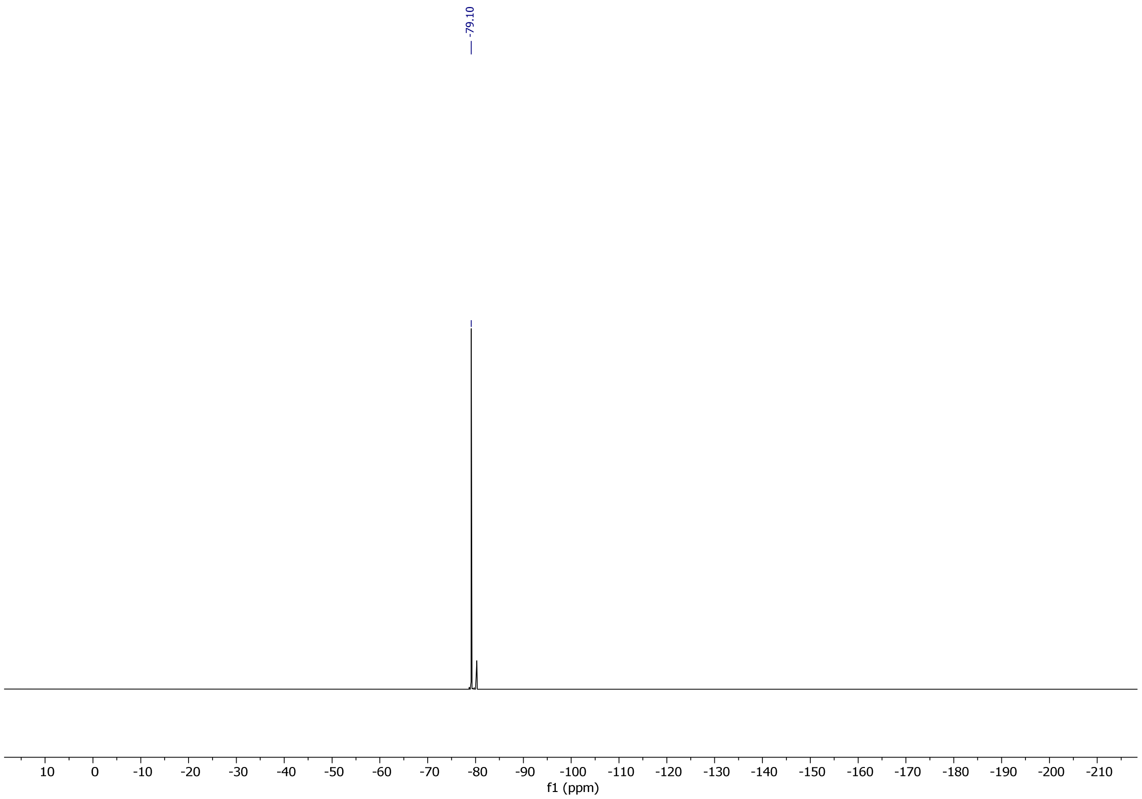
-79.10
10 0 -10 -20 -30 -40 -50 -60 -70 -80 -90 -100 -110 -120 -130 -140 -150 -160 -170 -180 -190 -200 -210
f1 (ppm)

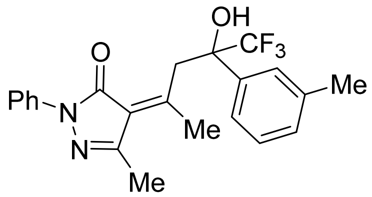

3ag

^{1}H NMR (300 MHz, CDCl$_3$)

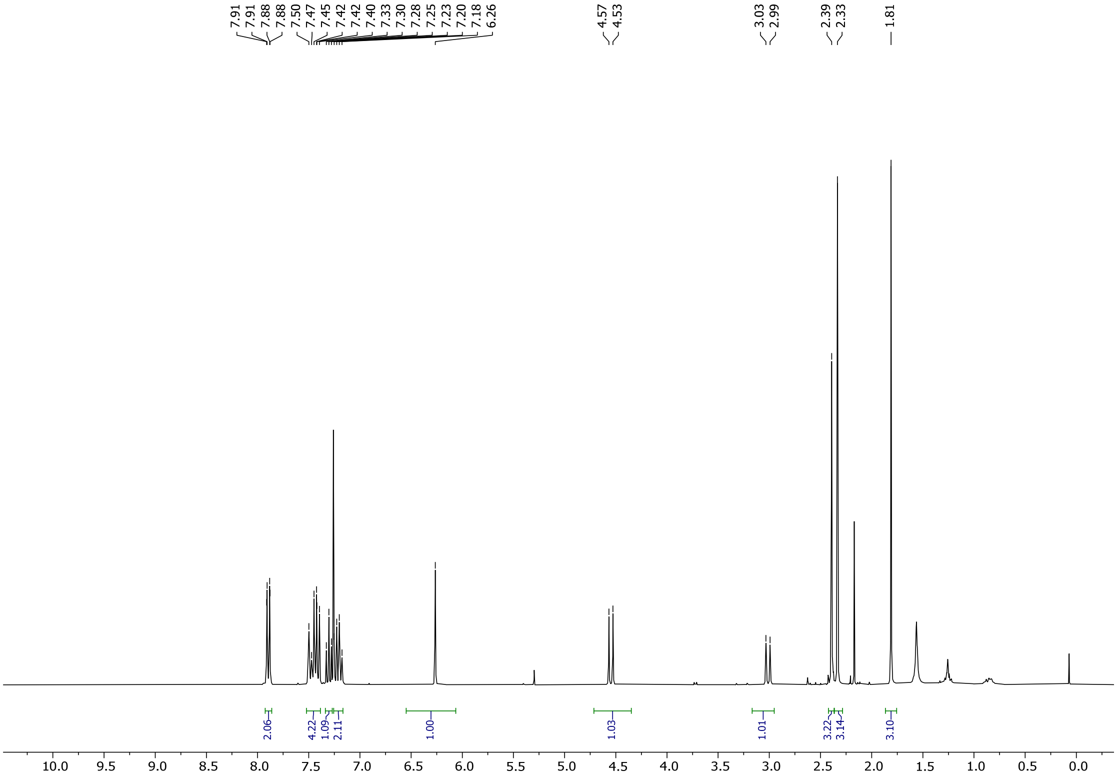

^{13}C {^{1}H} NMR (101 MHz, CDCl$_3$)

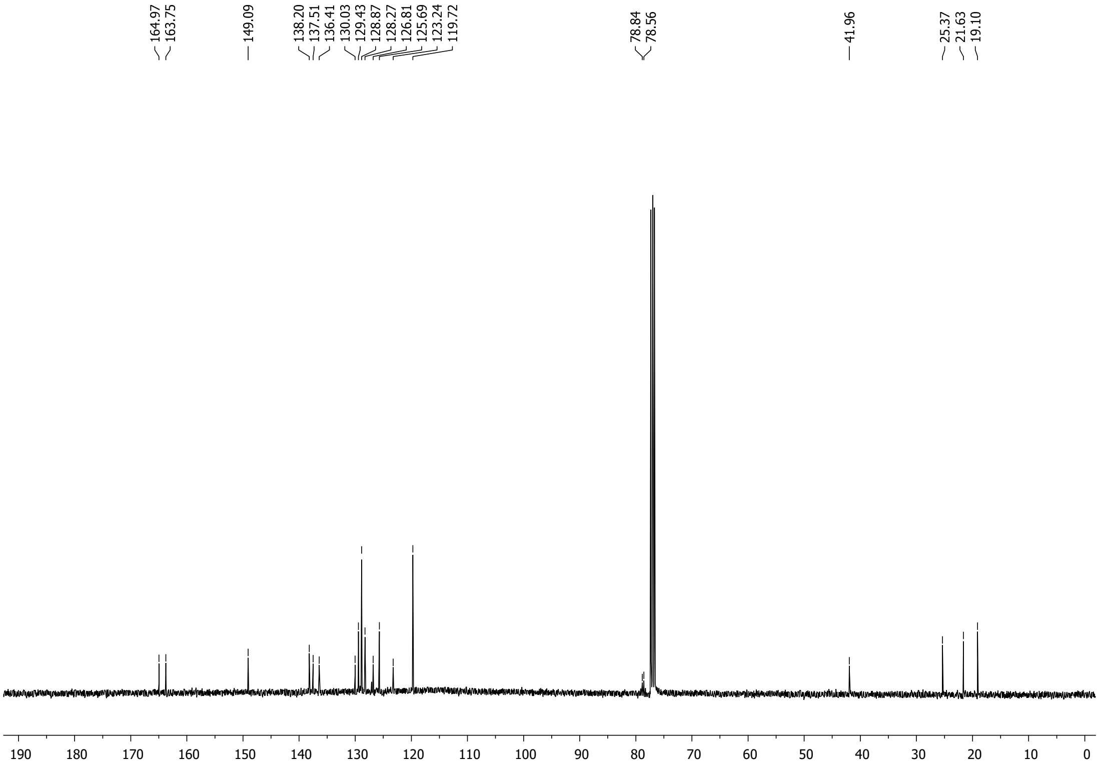

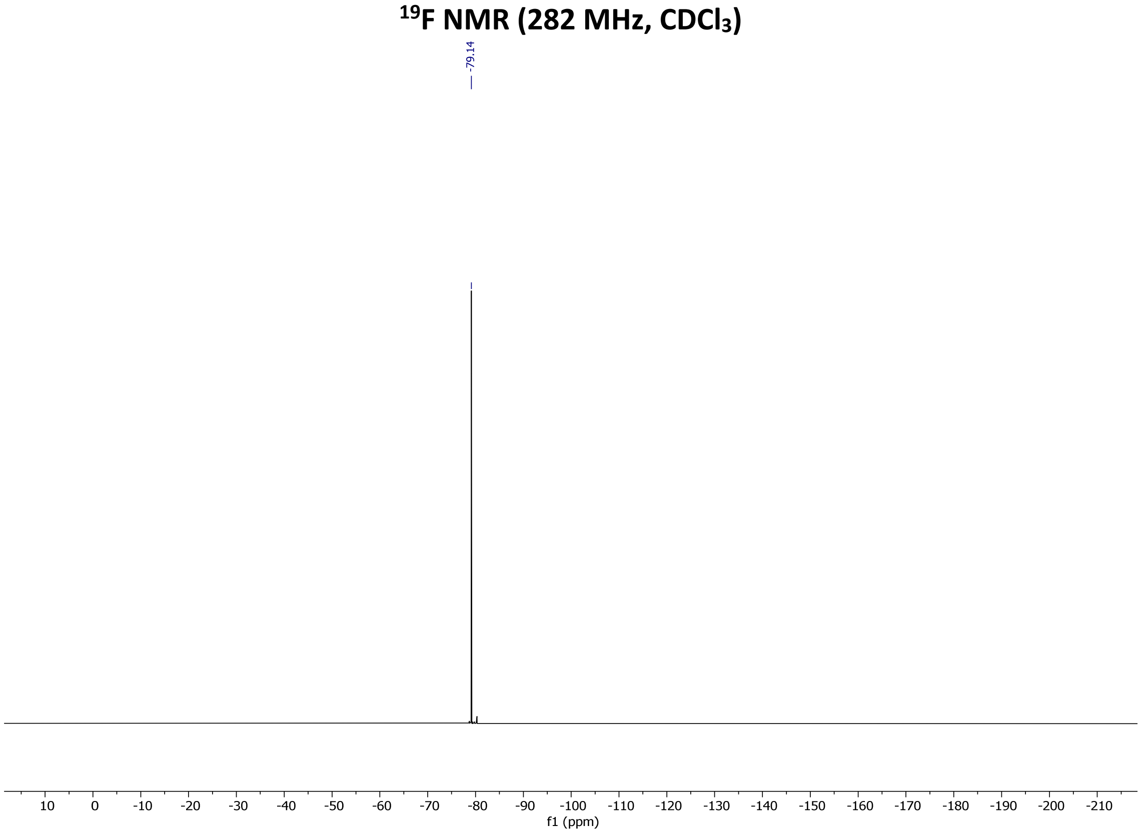
^{19}F NMR (282 MHz, $CDCl_3$)
-79.14
10 0 -10 -20 -30 -40 -50 -60 -70 -80 -90 -100 -110 -120 -130 -140 -150 -160 -170 -180 -190 -200 -210
f1 (ppm)

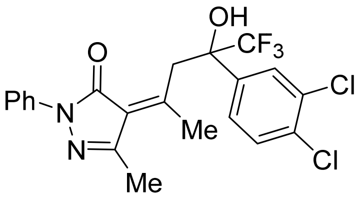

3ah

^{1}H NMR (300 MHz, CDCl$_3$)

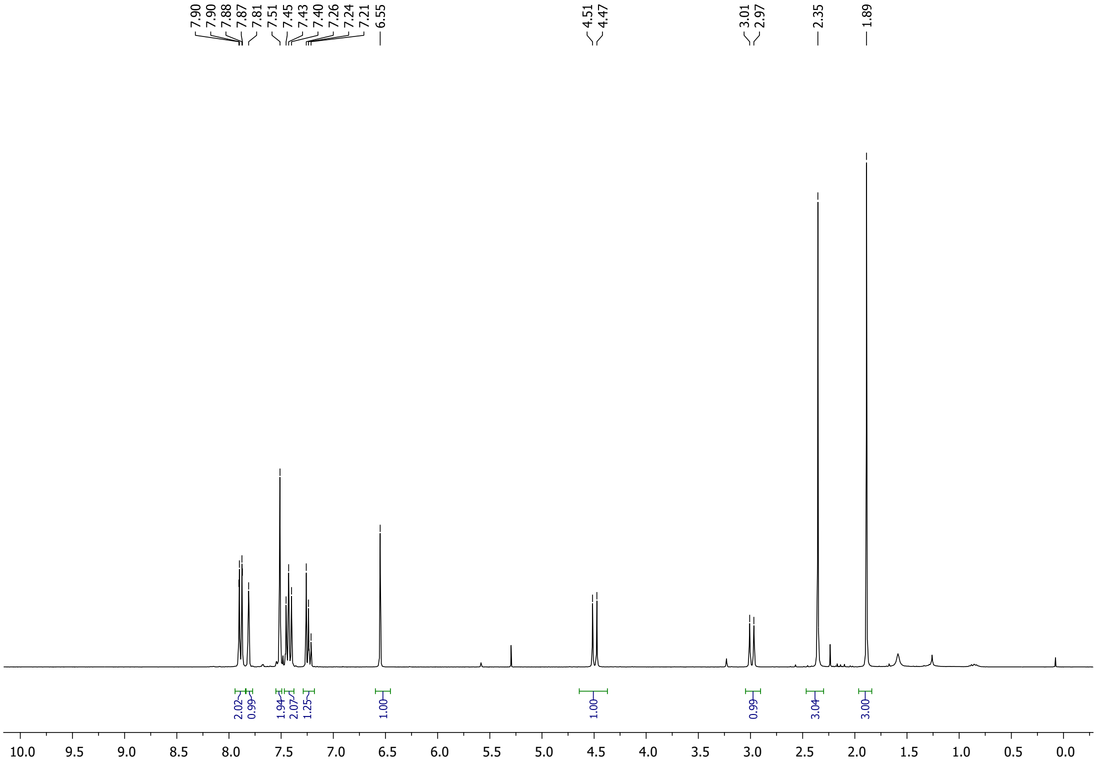

^{13}C {^{1}H} NMR (75 MHz, CDCl$_3$)

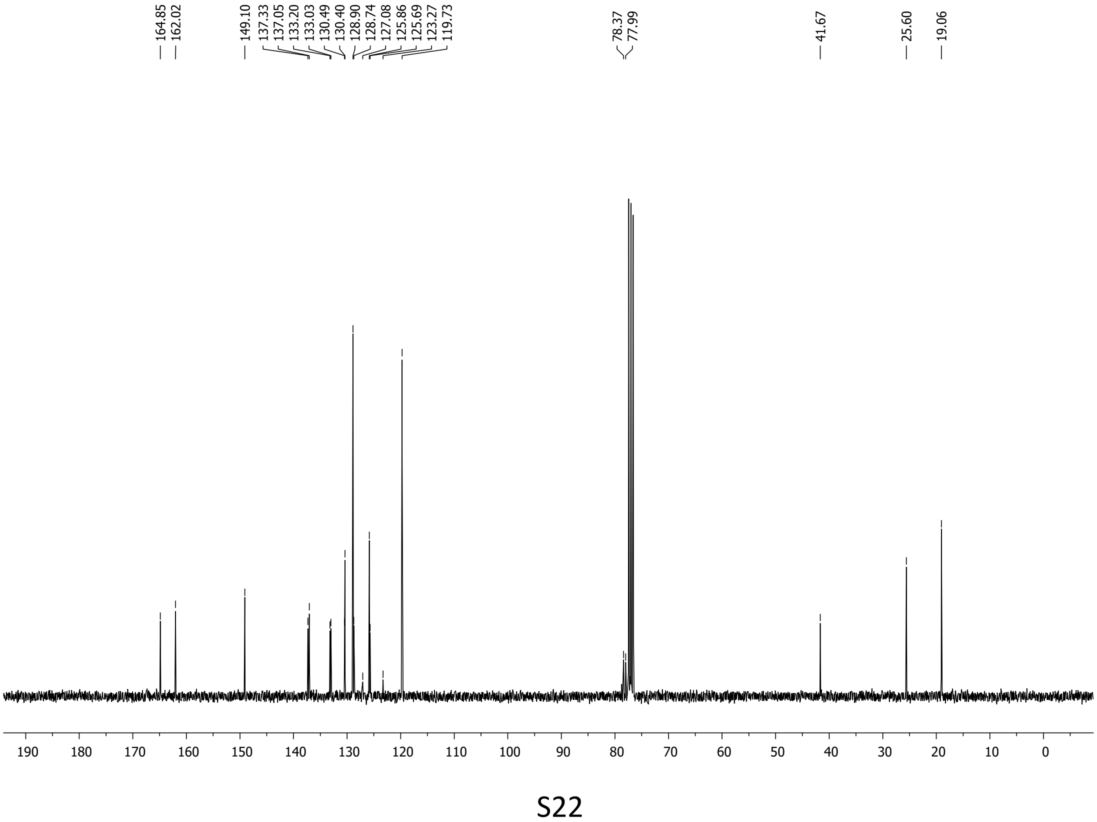

^{19}F NMR (282 MHz, $CDCl_3$)

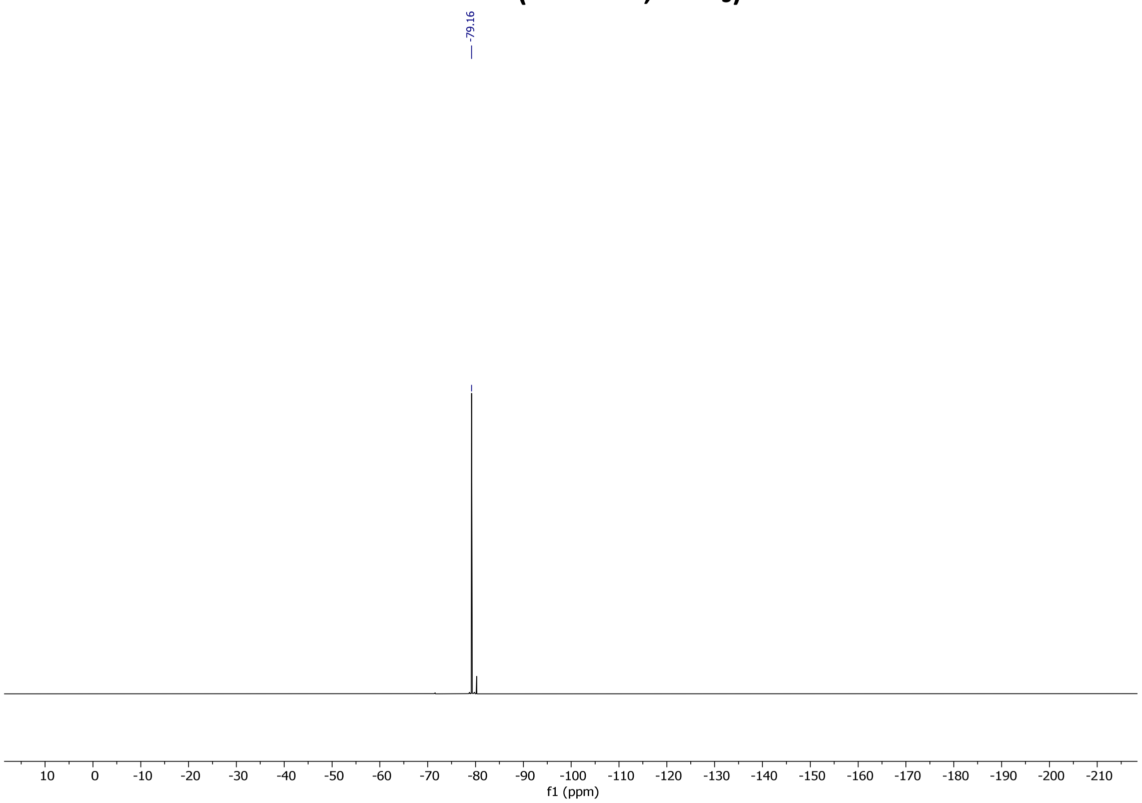

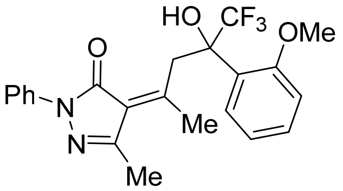

3ai

^{1}H NMR (300 MHz, $CDCl_3$)

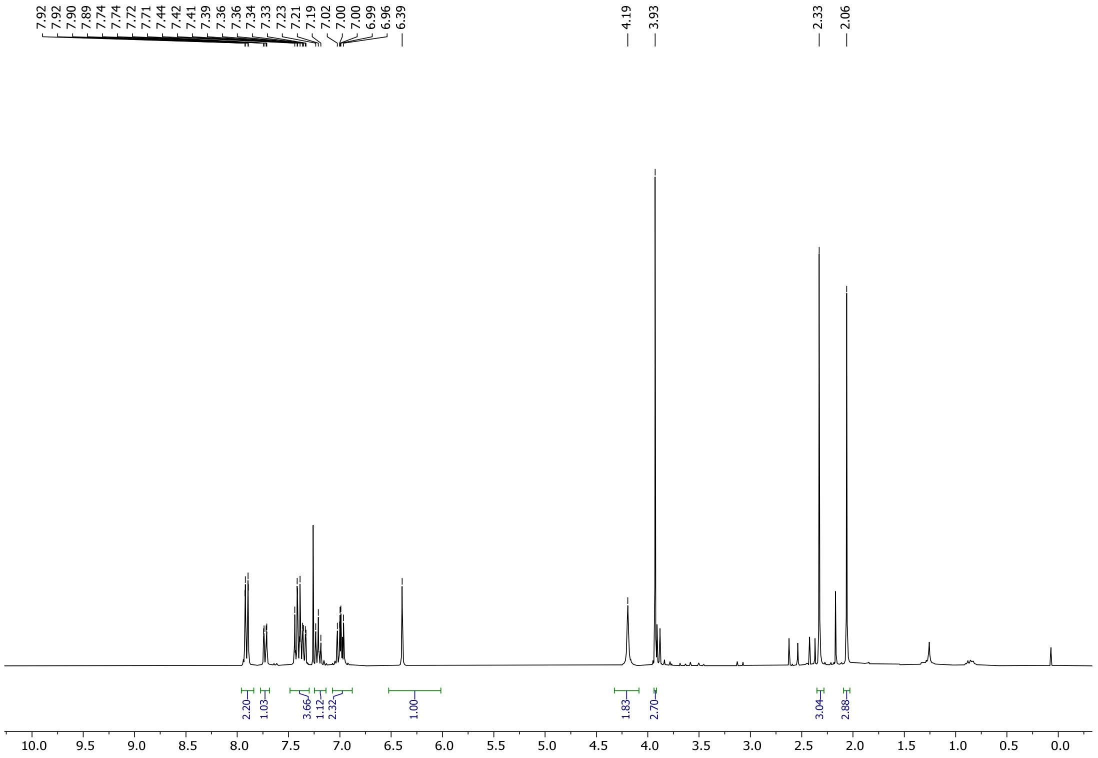

^{13}C {^{1}H} NMR (101 MHz, $CDCl_3$)

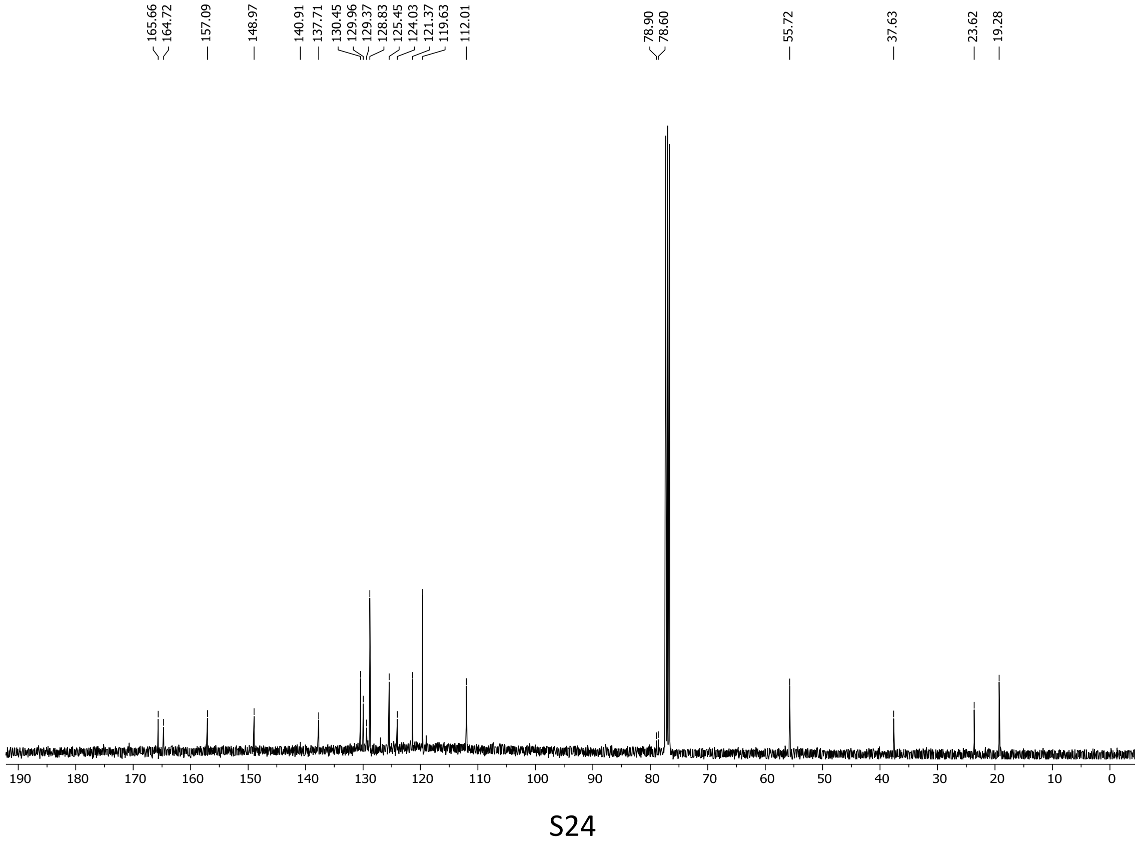

^{19}F NMR (282 MHz, $CDCl_3$)

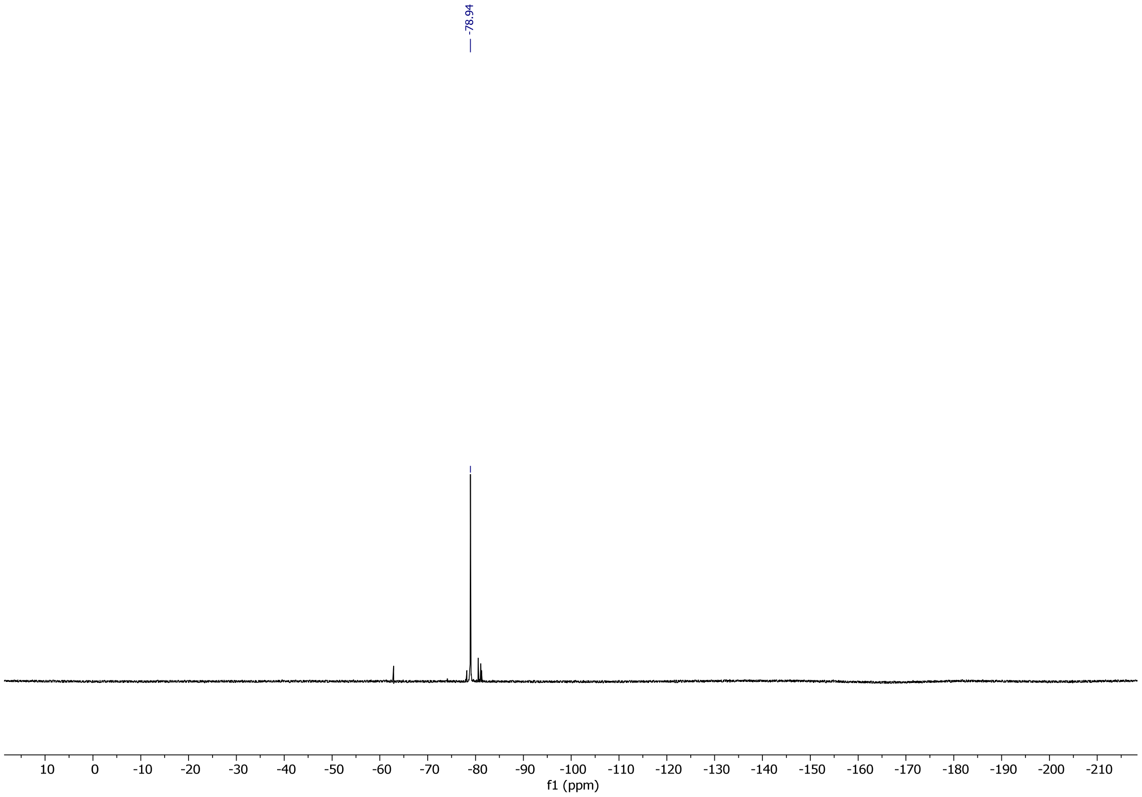

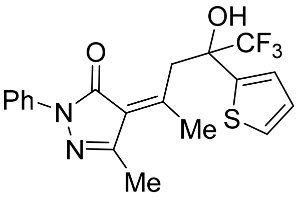

3aj

^{1}H NMR (300 MHz, CDCl$_3$)

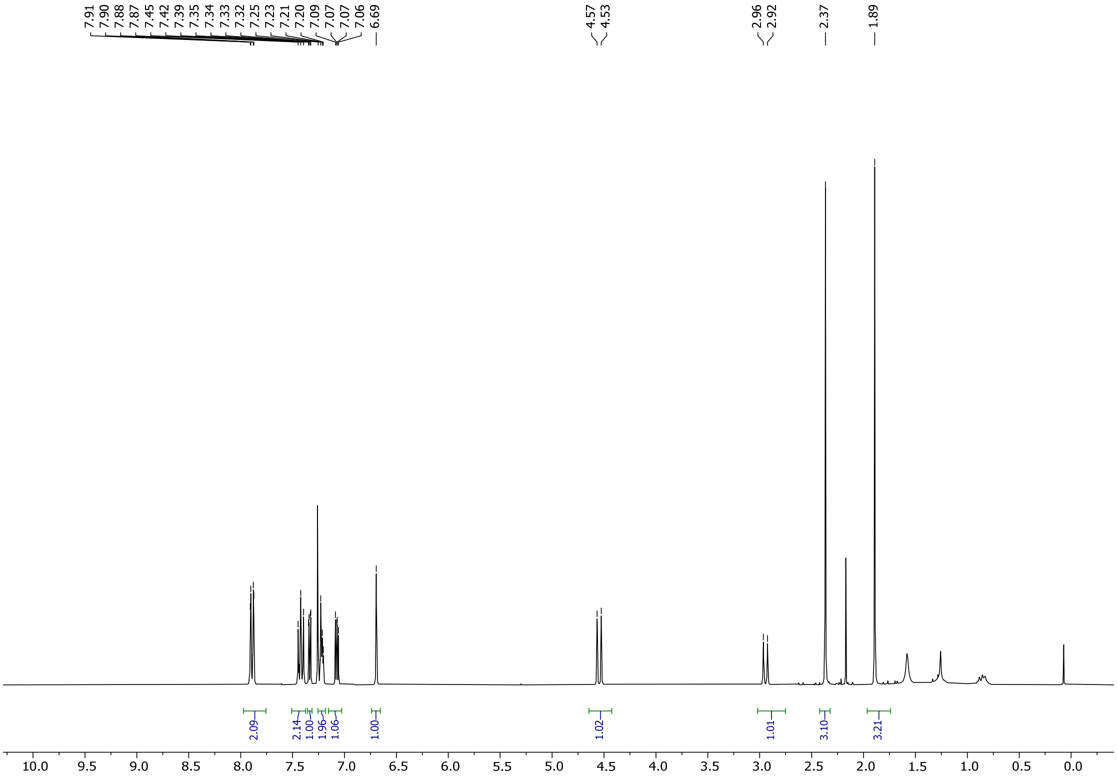

^{13}C {^{1}H} NMR (75 MHz, CDCl$_3$)

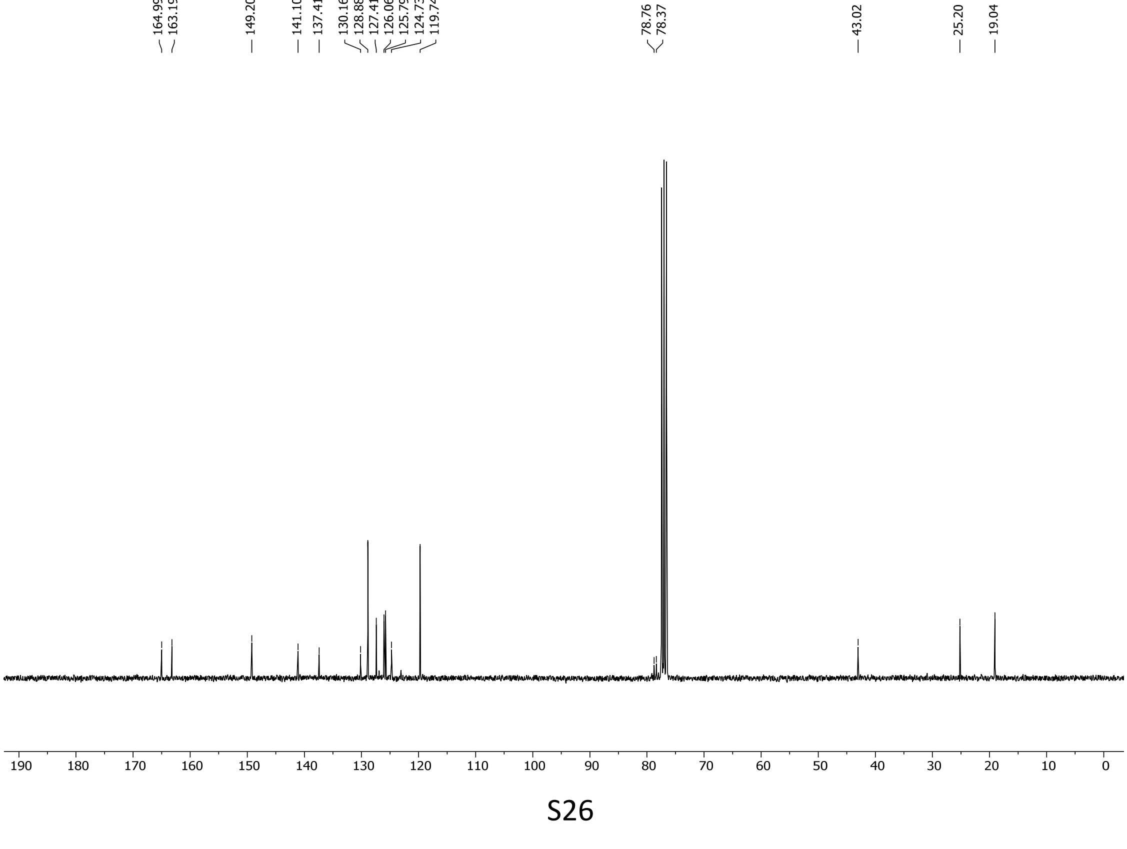

^{19}F NMR (282 MHz, $CDCl_3$)

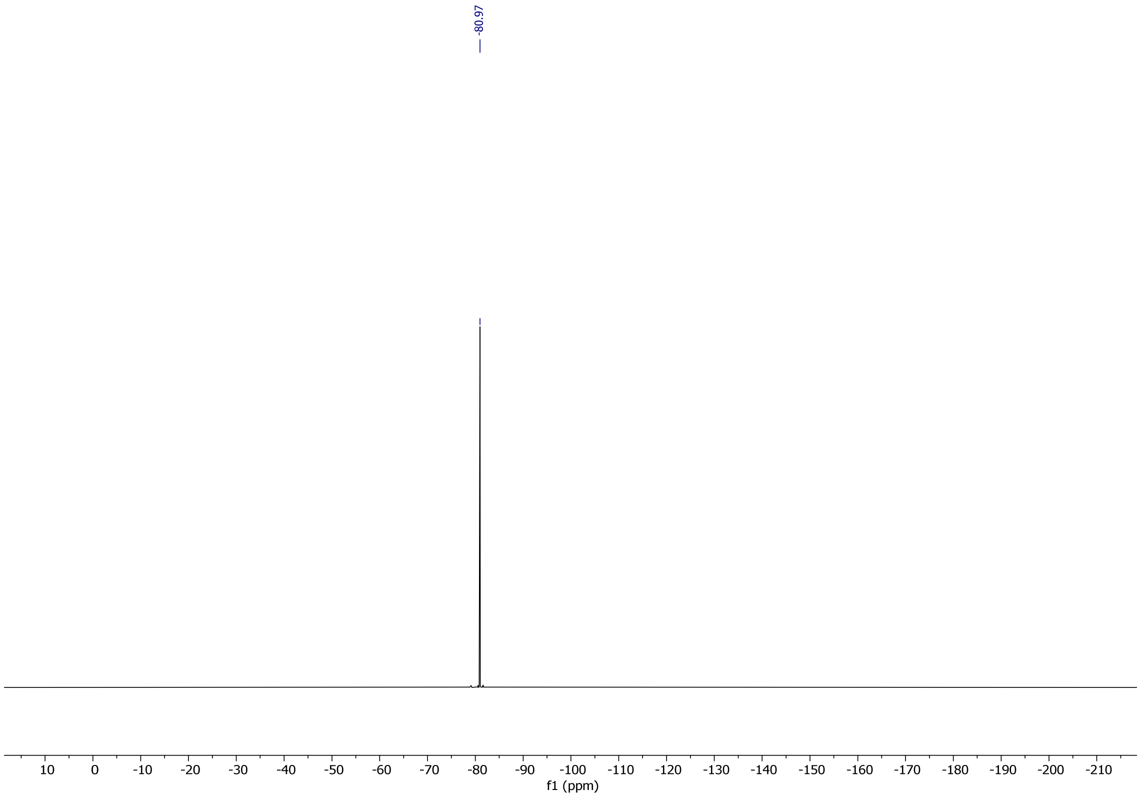

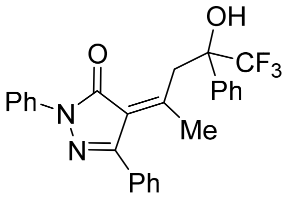

3ba

^{1}H NMR (300 MHz, $CDCl_3$)

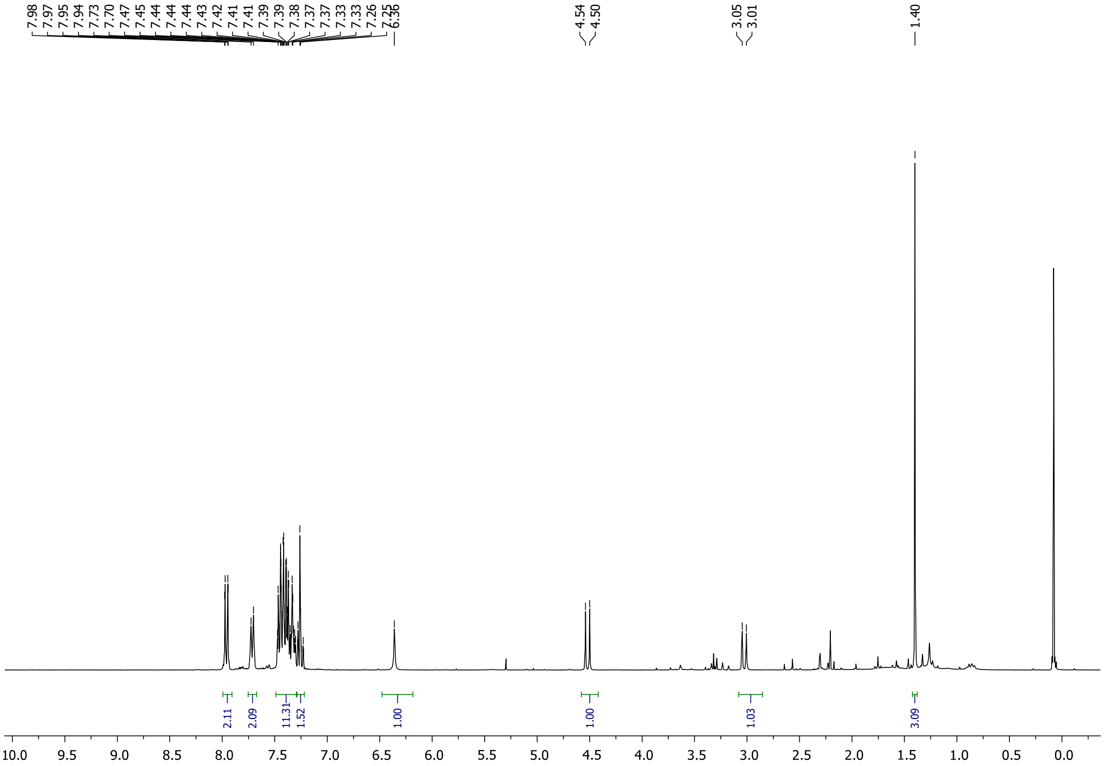

^{13}C {^{1}H} NMR (75 MHz, $CDCl_3$)

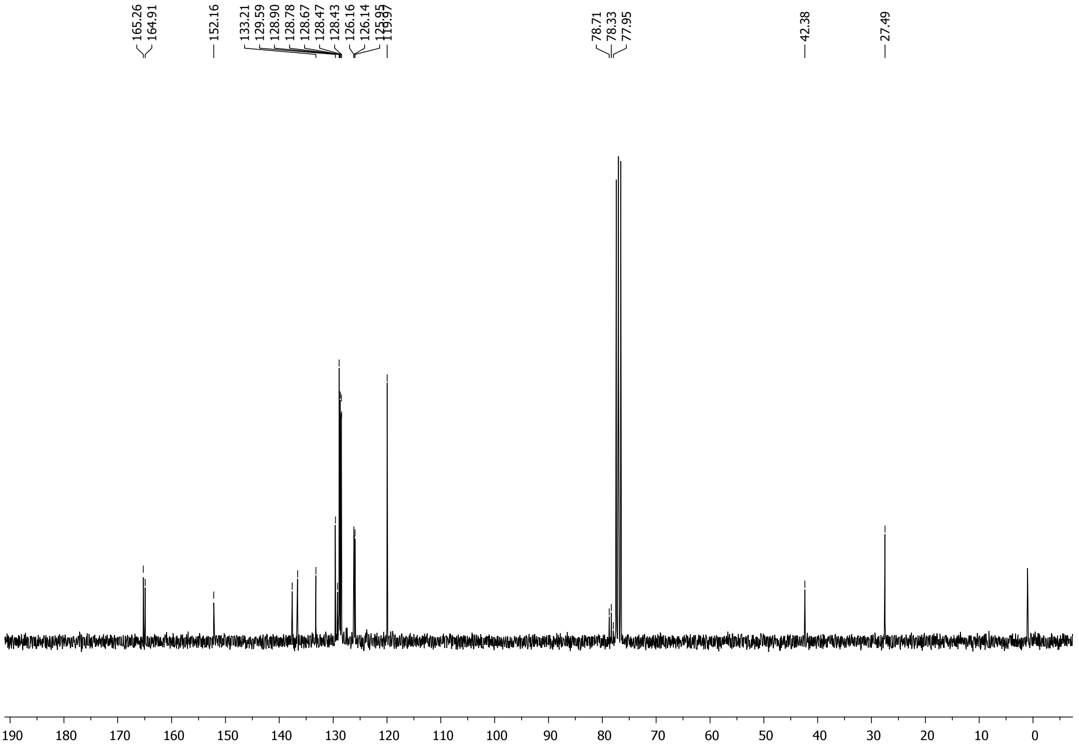

^{19}F NMR (282 MHz, $CDCl_3$)

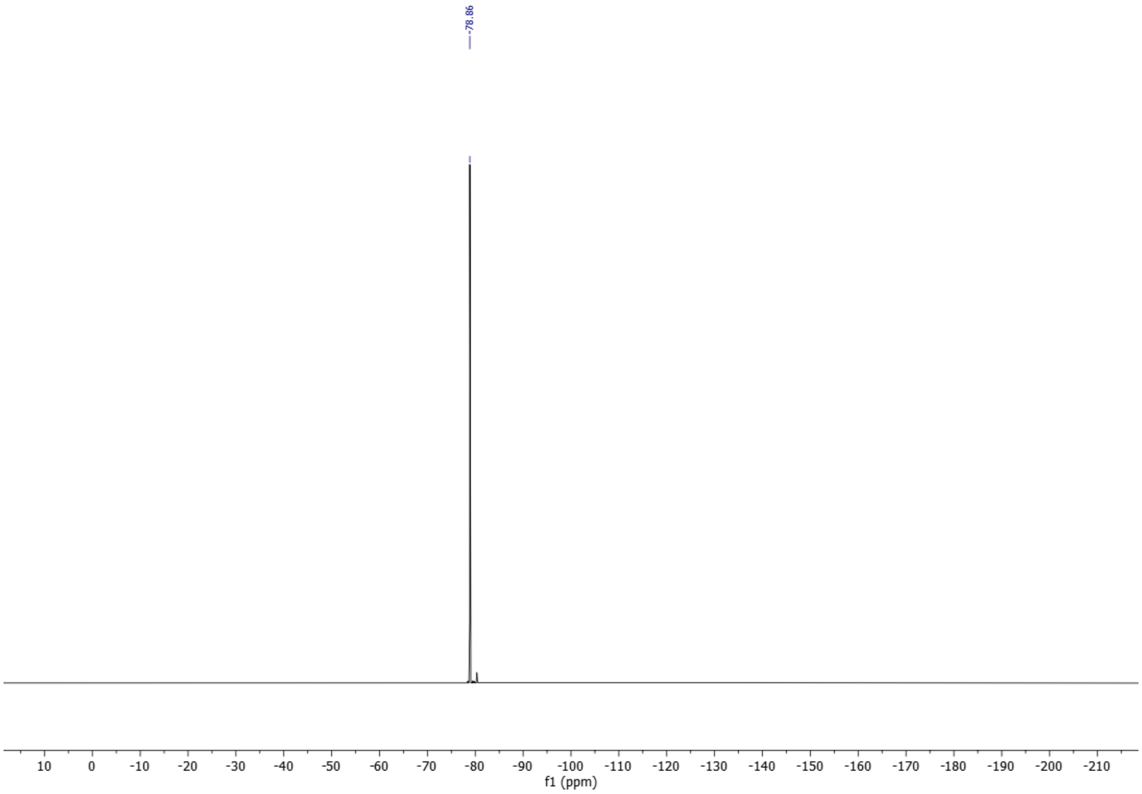

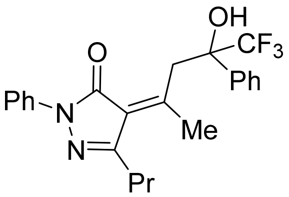

3ca

^{1}H NMR (300 MHz, CDCl$_3$)

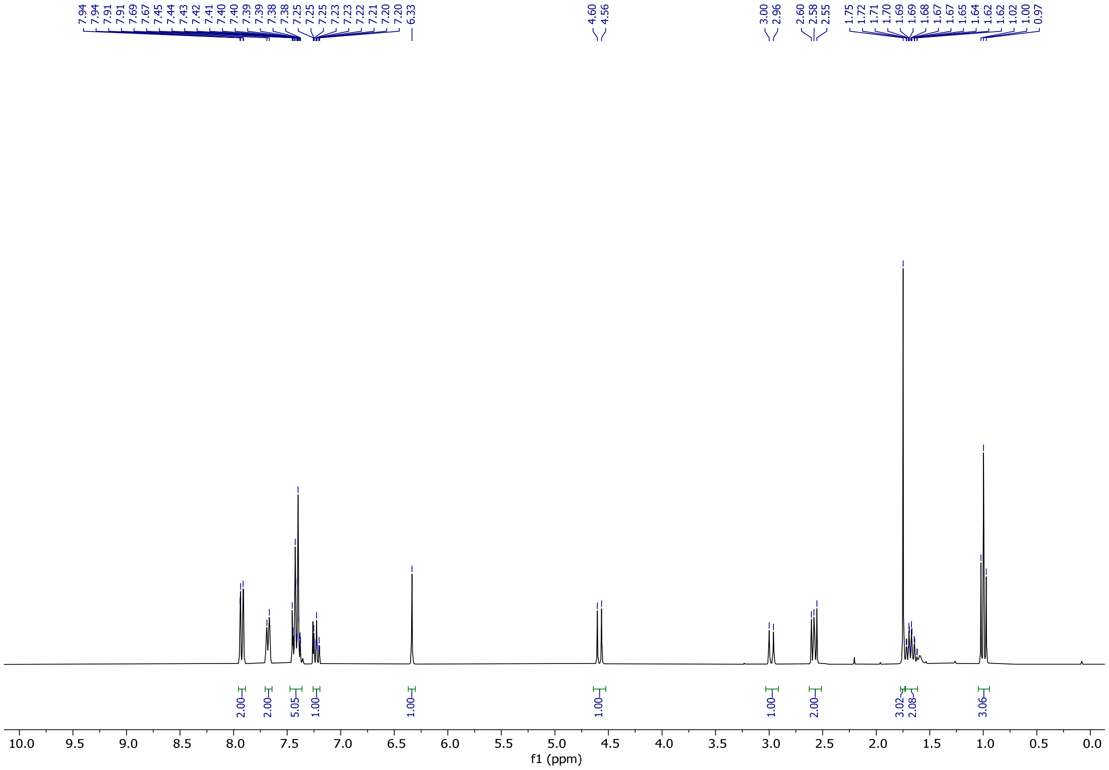

^{13}C {^{1}H} NMR (75 MHz, CDCl$_3$)

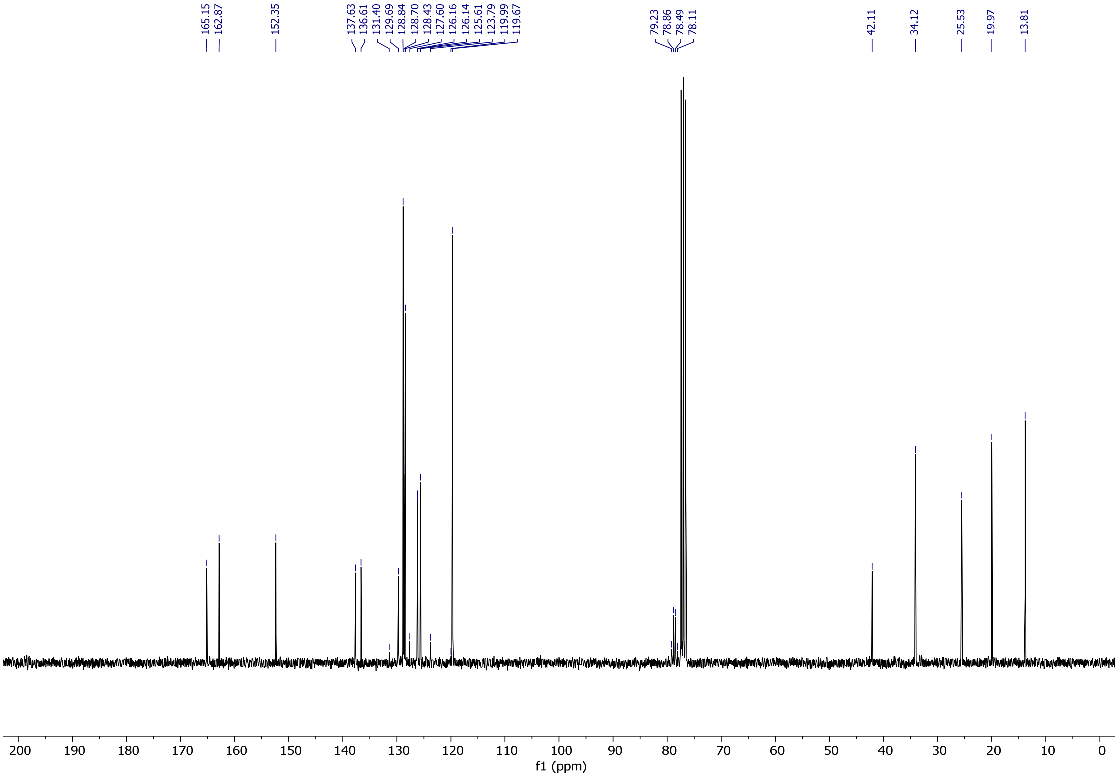

^{19}F NMR (282 MHz, $CDCl_3$)

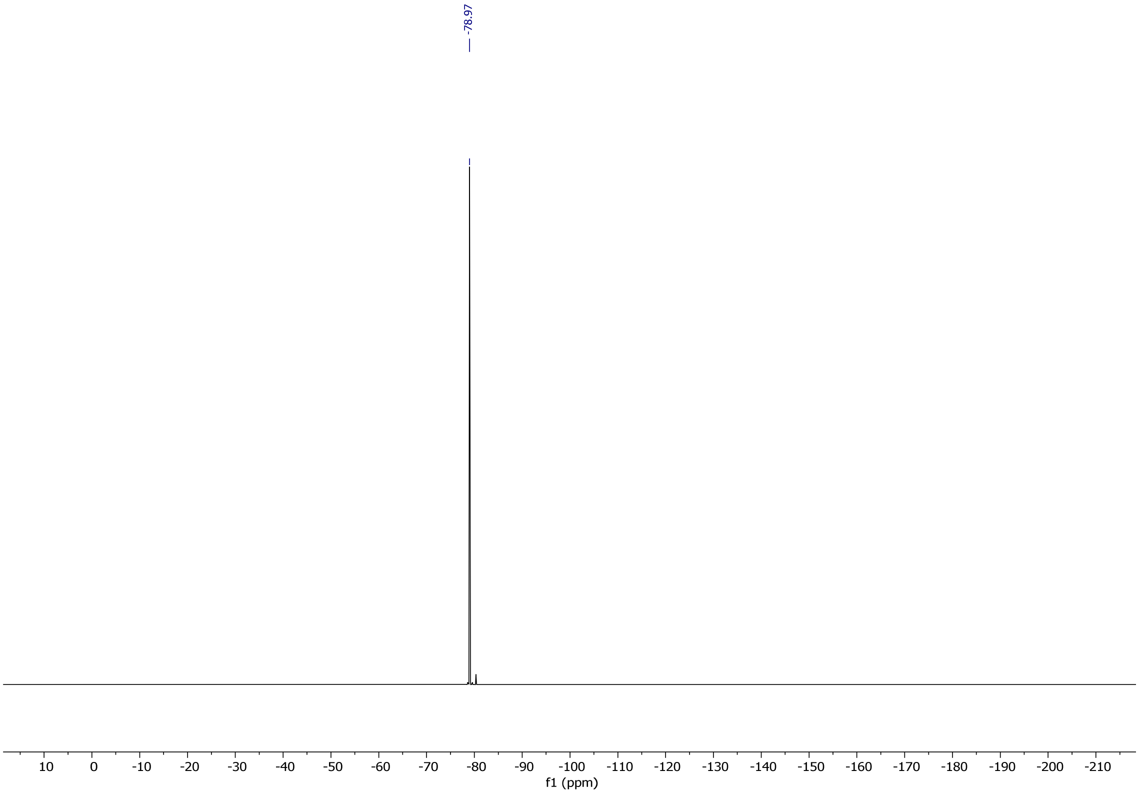

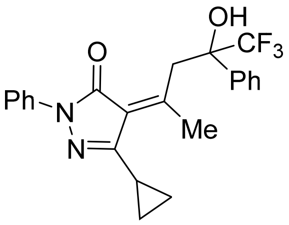

3da

^{1}H NMR (300 MHz, CDCl$_3$)

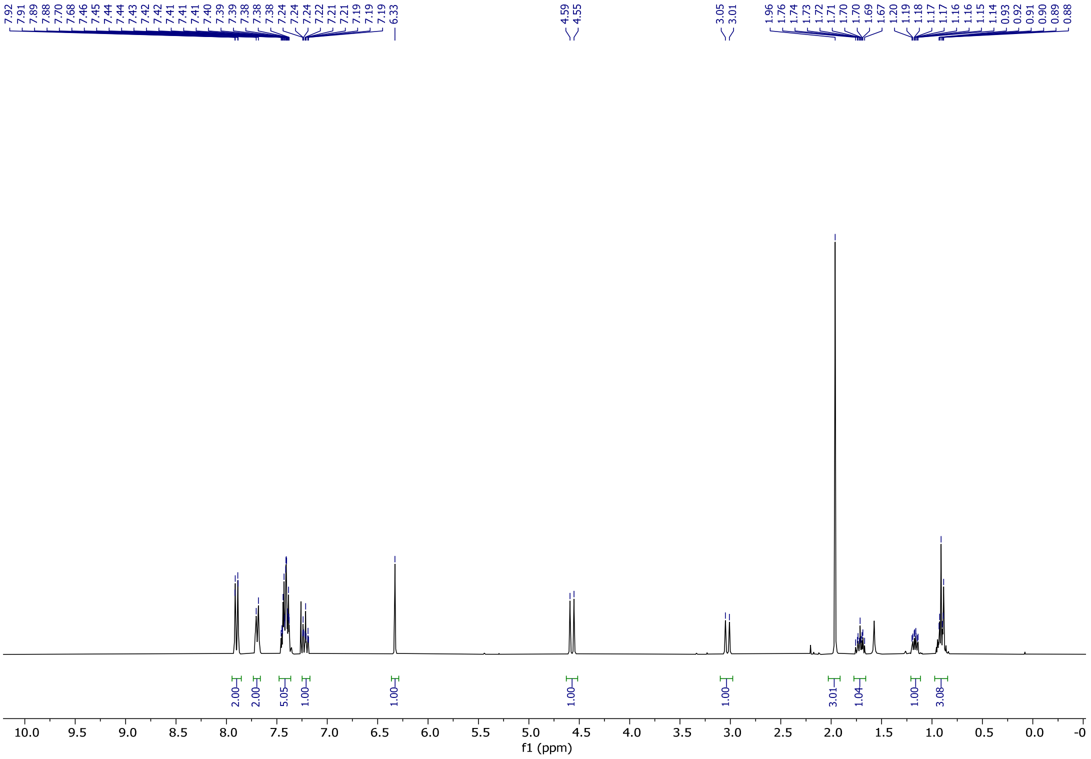

^{13}C {^{1}H} NMR (75 MHz, CDCl$_3$)

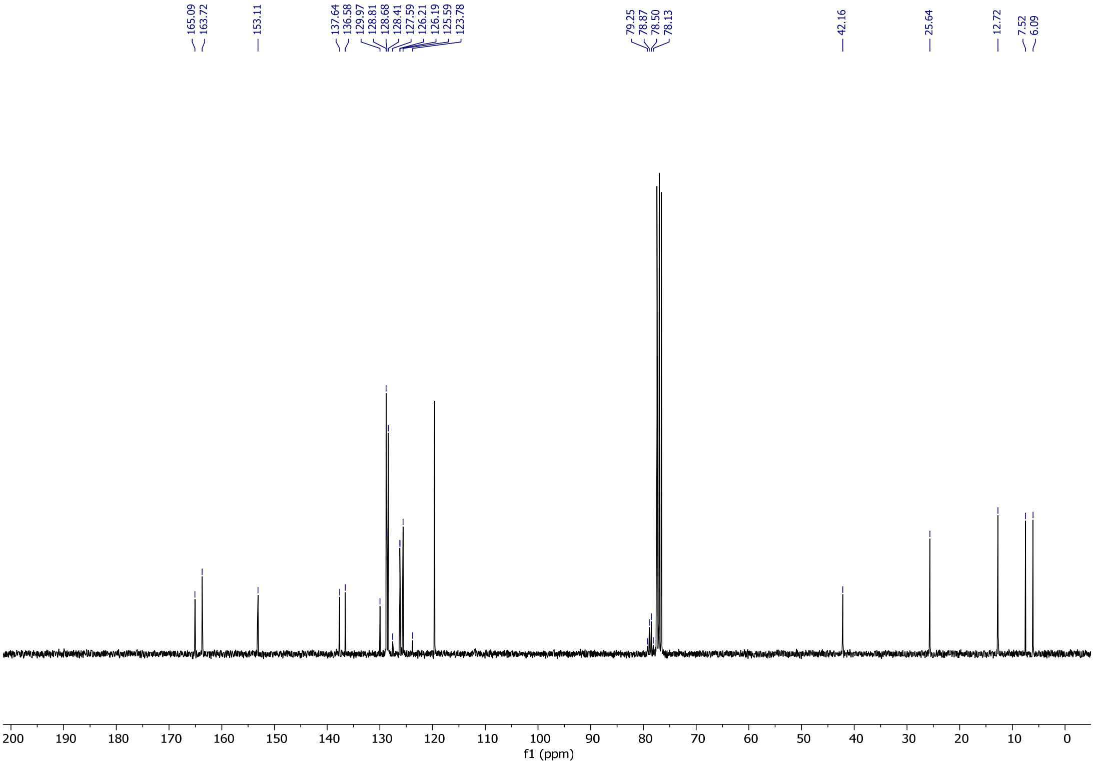

^{19}F NMR (282 MHz, $CDCl_3$)

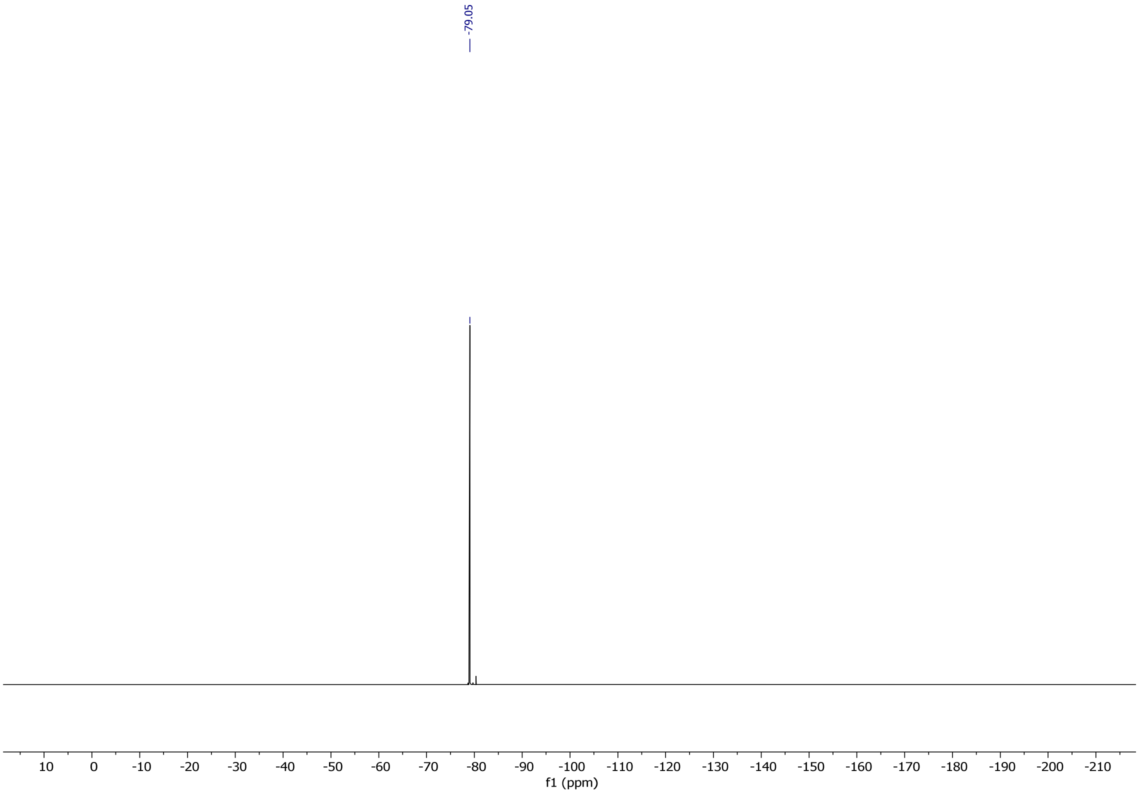

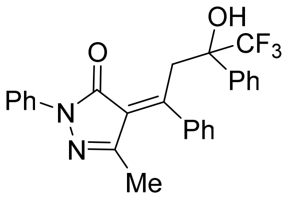

3ea

^{1}H NMR (300 MHz, CDCl$_3$)

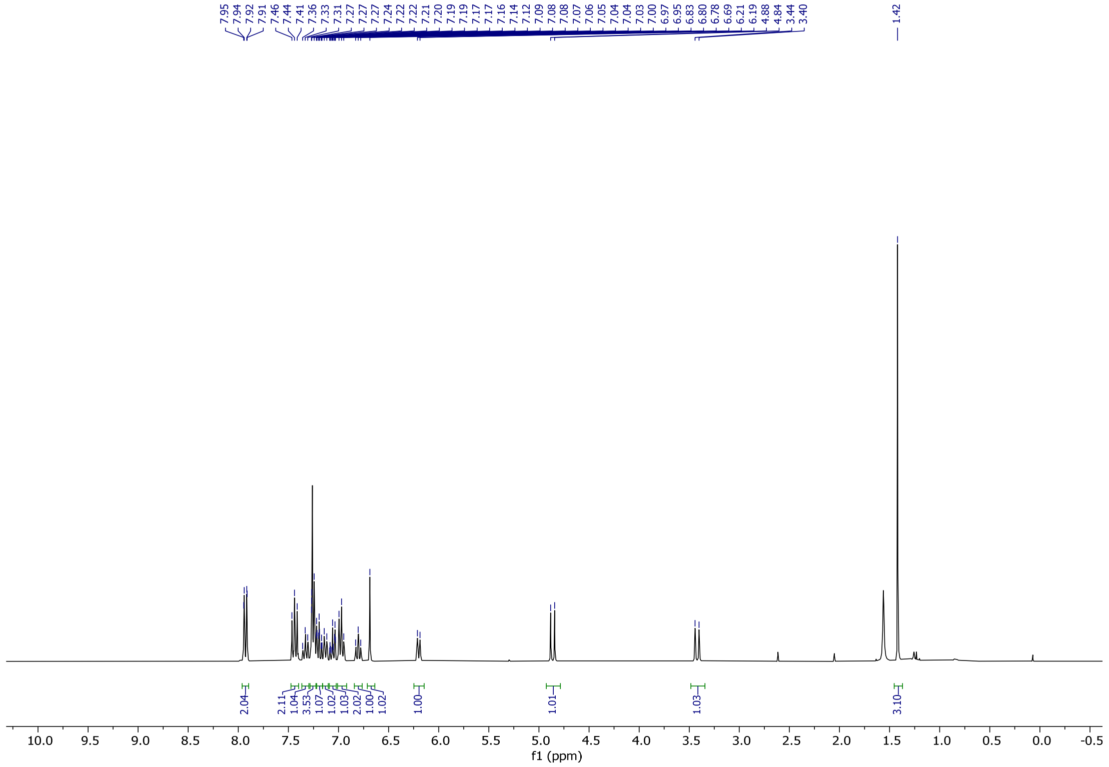

^{13}C {^{1}H} NMR (75 MHz, CDCl$_3$)

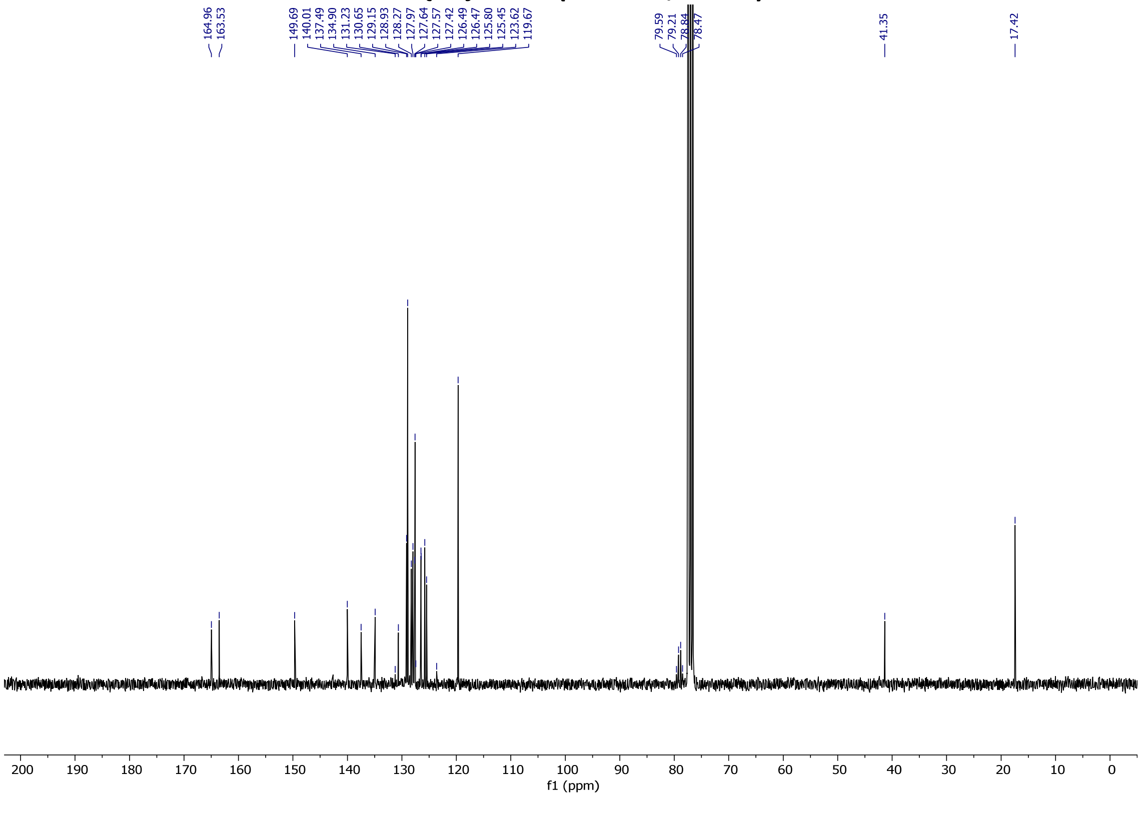

^{19}F NMR (282 MHz, $CDCl_3$)

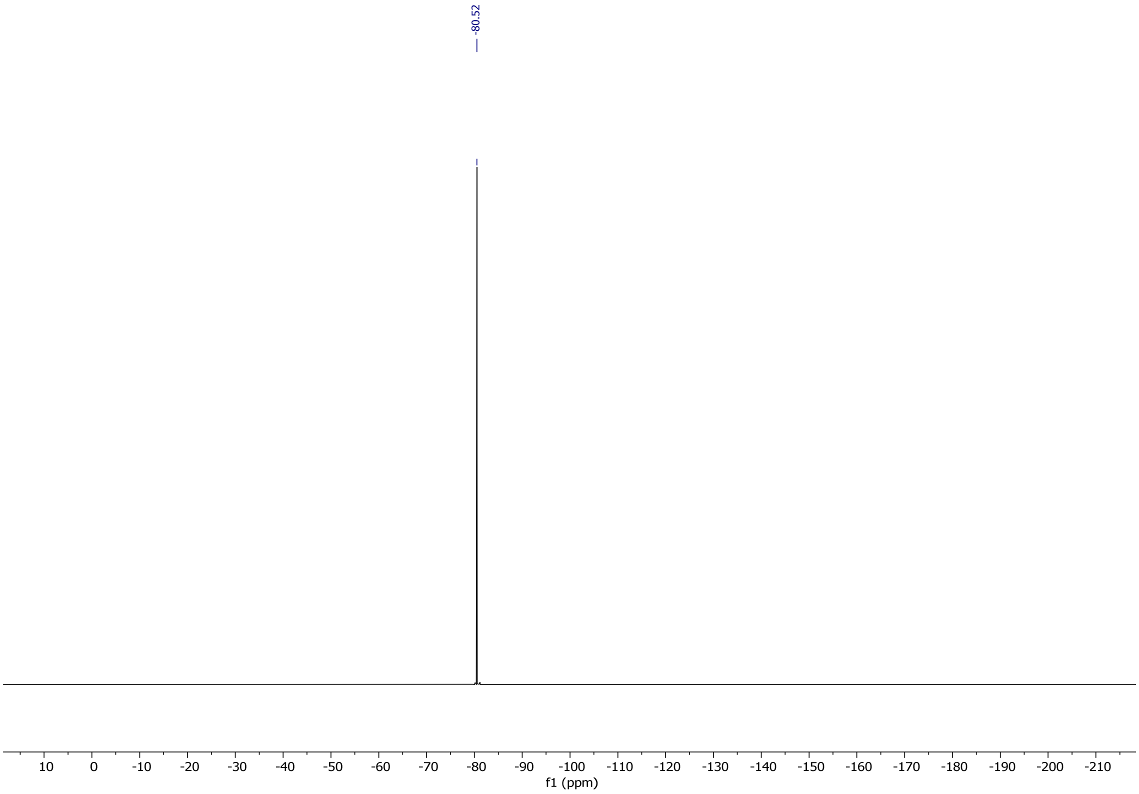

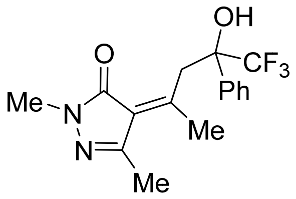

3fa

^{1}H NMR (300 MHz, $CDCl_3$)

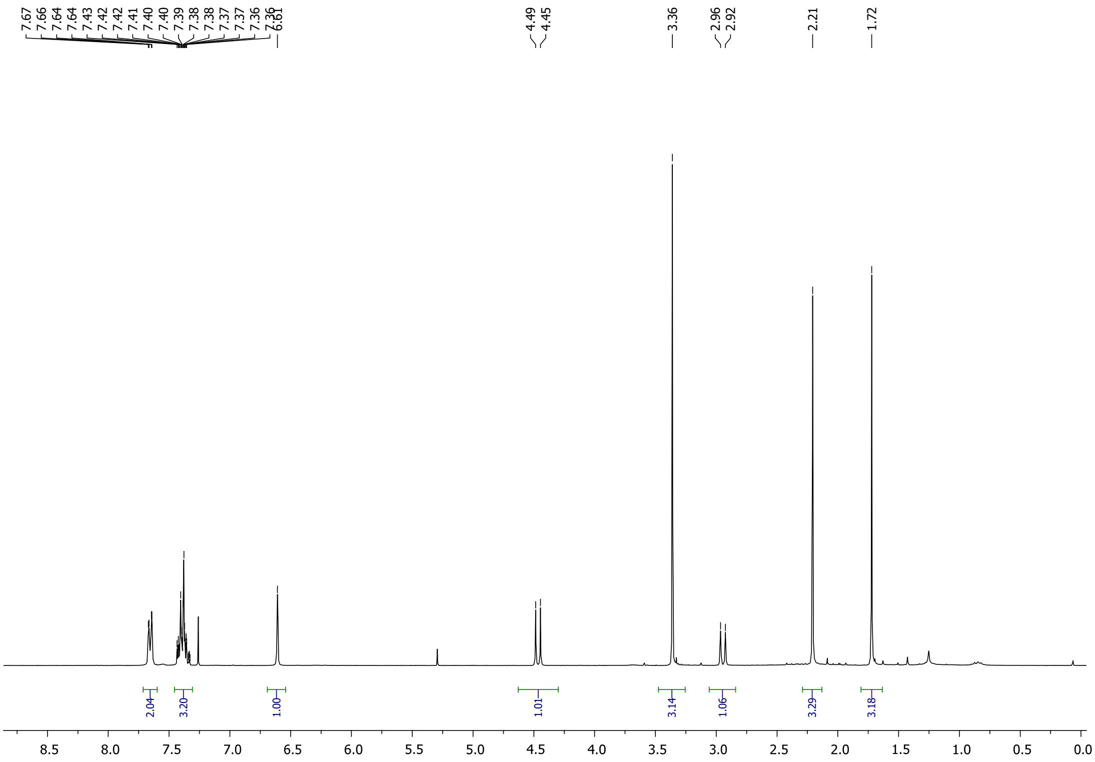

^{13}C {^{1}H} NMR (75 MHz, $CDCl_3$)

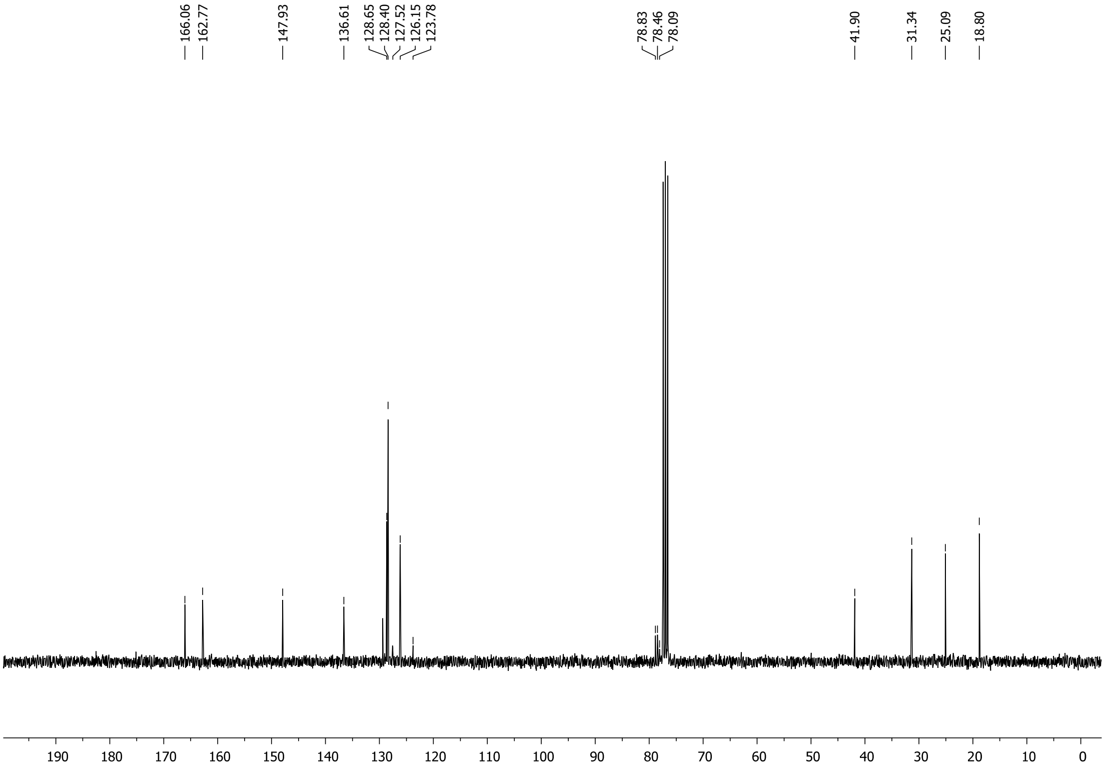

^{19}F NMR (282 MHz, $CDCl_3$)

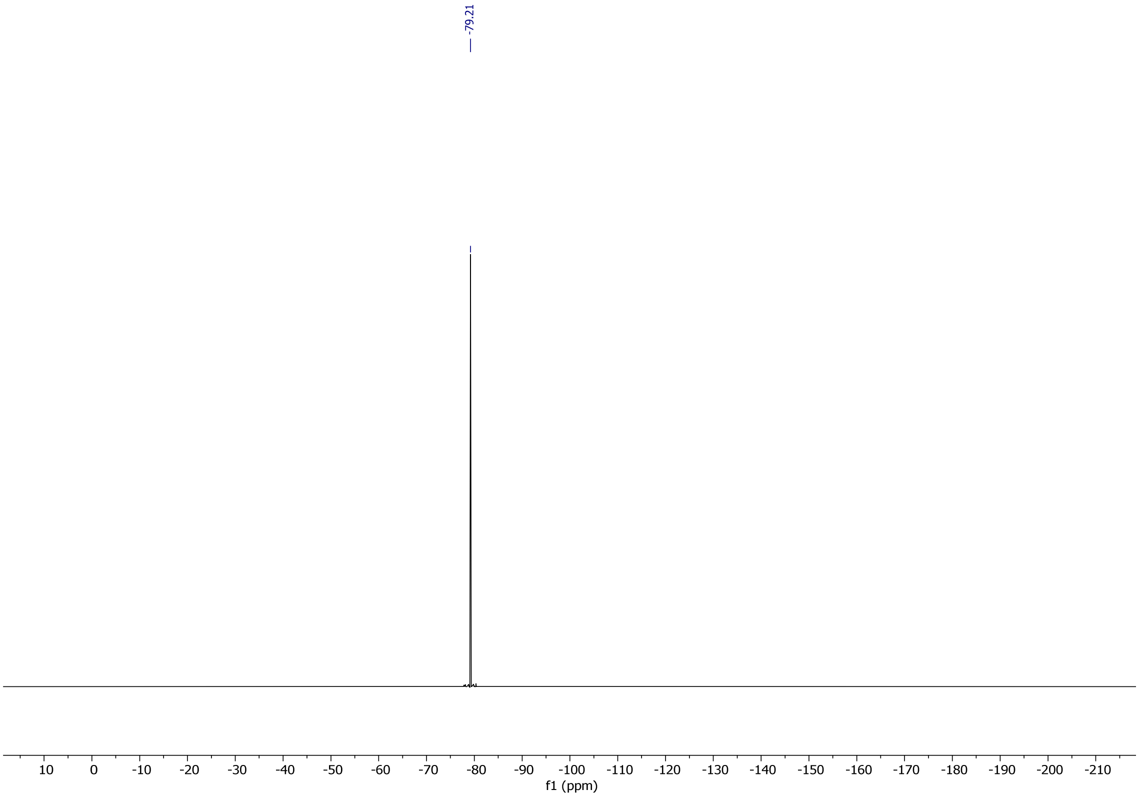

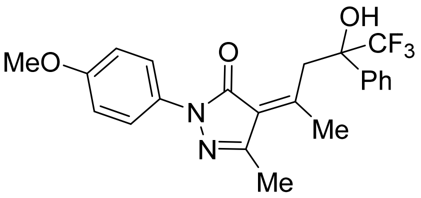

3ga

^{1}H NMR (300 MHz, CDCl$_3$)

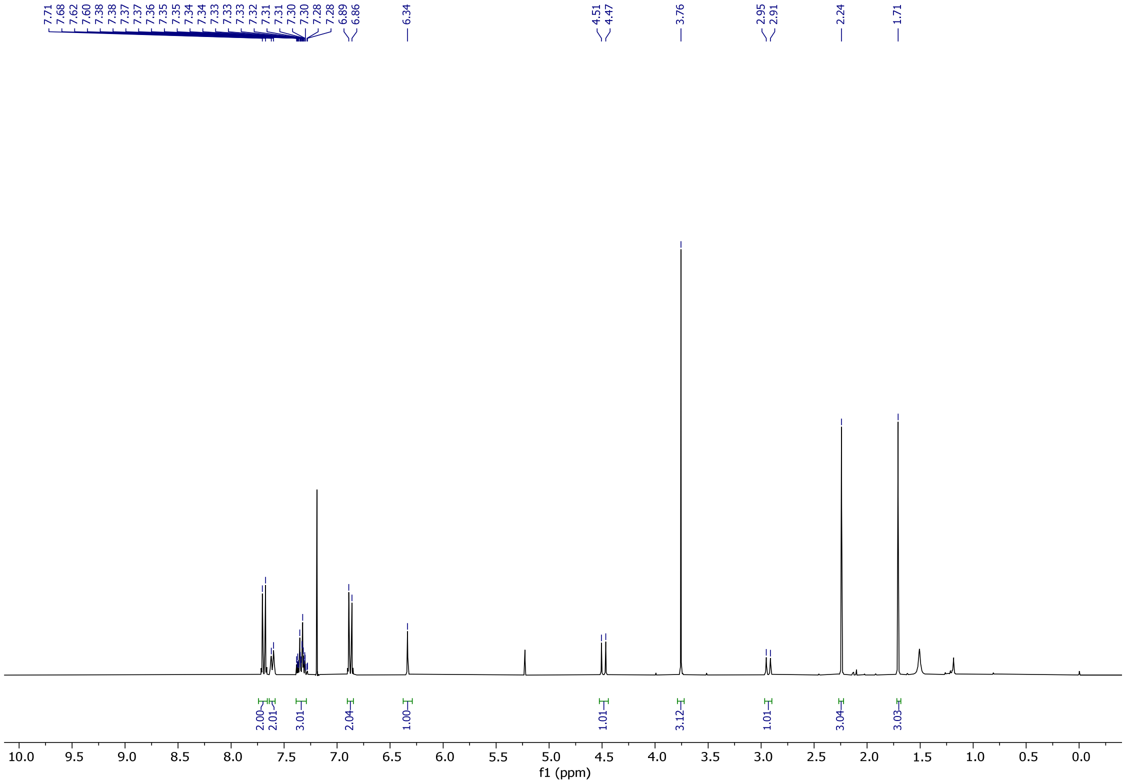

^{13}C {^{1}H} NMR (75 MHz, CDCl$_3$)

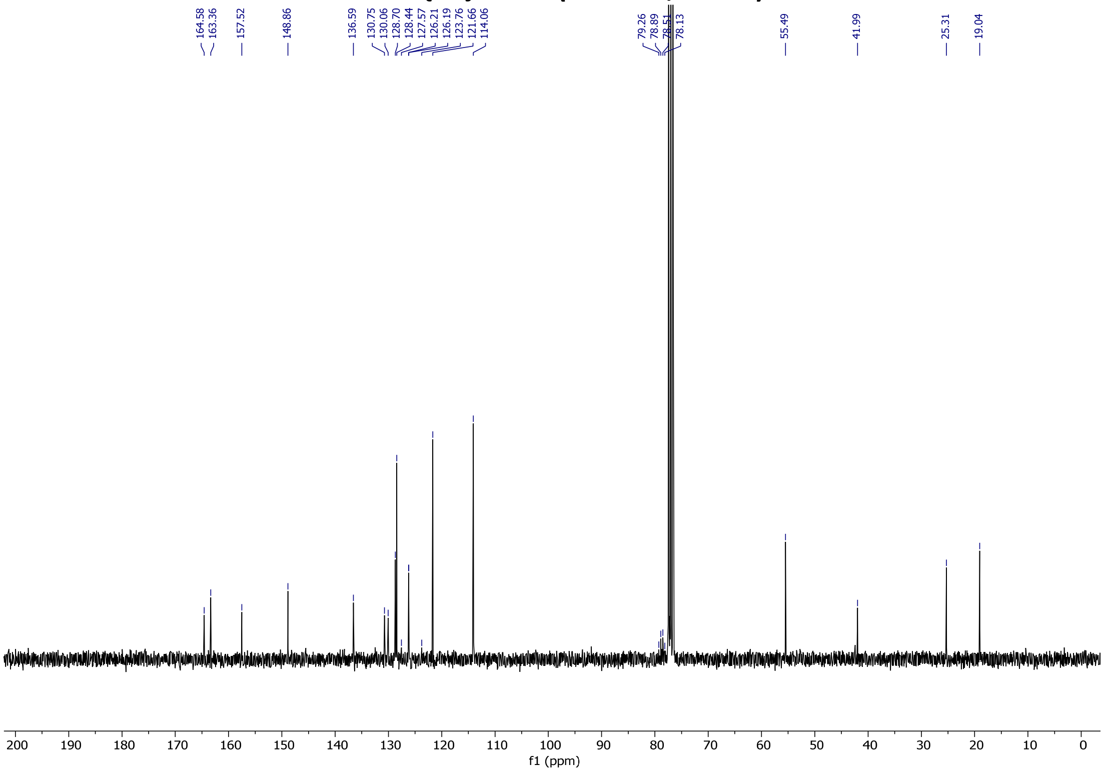

^{19}F NMR (282 MHz, $CDCl_3$)

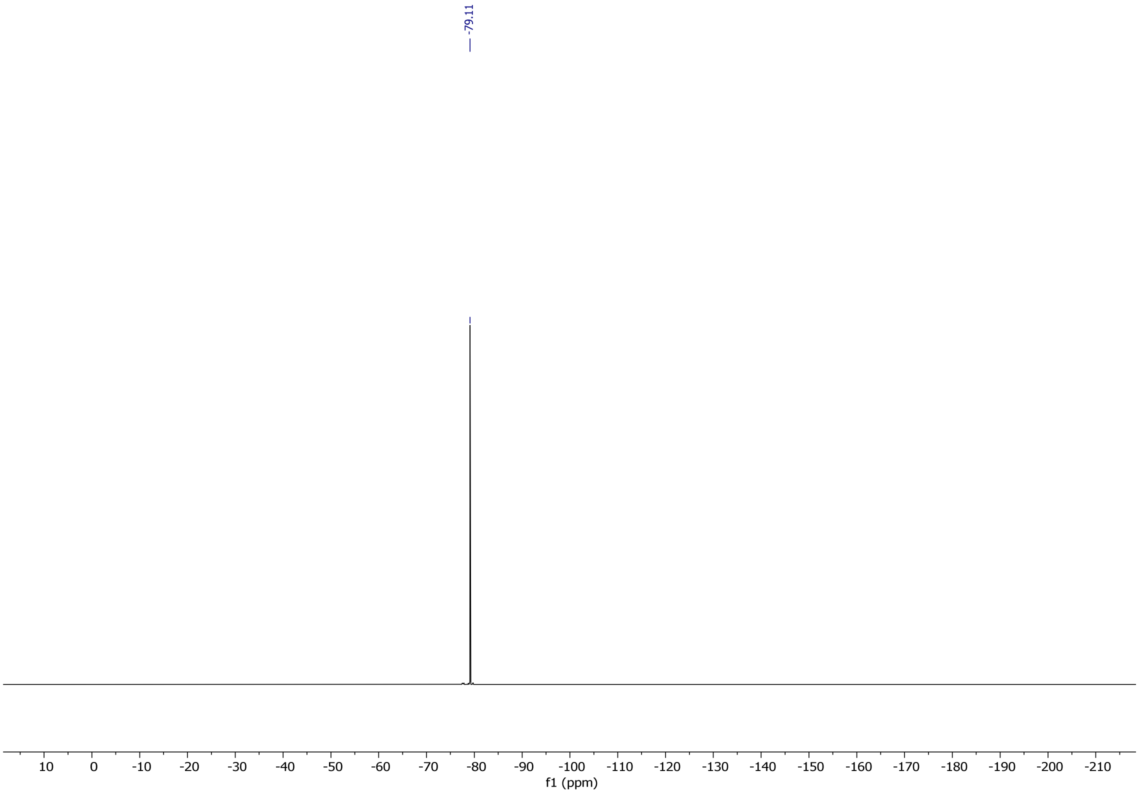

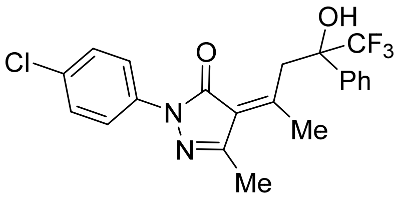

3ha

^{1}H NMR (300 MHz, $CDCl_3$)

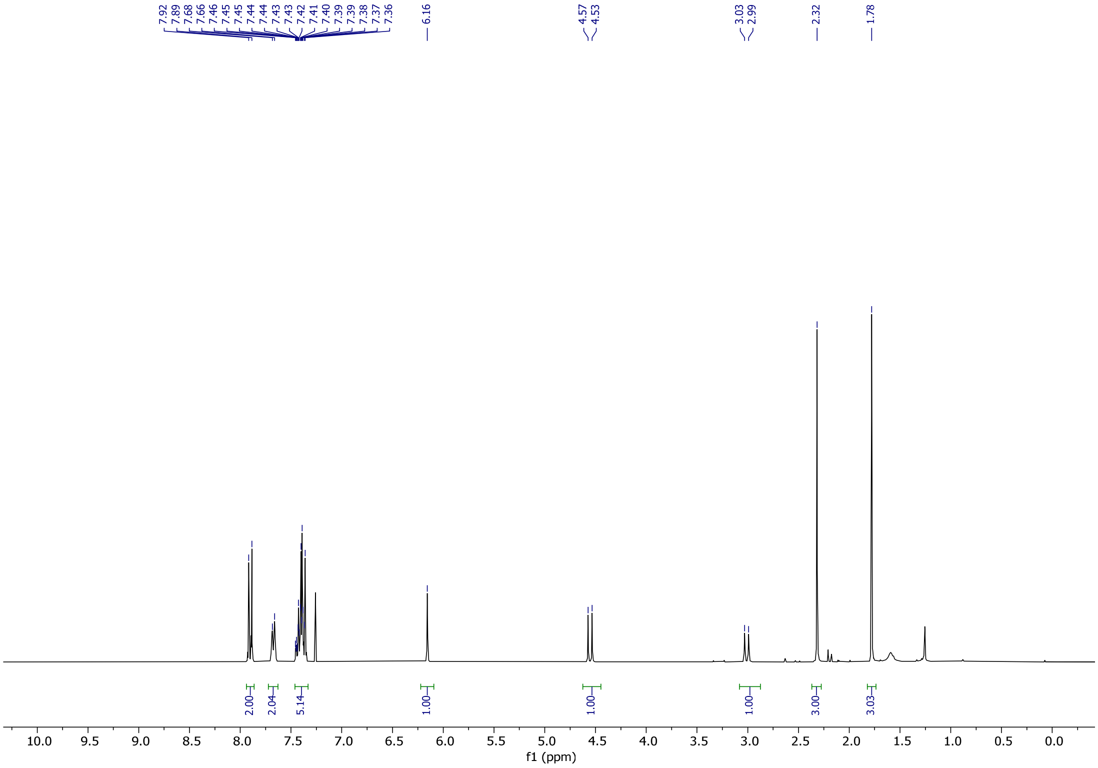

^{13}C {^{1}H} NMR (75 MHz, $CDCl_3$)

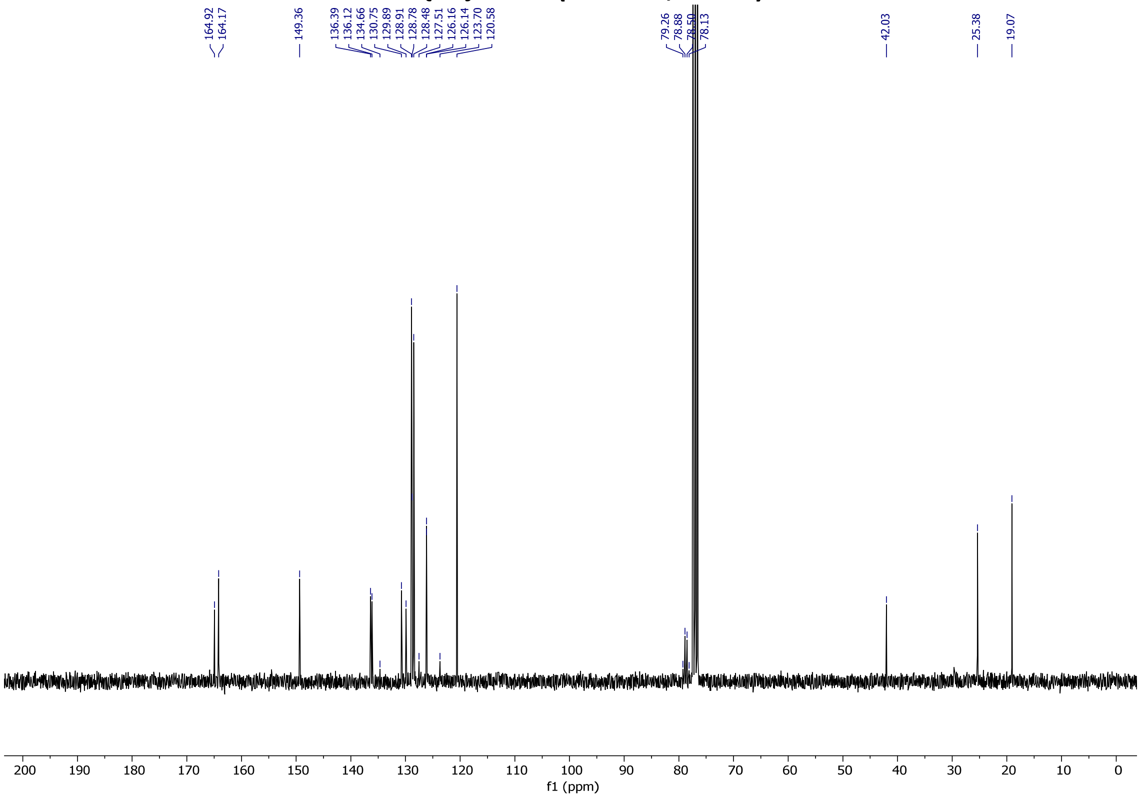

^{19}F NMR (282 MHz, $CDCl_3$)

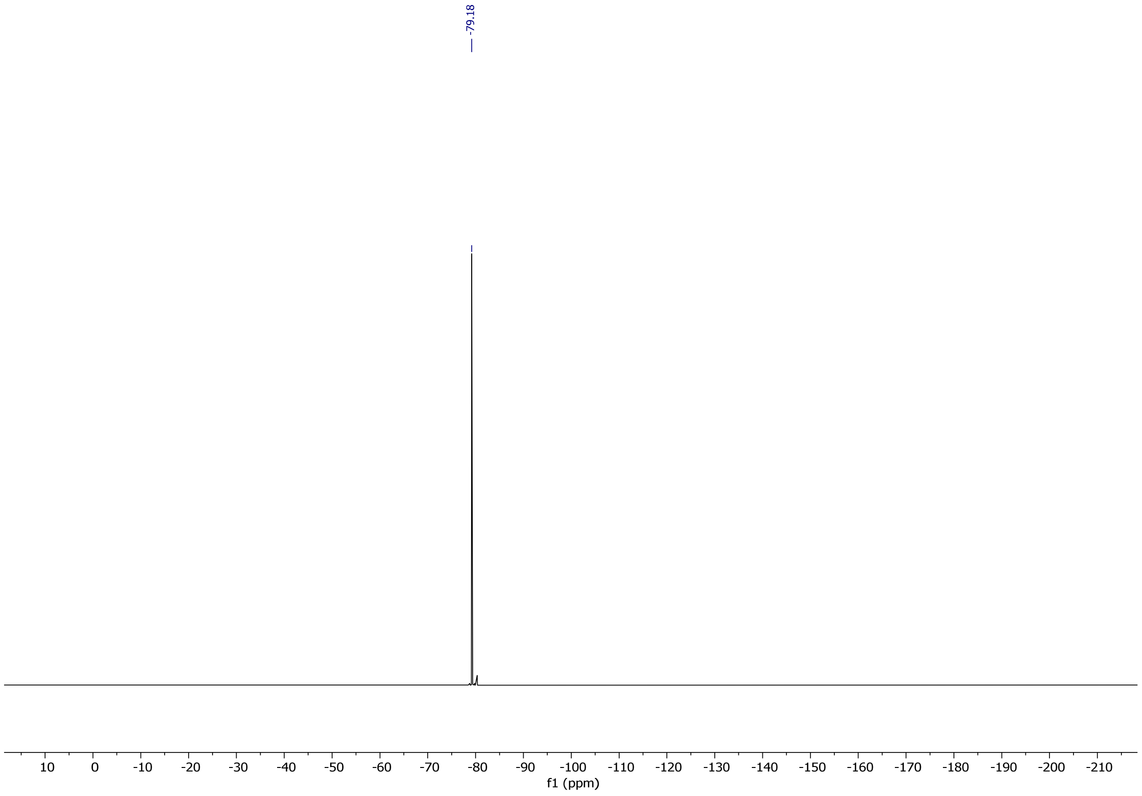

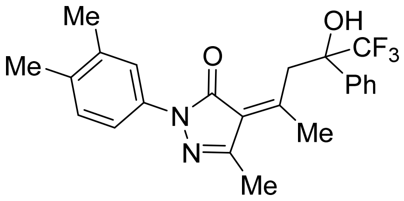

3ia

^{1}H NMR (300 MHz, CDCl$_3$)

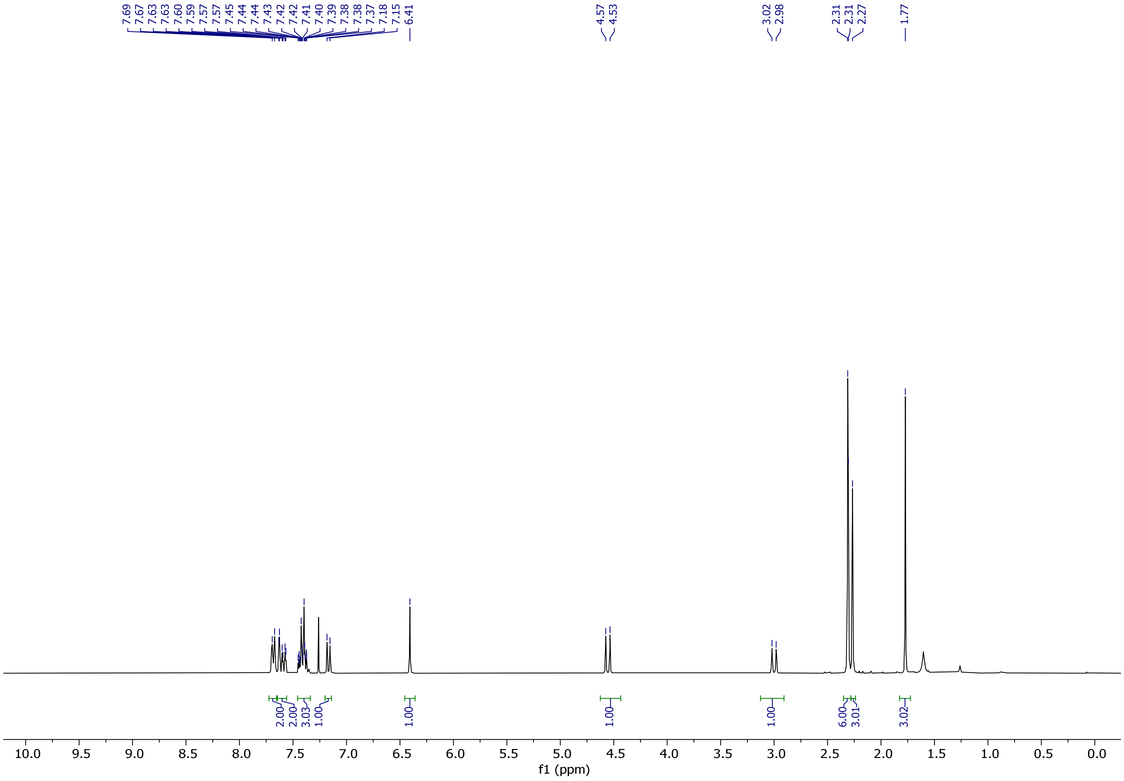

^{13}C {^{1}H} NMR (75 MHz, CDCl$_3$)

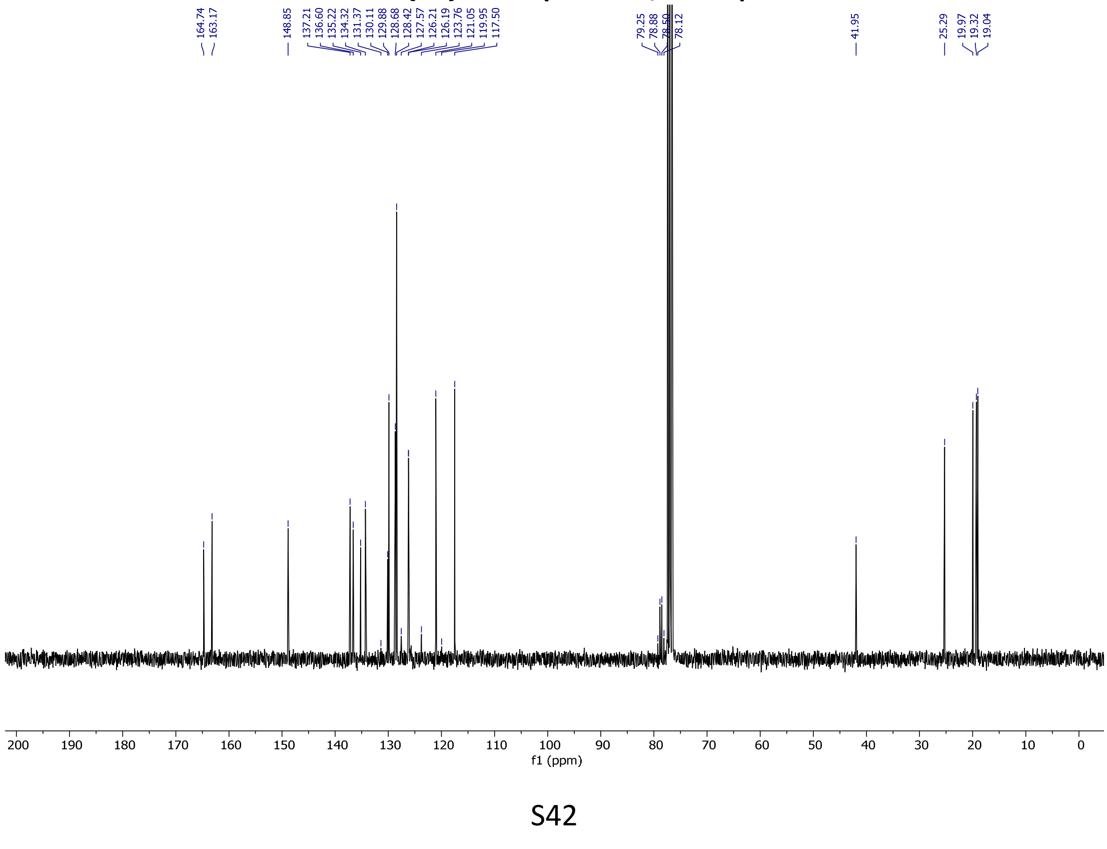

^{19}F NMR (282 MHz, $CDCl_3$)

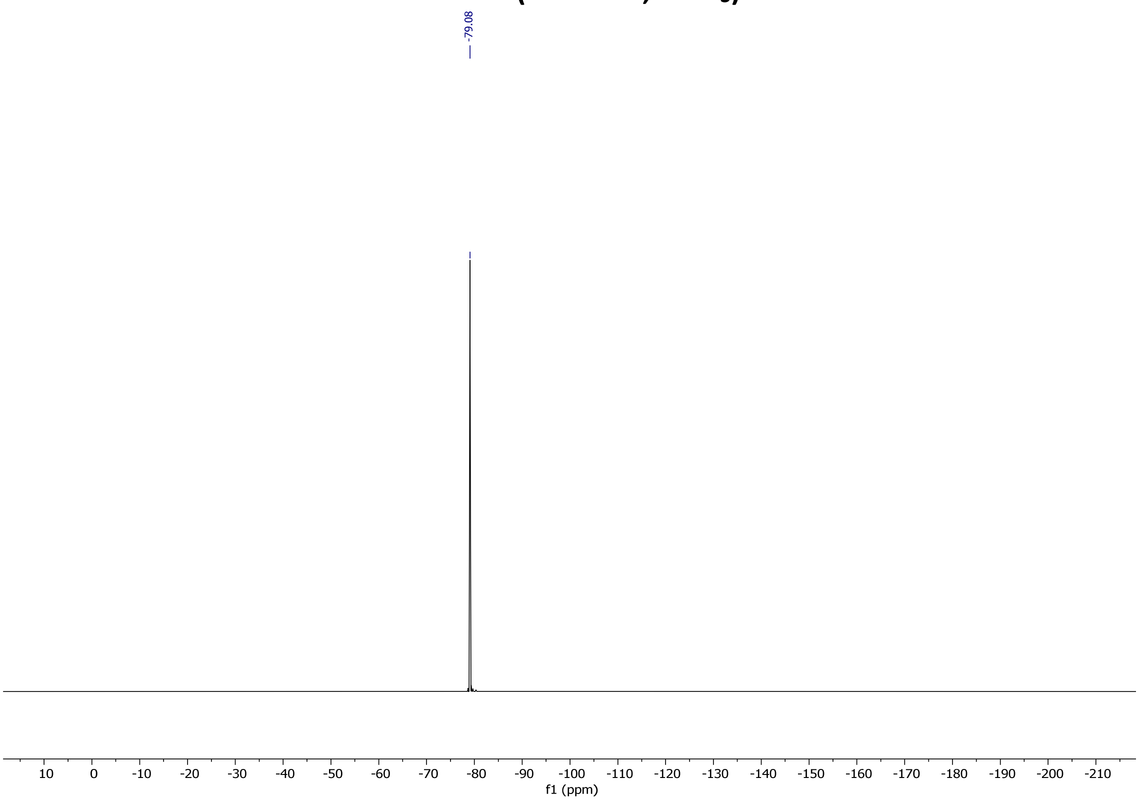

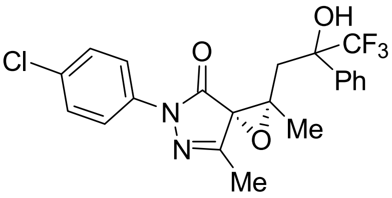

4 major

^{1}H NMR (300 MHz, $CDCl_3$)

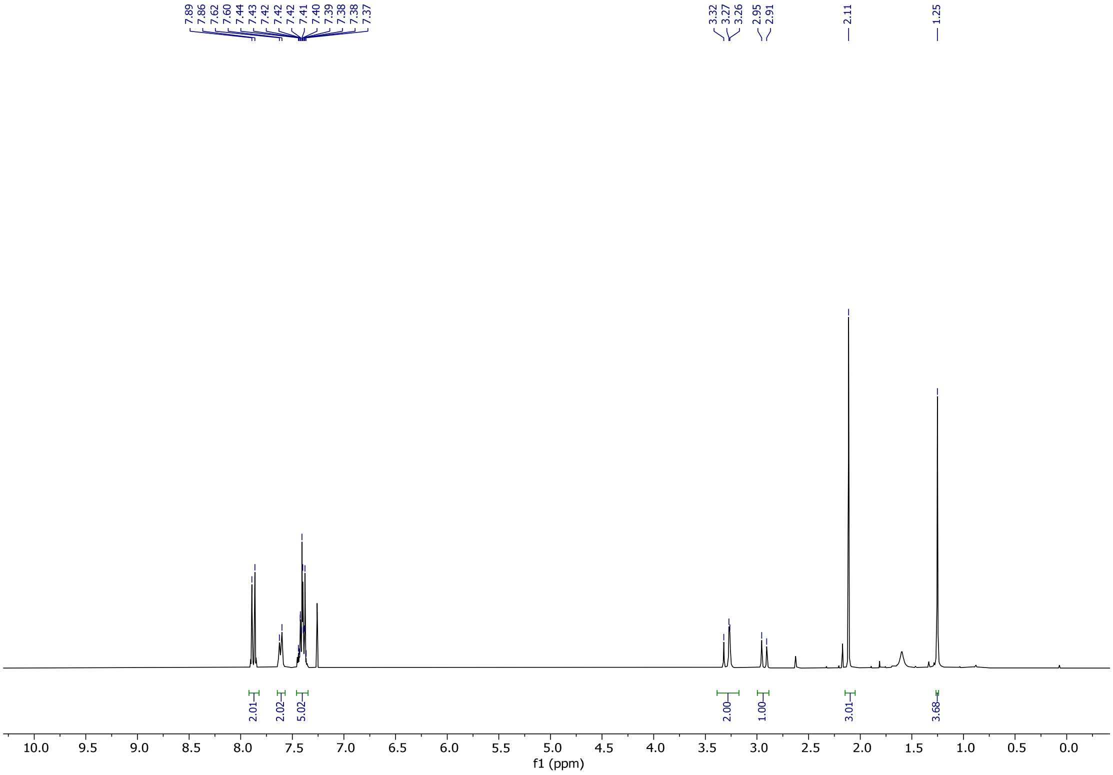

^{13}C {^{1}H} NMR (75 MHz, $CDCl_3$)

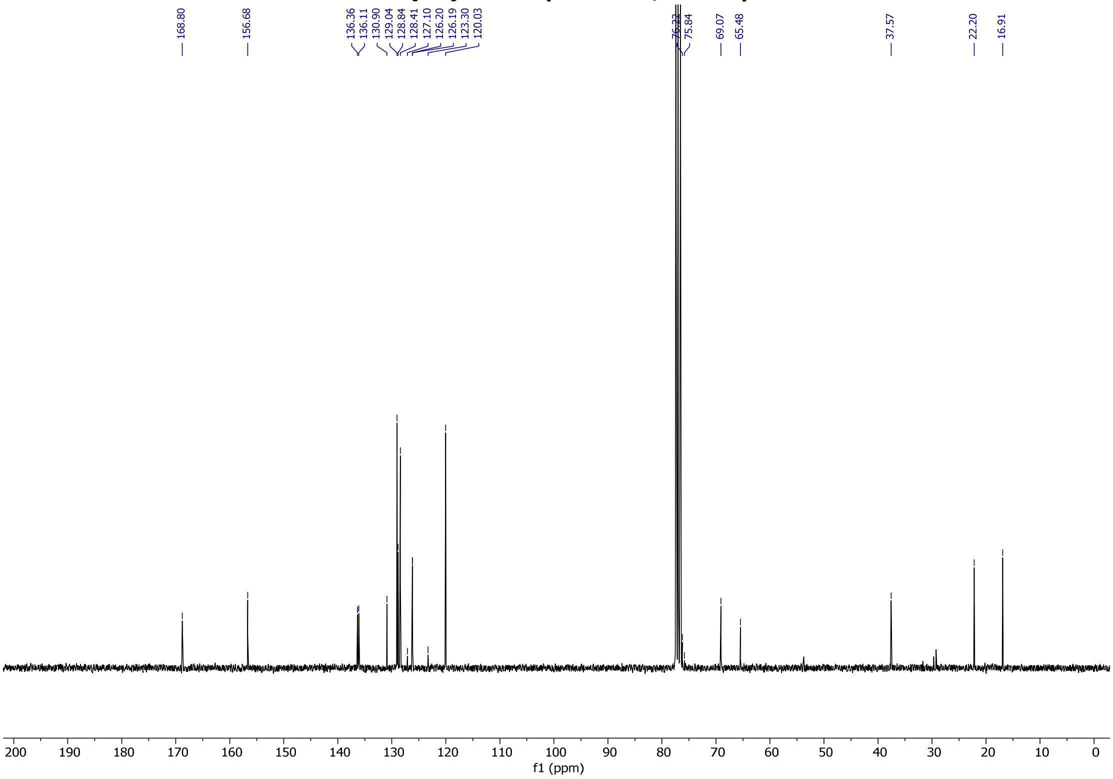

^{19}F NMR (282 MHz, $CDCl_3$)

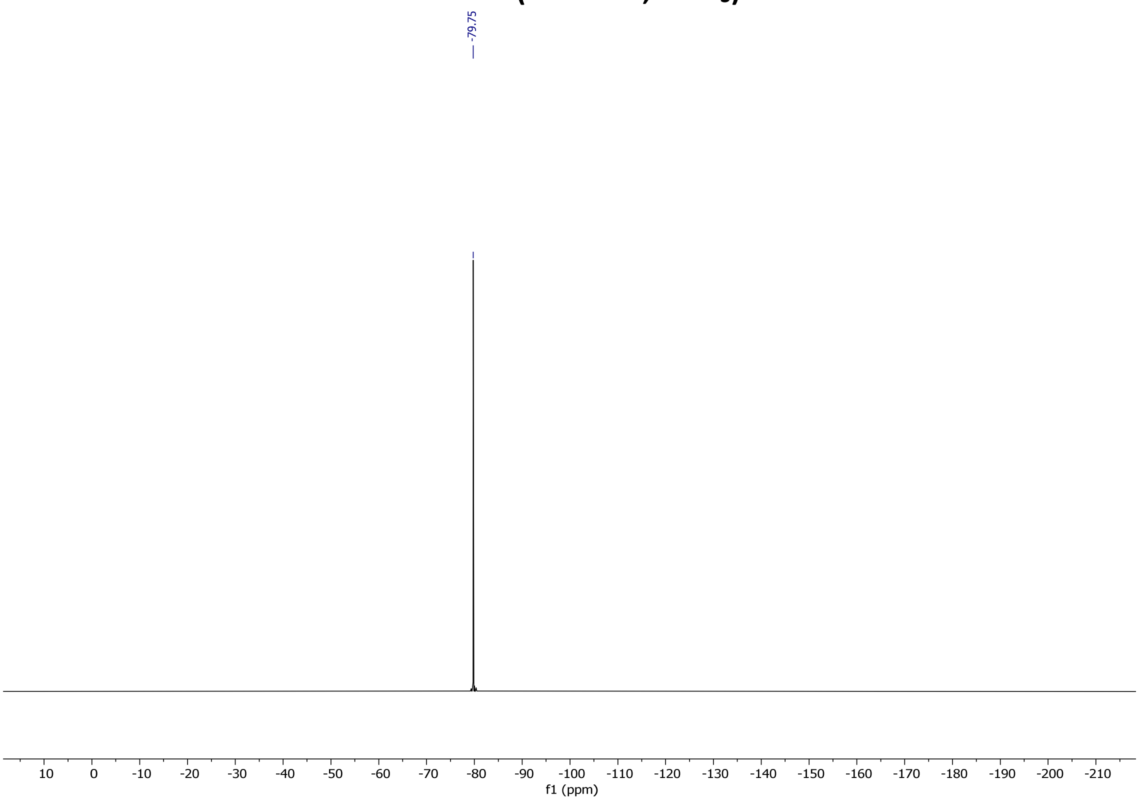

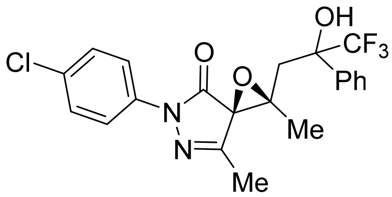

4 minor

^{1}H NMR (300 MHz, CDCl$_3$)

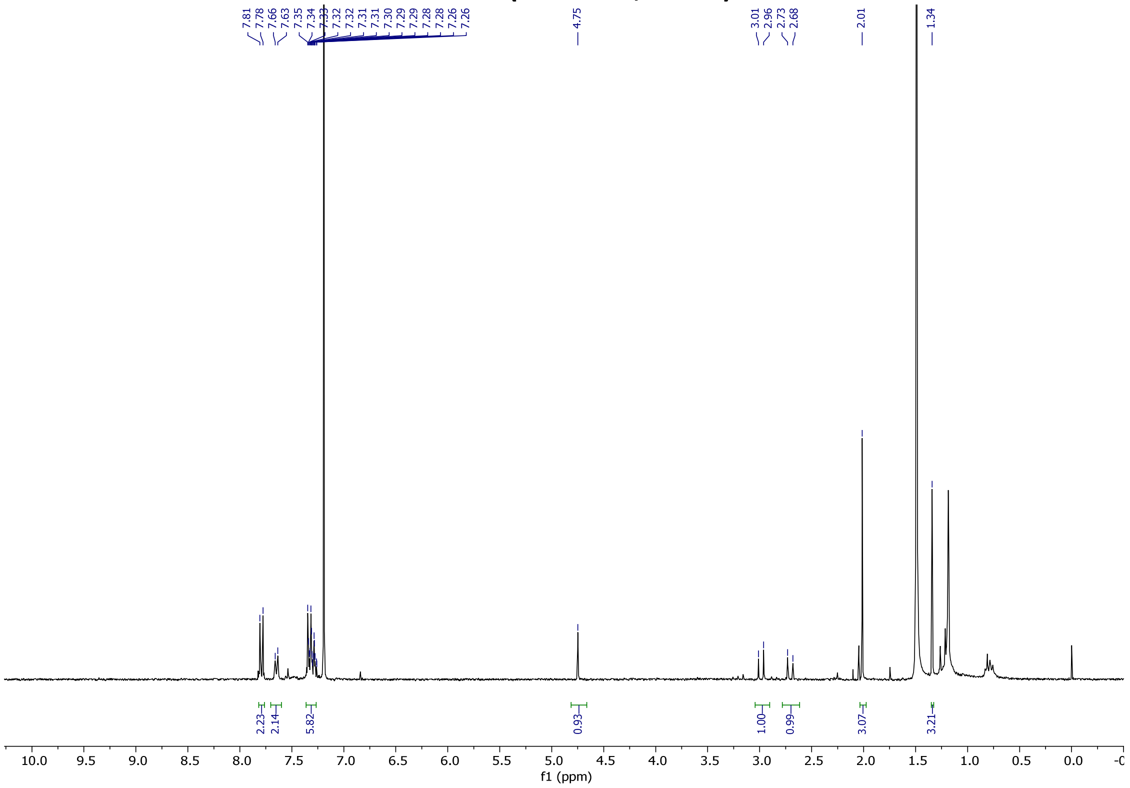

^{19}F NMR (282 MHz, CDCl$_3$)

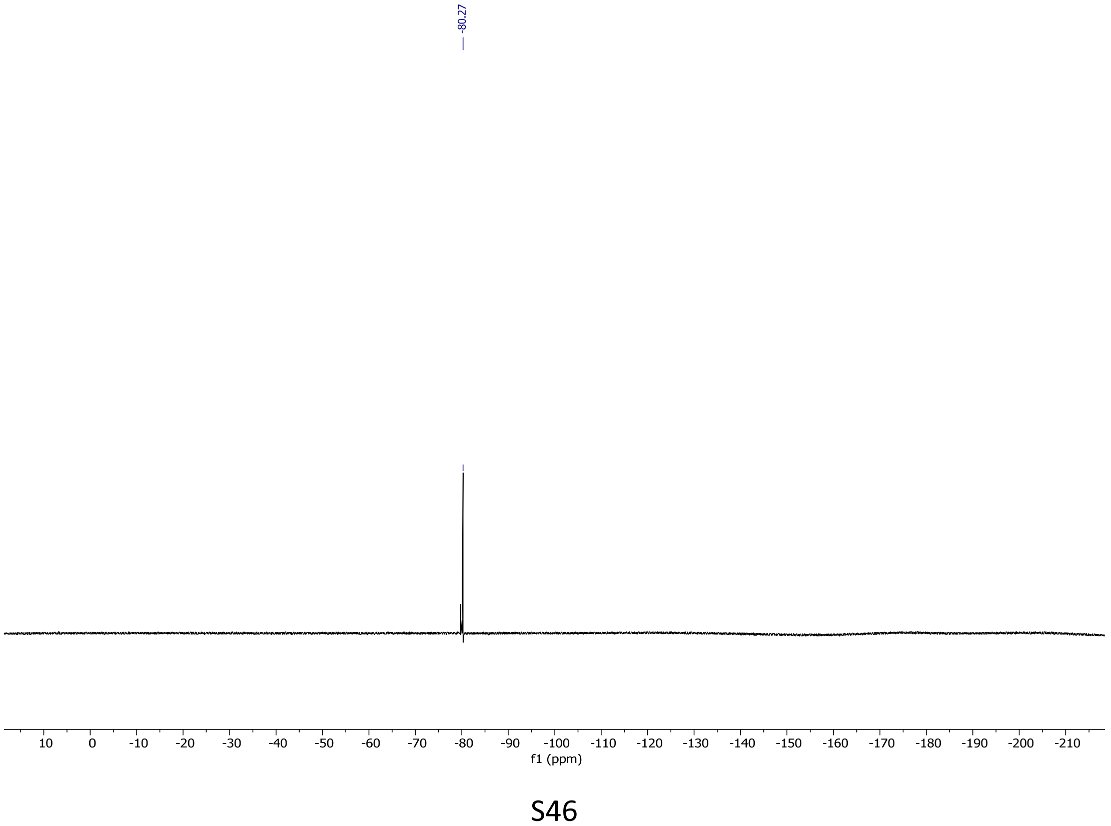

8. HPLC DATA

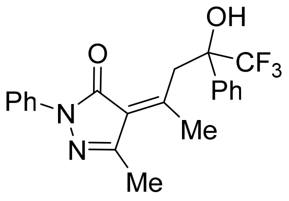

3aa

Sample Name: LC-605 ADH 9010 1mL Vial Number: 1

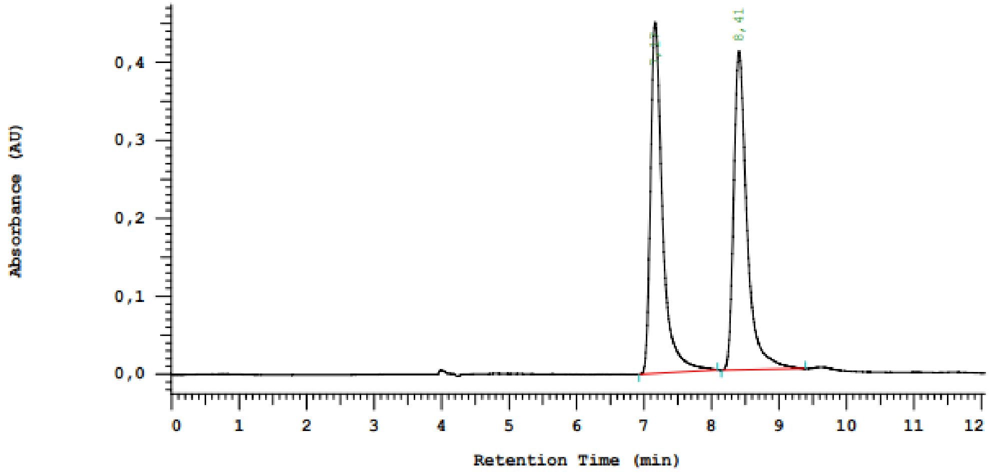

No.	RT	Area	Area %	Name
1	7,17	2783130	49,926	
2	8,41	2791410	50,074	
		5574540	100,000	

Sample Name: LC-603 ADH 9010 1mL Vial Number: 1

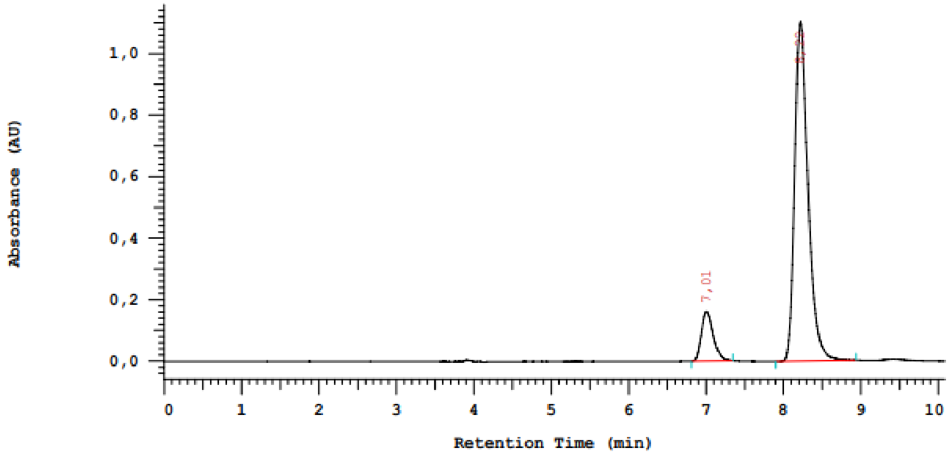

No.	RT	Area	Area %	Name
1	7,01	850610	11,409	
2	8,22	6605100	88,591	
		7455710	100,000	

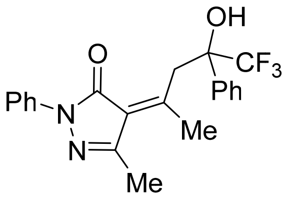

3aa (result at 0.2M concentration)

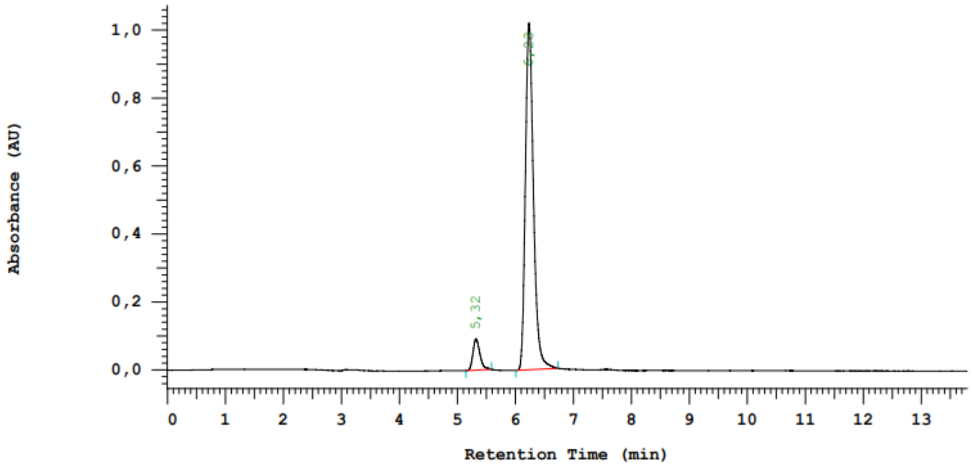

No.	RT	Area	Area %	Name
1	5,32	382210	7,167	
2	6,23	4950775	92,833	
		5332985	100,000	

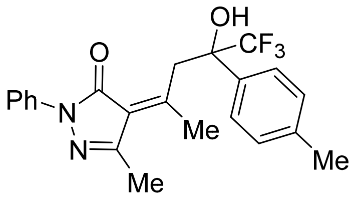

3ba

Sample Name: AG-74 ADH 9010 1mL Vial Number: 1

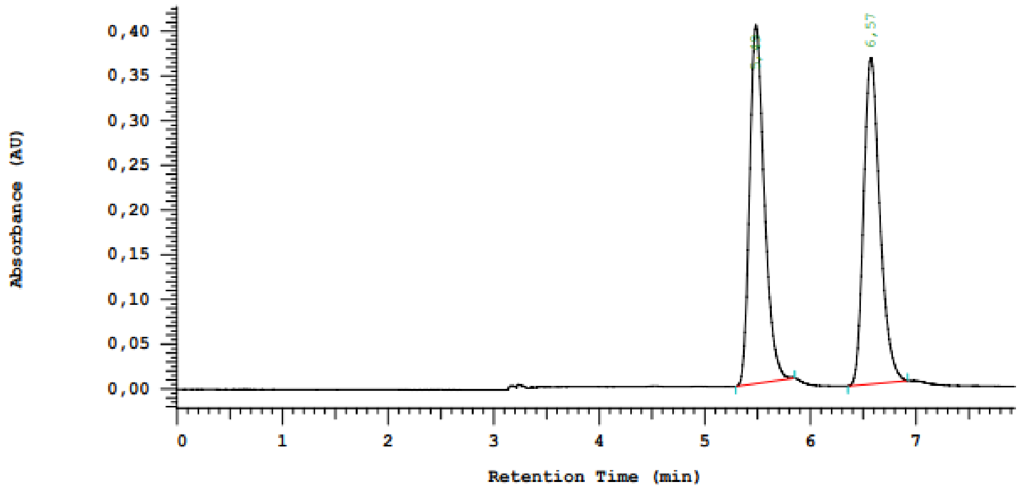

No.	RT	Area	Area %	Name
1	5,49	1952880	49,884	
2	6,57	1961955	50,116	
		3914835	100,000	

Sample Name: AG-117 ADH 9010 1mL Vial Number: 1

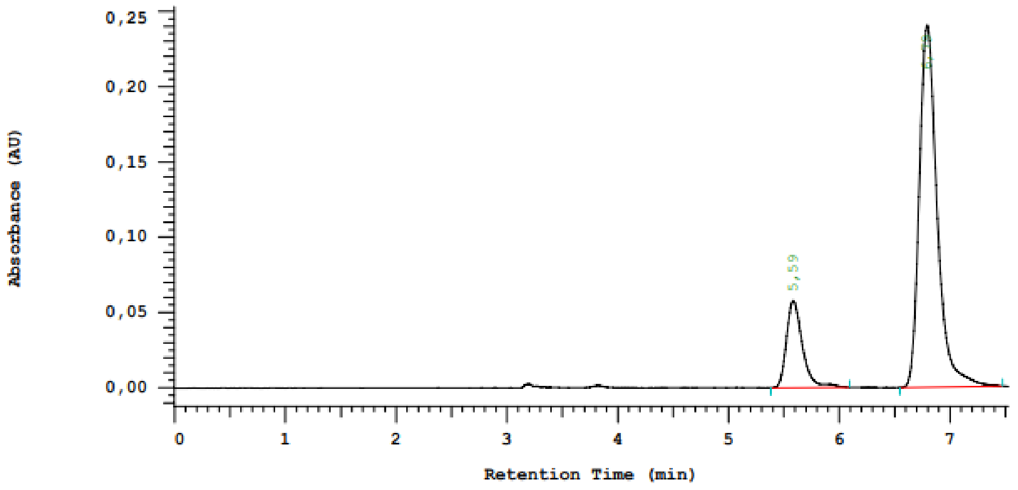

No.	RT	Area	Area %	Name
1	5,59	289595	17,678	
2	6,79	1348575	82,322	
		1638170	100,000	

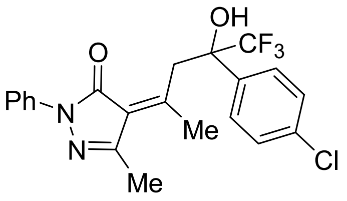

3ac

Sample Name: AG-76 ADH 9010 1mL Vial Number: 1

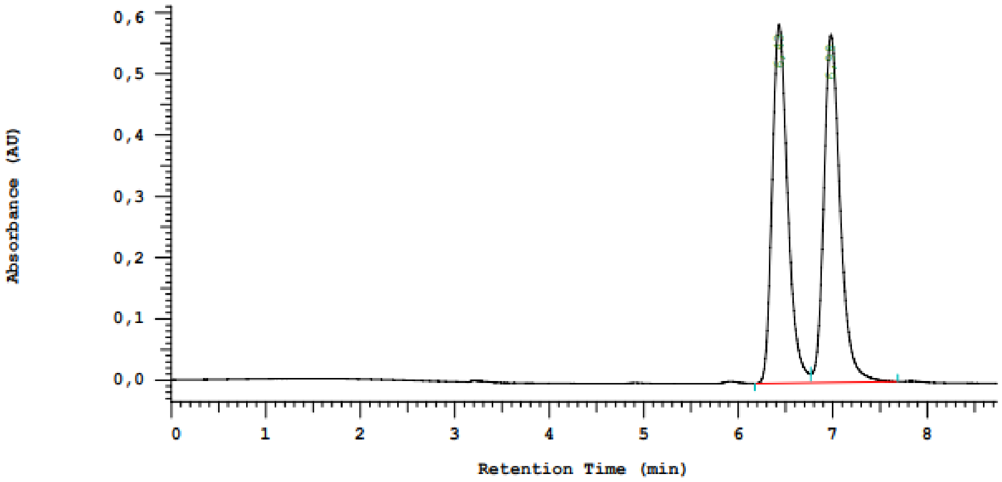

No.	RT	Area	Area %	Name
1	6,43	3330573	49,223	
2	6,98	3435776	50,777	
		6766349	100,000	

Sample Name: AG-118 ADH 9010 1mL Vial Number: 1

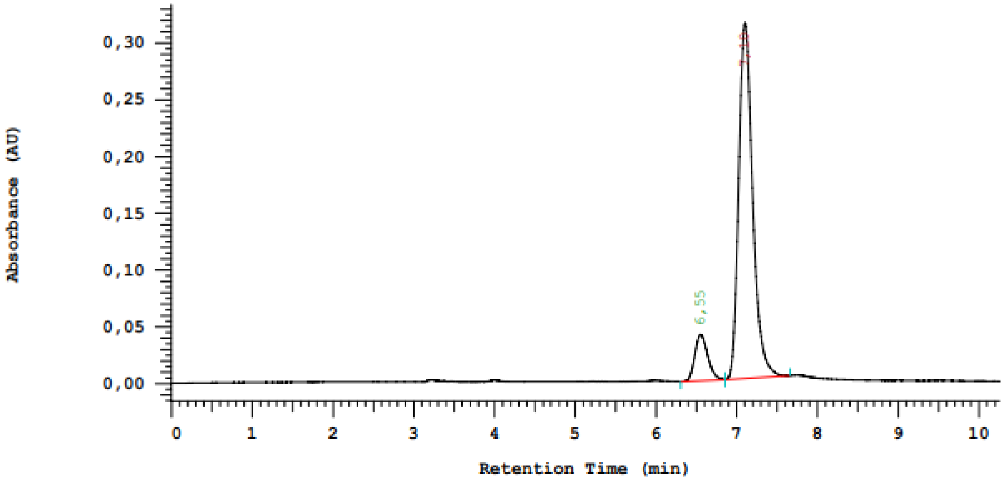

No.	RT	Area	Area %	Name
1	6,55	223170	10,683	
2	7,10	1865935	89,317	
		2089105	100,000	

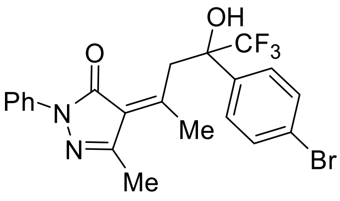

3ad

Sample Name: AG-82 ADH 9010 1mL   Vial Number: 1

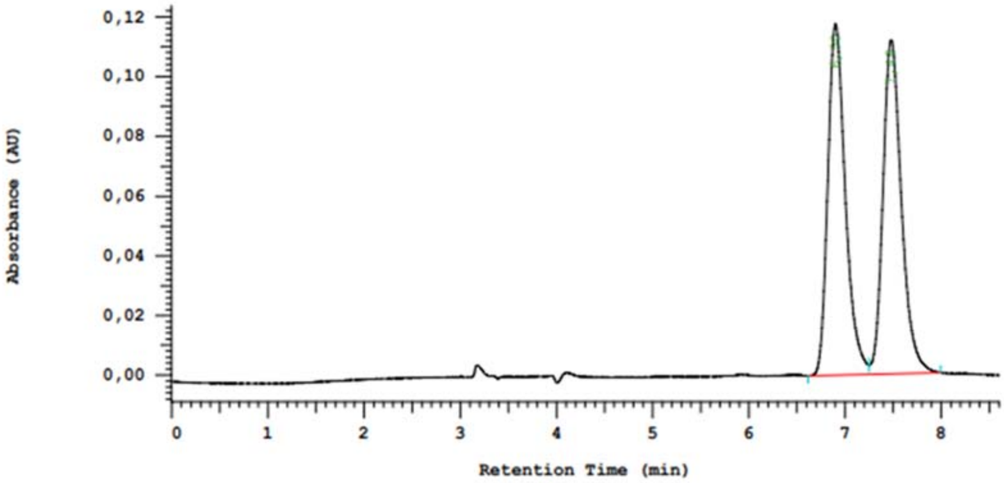

No.	RT	Area	Area %	Name
1	6,90	745392	49,905	
2	7,48	748242	50,095	
		1493634	100,000	

Sample Name: LC-840 ADH 9010 1mL   Vial Number: 1

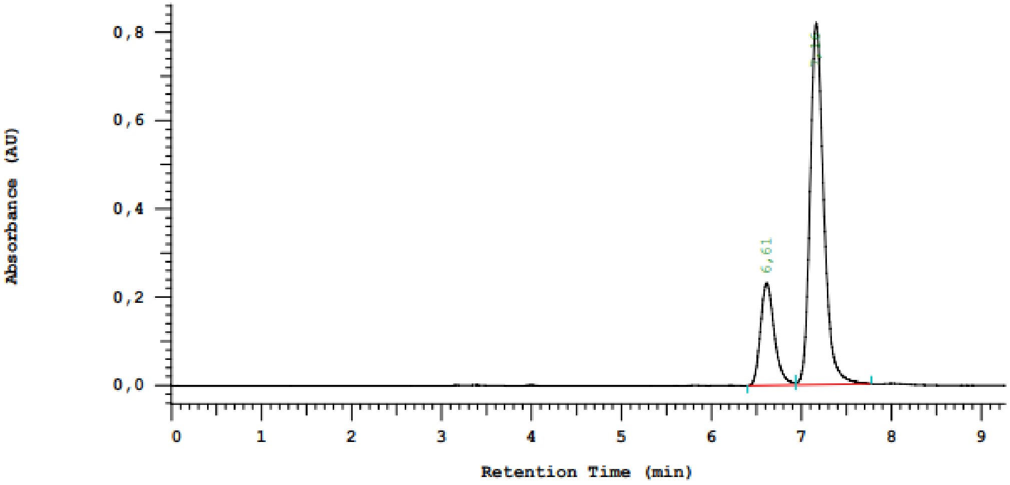

No.	RT	Area	Area %	Name
1	6,61	1244131	22,175	
2	7,16	4366328	77,825	
		5610459	100,000	

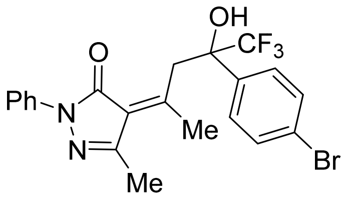

3ad (result at 0.2 M concentration)

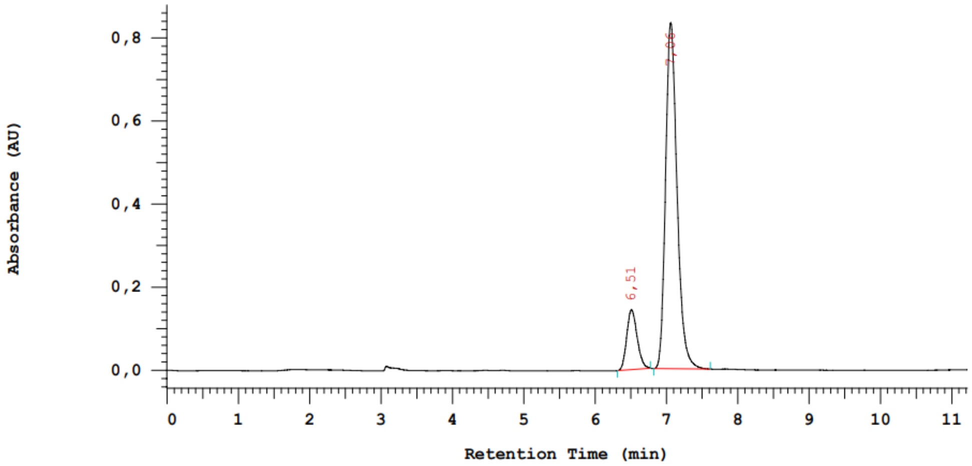

No.	RT	Area	Area %	Name
1	6,51	726445	13,536	
2	7,06	4640360	86,464	
		5366805	100,000	

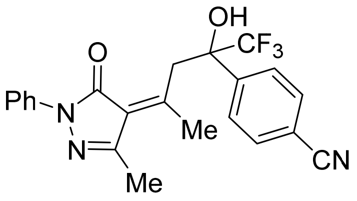

3ae

Sample Name: AG-99 ADH 9010 1mL Vial Number: 1

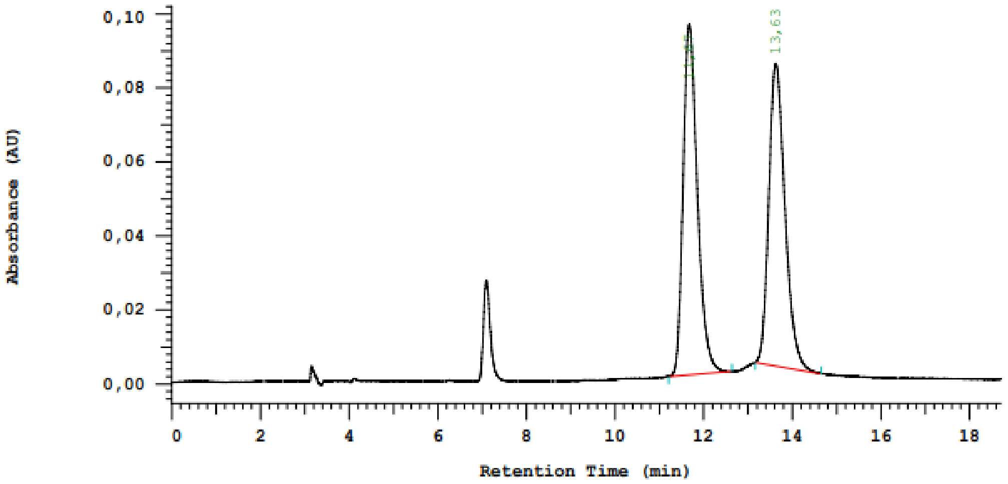

No.	RT	Area	Area %	Name
1	11,67	1053350	50,391	
2	13,63	1036990	49,609	
		2090340	100,000	

Sample Name: AG-122 ADH 9010 1mL Vial Number: 1

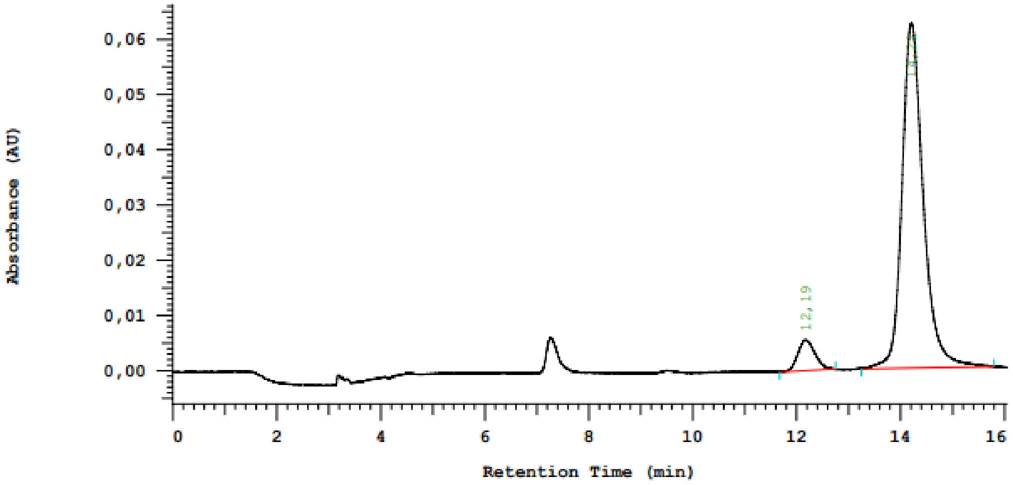

No.	RT	Area	Area %	Name
1	12,19	63450	6,658	
2	14,21	889560	93,342	
		953010	100,000	

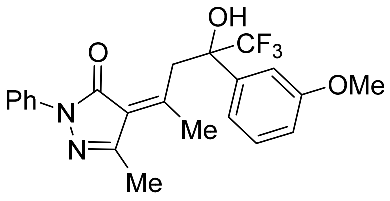

3af

Sample Name: AG-79 ADH 9010 1mL Vial Number: 1

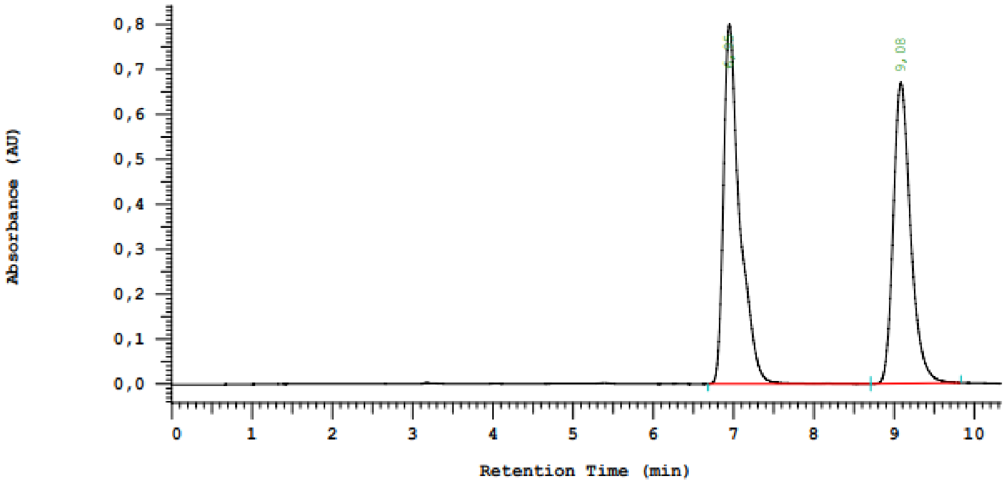

No.	RT	Area	Area %	Name
1	6,95	5656870	52,828	
2	9,08	5051130	47,172	
		10708000	100,000	

Sample Name: AG-119 ADH 9010 1mL Vial Number: 1

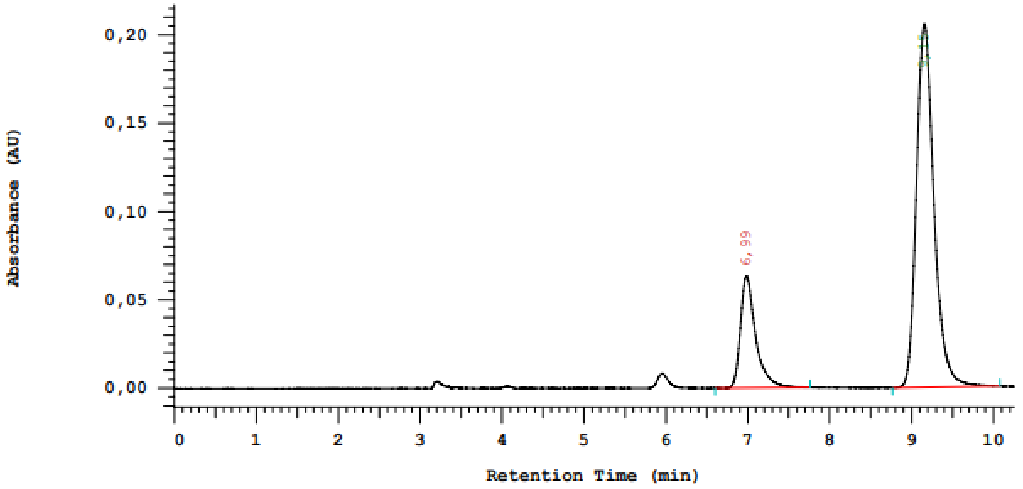

No.	RT	Area	Area %	Name
1	6,99	409300	21,093	
2	9,15	1531120	78,907	
		1940420	100,000	

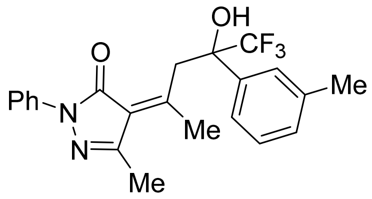

3ag

Sample Name: AG-85 ADH 9010 1mL   Vial Number: 1

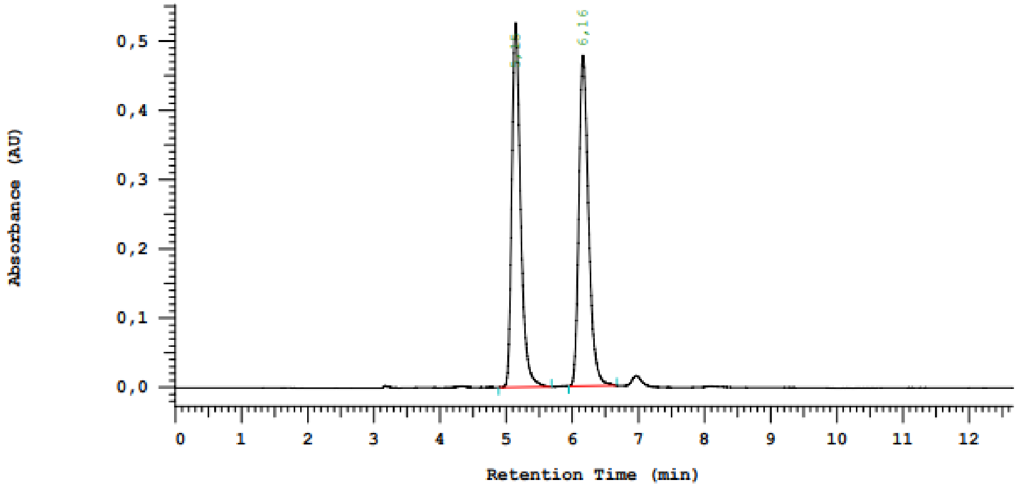

No.	RT	Area	Area %	Name
1	5,15	2445390	49,921	
2	6,16	2453085	50,079	
		4898475	100,000	

Sample Name: AG-120 ADH 9010 1mL   Vial Number: 1

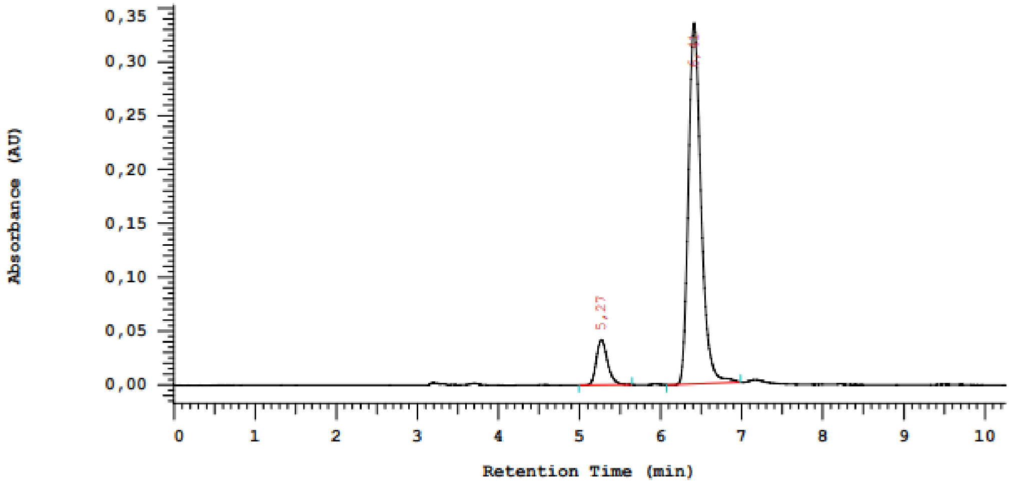

No.	RT	Area	Area %	Name
1	5,27	198980	9,944	
2	6,41	1801995	90,056	
		2000975	100,000	

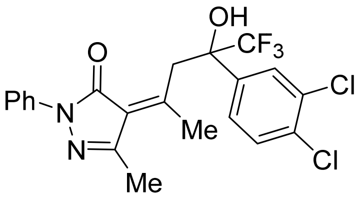

3ah

Sample Name: AG-112 ADH 9010 1mL Vial Number: 1

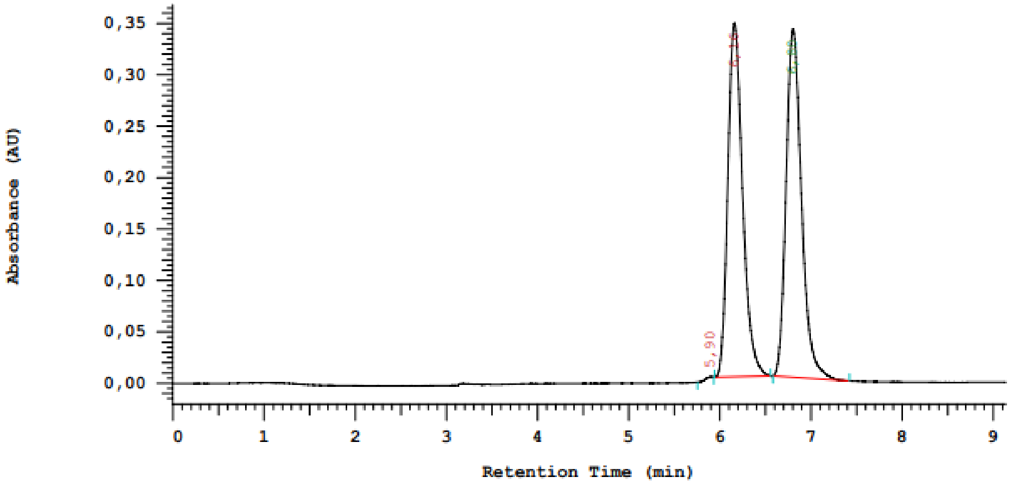

No.	RT	Area	Area %	Name
1	5,90	0	0,000	
2	6,16	1940125	49,317	
3	6,80	1993840	50,683	
		3933965	100,000	

Sample Name: AG-126 ADH 9010 1mL Vial Number: 1

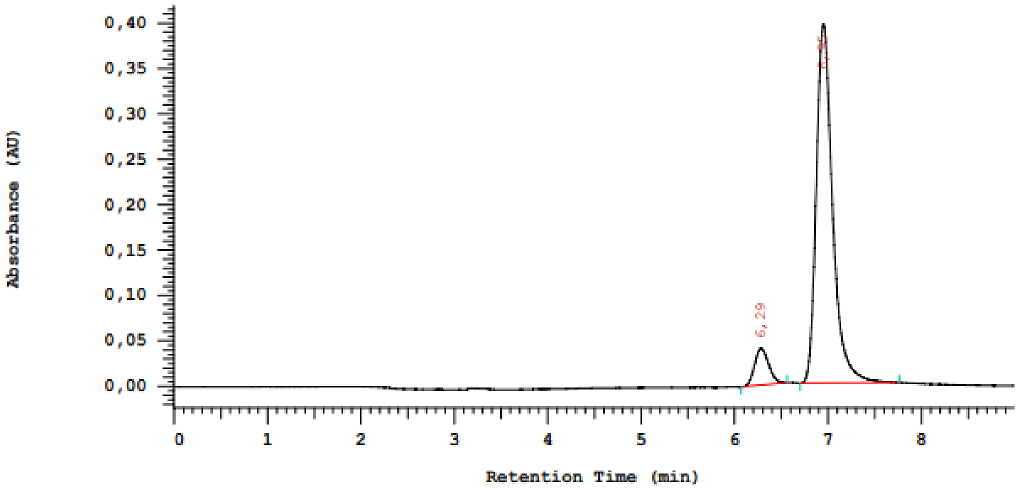

No.	RT	Area	Area %	Name
1	6,29	209800	8,031	
2	6,95	2402600	91,969	
		2612400	100,000	

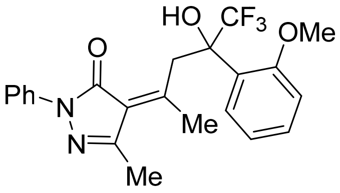

3ai

Sample Name: AG-86 ADH 9010 1mL Vial Number: 1

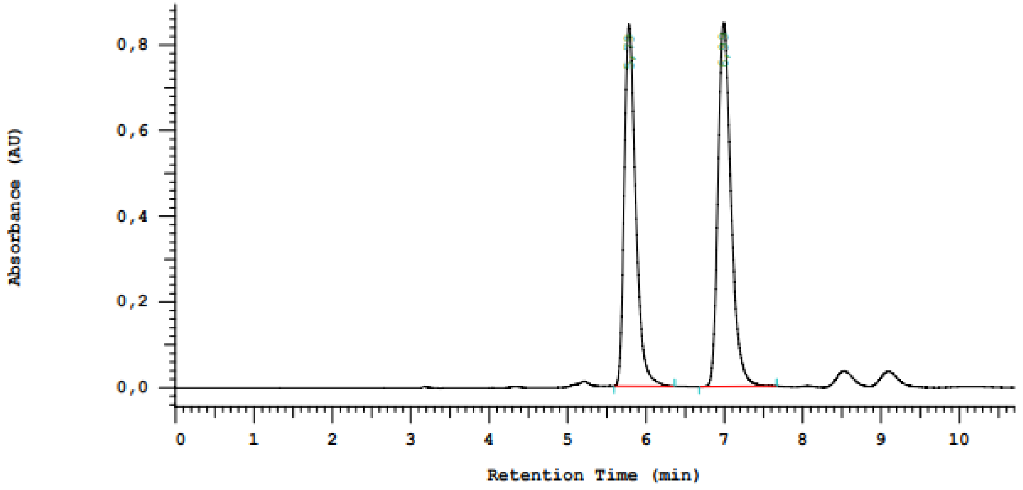

No.	RT	Area	Area %	Name
1	5,79	4255410	46,513	
2	6,99	4893390	53,487	
		9148800	100,000	

Sample Name: AG-128 ADH 9010 1mL Vial Number: 1

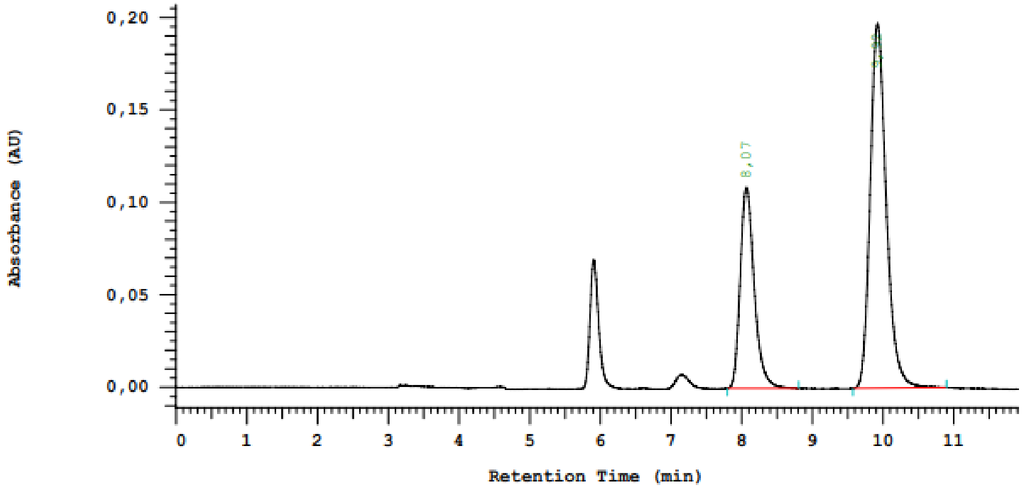

No.	RT	Area	Area %	Name
1	8,07	750160	32,396	
2	9,92	1565410	67,604	
		2315570	100,000	

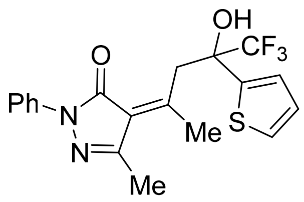

3aj

Sample Name: AG-83 ADH 9010 1mL Vial Number: 1

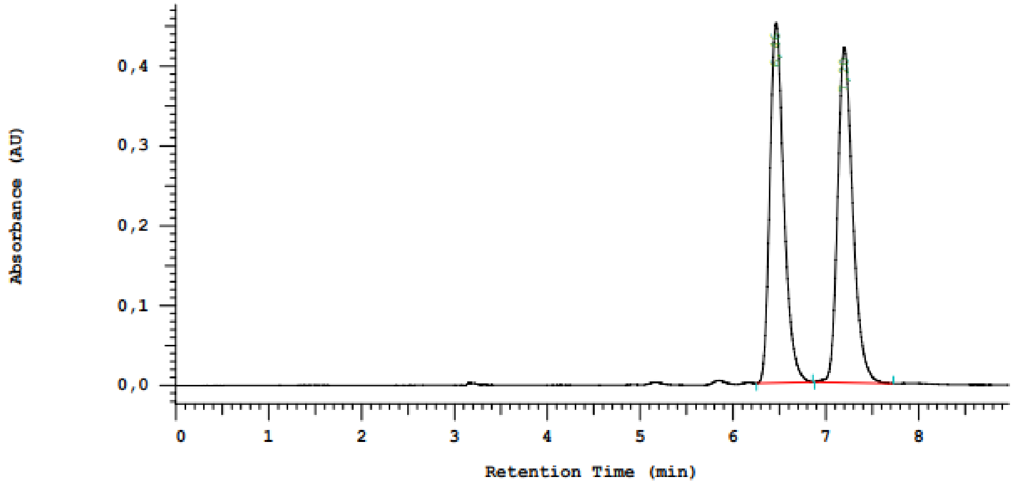

No.	RT	Area	Area %	Name
1	6,46	2362405	49,701	
2	7,20	2390820	50,299	
		4753225	100,000	

Sample Name: LC-849 ADH 9010 1mL Vial Number: 1

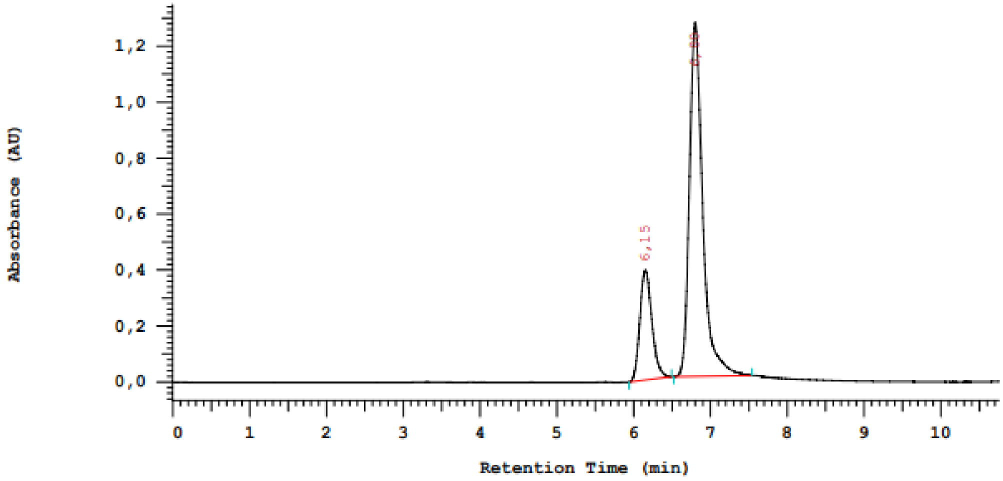

No.	RT	Area	Area %	Name
1	6,15	2140840	21,978	
2	6,80	7599864	78,022	
		9740704	100,000	

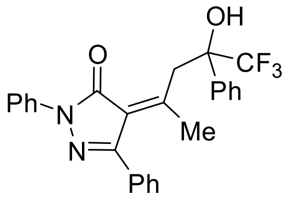

3ba

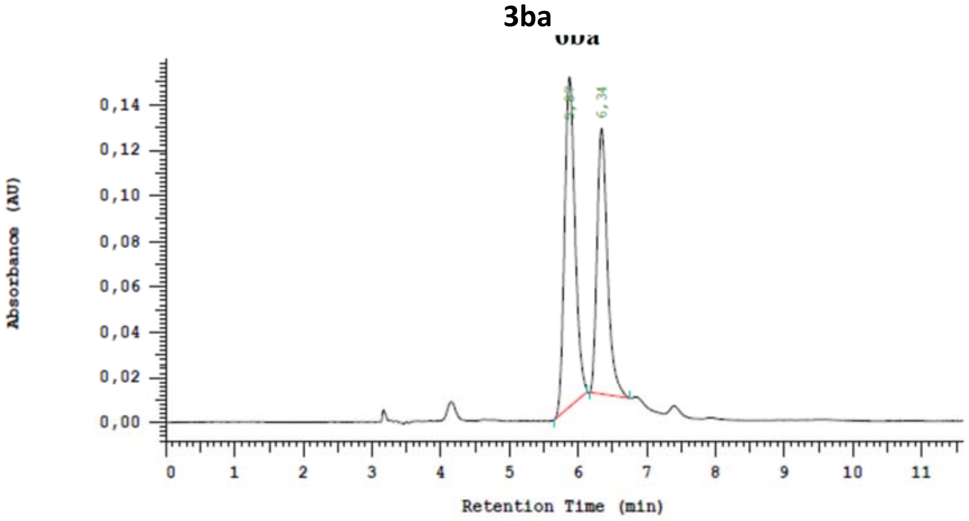

No.	RT	Area	Area %	Name
1	5,87	765840	55,284	
2	6,34	619450	44,716	
		1385290	100,000	

Sample Name: LC-855 ADH 9010 1mL   Vial Number: 1

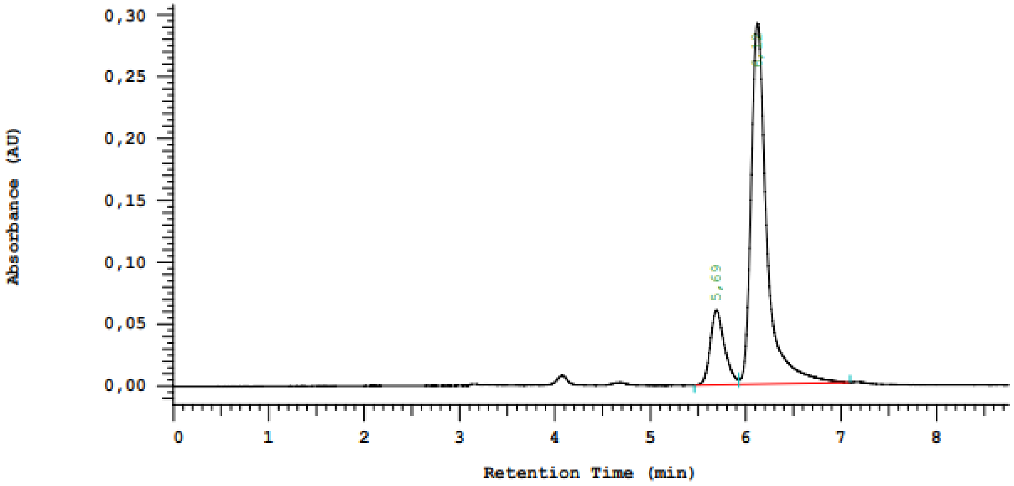

No.	RT	Area	Area %	Name
1	5,69	313940	15,938	
2	6,12	1655815	84,062	
		1969755	100,000	

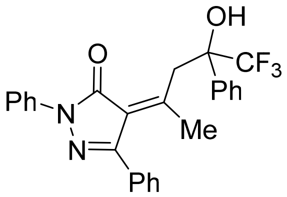

3ba (result at 0.2 M concentration)

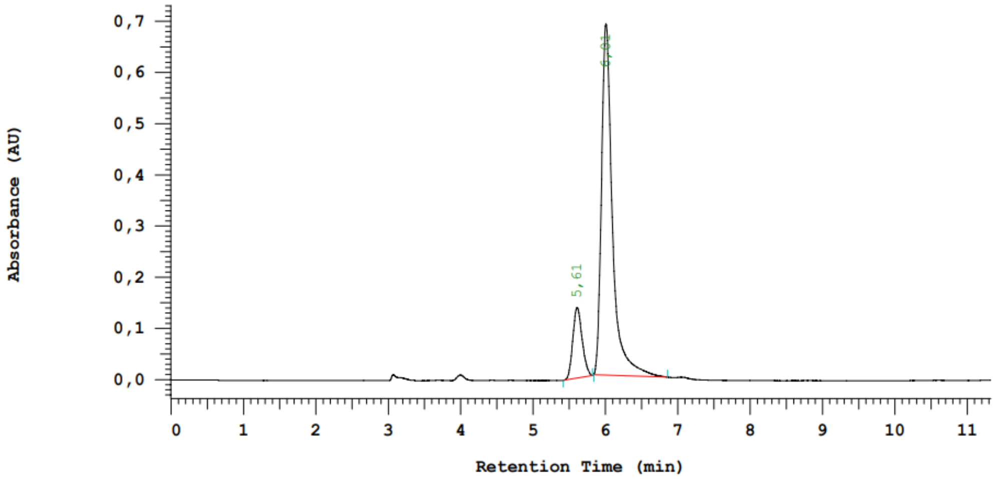

No.	RT	Area	Area %	Name
1	5,61	621400	14,730	
2	6,01	3597184	85,270	
		4218584	100,000	

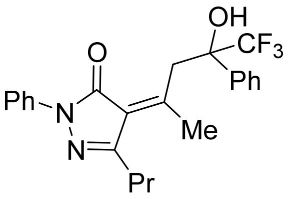

3ca

Sample Name: LC-832 ADH 9010 1mL   Vial Number: 1

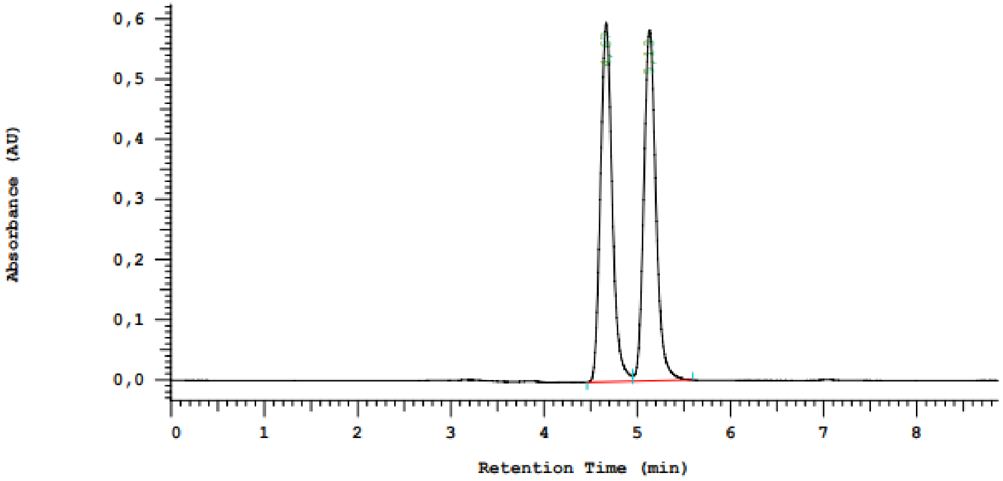

No.	RT	Area	Area %	Name
1	4,67	2628556	49,802	
2	5,13	2649493	50,198	
		5278049	100,000	

Sample Name: LC-844 ADH 9010 1mL   Vial Number: 1

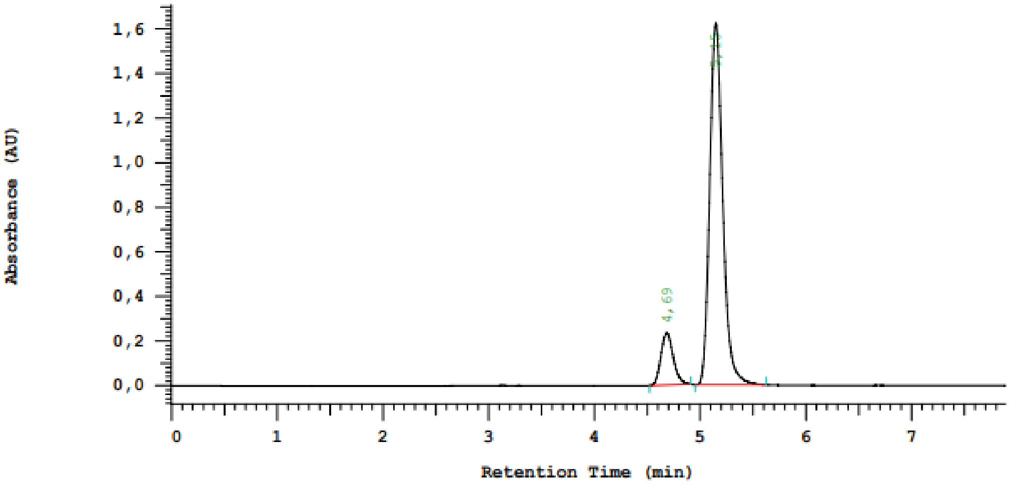

No.	RT	Area	Area %	Name
1	4,69	951045	12,094	
2	5,15	6913015	87,906	
		7864060	100,000	

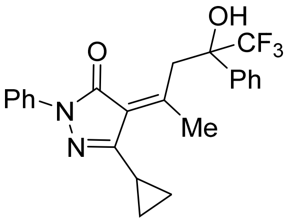

3da

Sample Name: LC-833 ADH 9010 1mL   Vial Number: 1

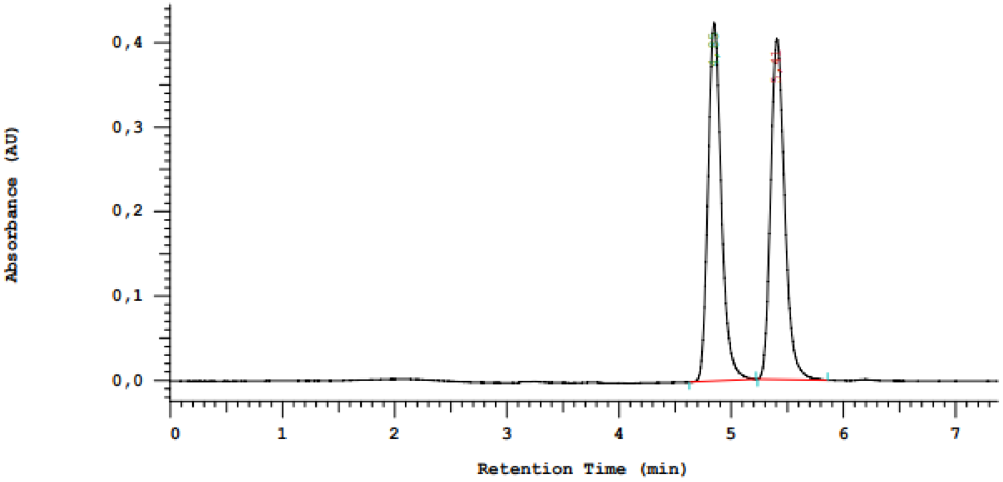

No.	RT	Area	Area %	Name
1	4,85	1764465	50,180	
2	5,41	1751830	49,820	
		3516295	100,000	

Sample Name: LC-845 ADH 9010 1mL   Vial Number: 1

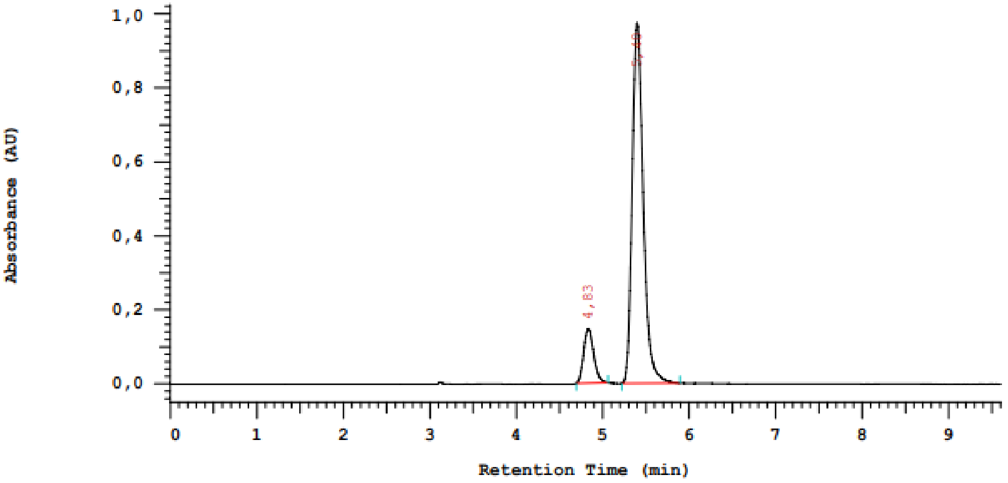

No.	RT	Area	Area %	Name
1	4,83	593995	12,028	
2	5,40	4344264	87,972	
		4938259	100,000	

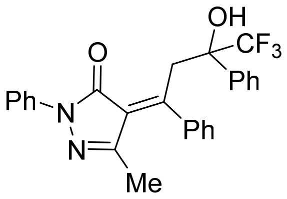

3ea

Sample Name: LC-854 IC 9010 1mL Vial Number: 1

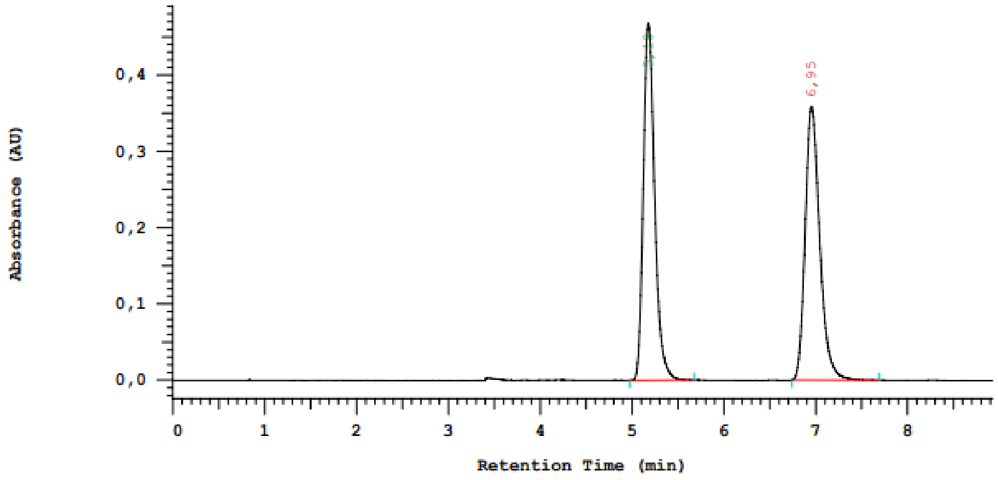

No.	RT	Area	Area %	Name
1	5,18	2005775	50,214	
2	6,95	1988655	49,786	
		3994430	100,000	

Sample Name: LC-854 IC 9010 1mL Vial Number: 1

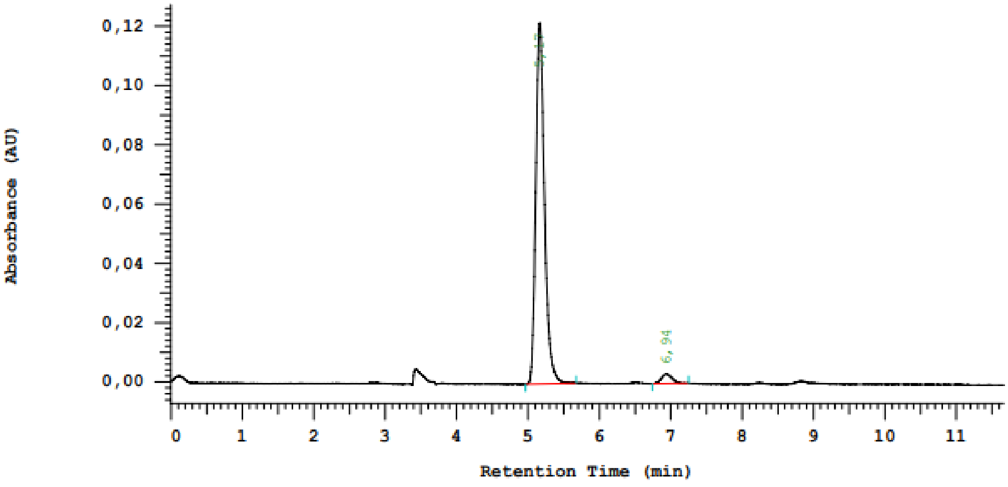

No.	RT	Area	Area %	Name
1	5,17	522190	96,966	
2	6,94	16340	3,034	
		538530	100,000	

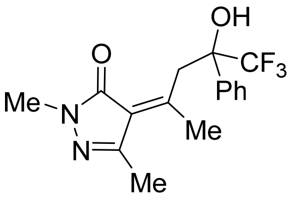

3fa

Sample Name: AG-127 ADH 9010 1mL Vial Number: 1

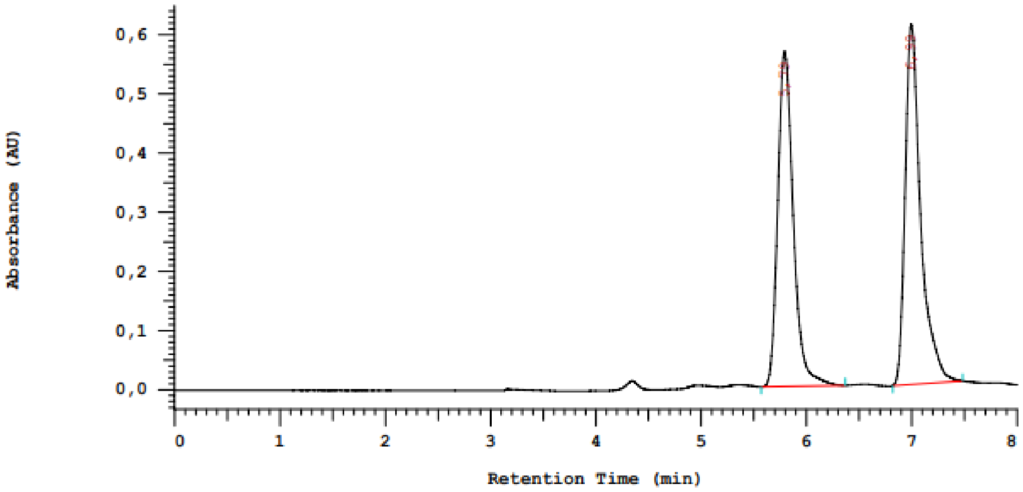

No.	RT	Area	Area %	Name
1	5,79	2919910	48,351	
2	6,99	3119030	51,649	
		6038940	100,000	

Sample Name: AG-124 ADH 9010 1mL Vial Number: 1

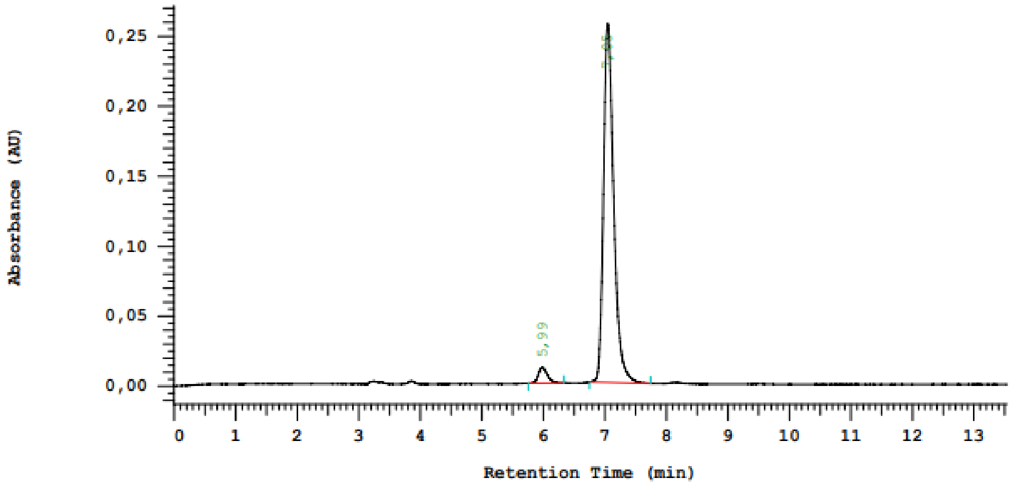

No.	RT	Area	Area %	Name
1	5,99	57410	3,735	
2	7,05	1479530	96,265	
		1536940	100,000	

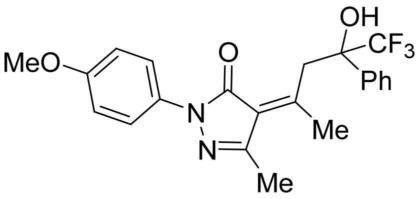

3ga

Sample Name: LC-835 ADH 9010 1mL Vial Number: 1

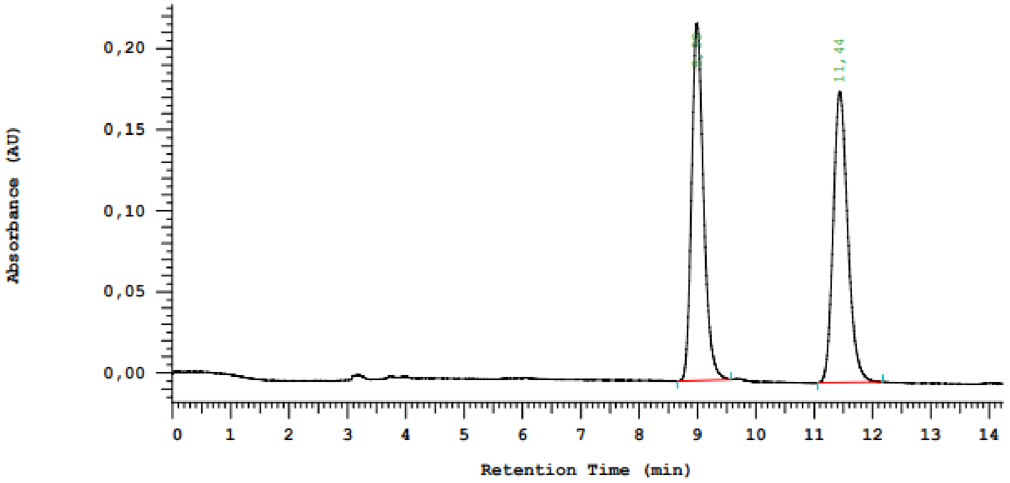

No.	RT	Area	Area %	Name
1	8,99	1562450	50,142	
2	11,44	1553610	49,858	
		3116060	100,000	

Sample Name: LC-853 ADH 9010 1mL Vial Number: 1

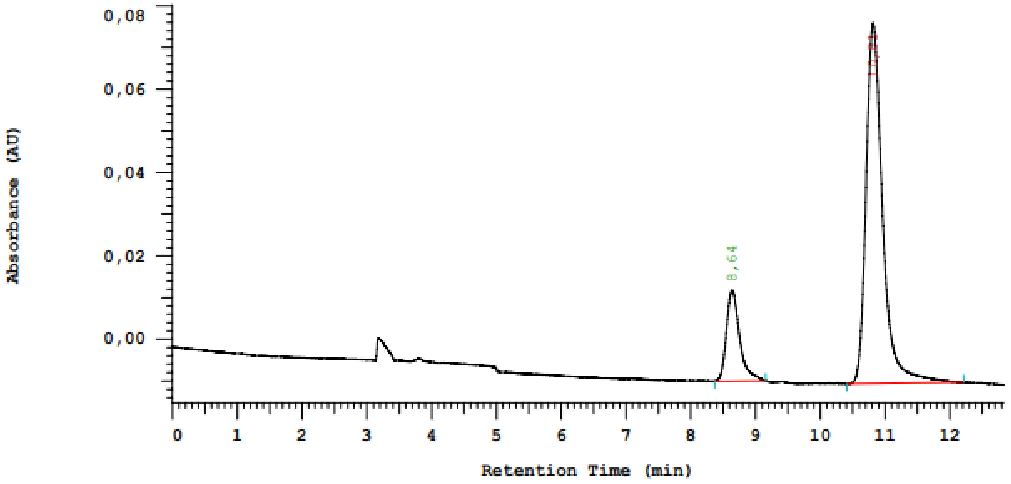

No.	RT	Area	Area %	Name
1	8,64	154470	16,874	
2	10,81	760980	83,126	
		915450	100,000	

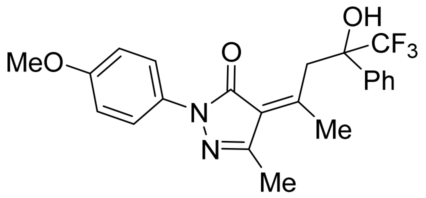

3ga (result at 0.2 M concentration)

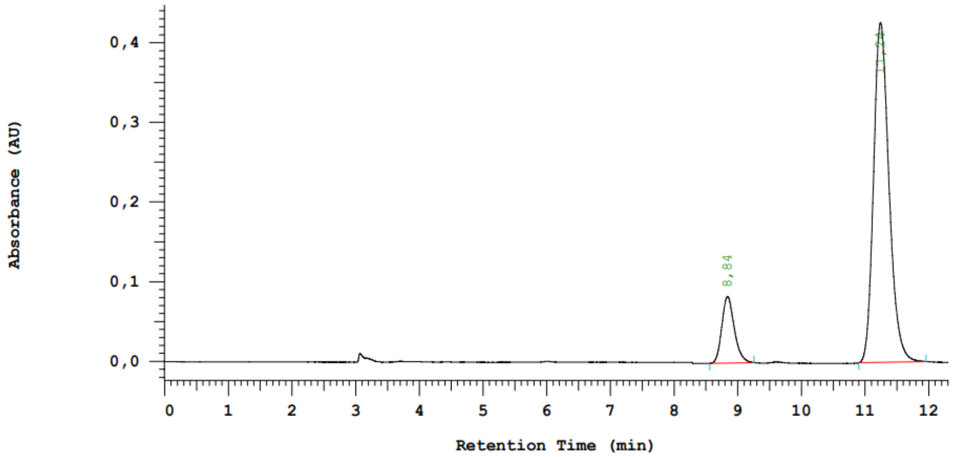

No.	RT	Area	Area %	Name
1	8,84	569990	13,733	
2	11,24	3580610	86,267	
		4150600	100,000	

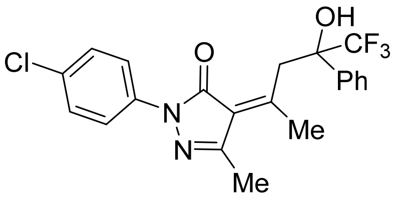

3ha

Sample Name: LC-843 ADH 9010 1mL   Vial Number: 1

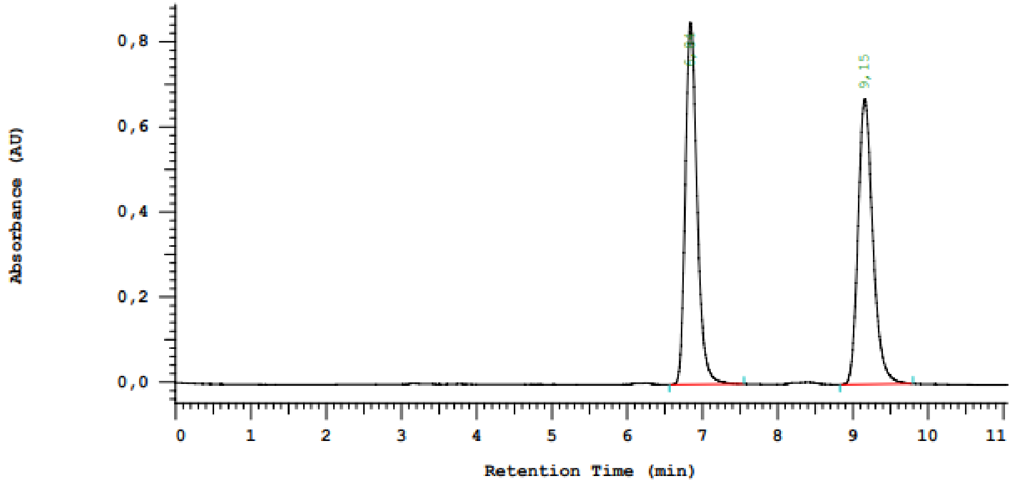

No.	RT	Area	Area %	Name
1	6,84	4593090	49,832	
2	9,15	4624095	50,168	
		9217185	100,000	

Sample Name: LC-852 ADH 9010 1mL   Vial Number: 1

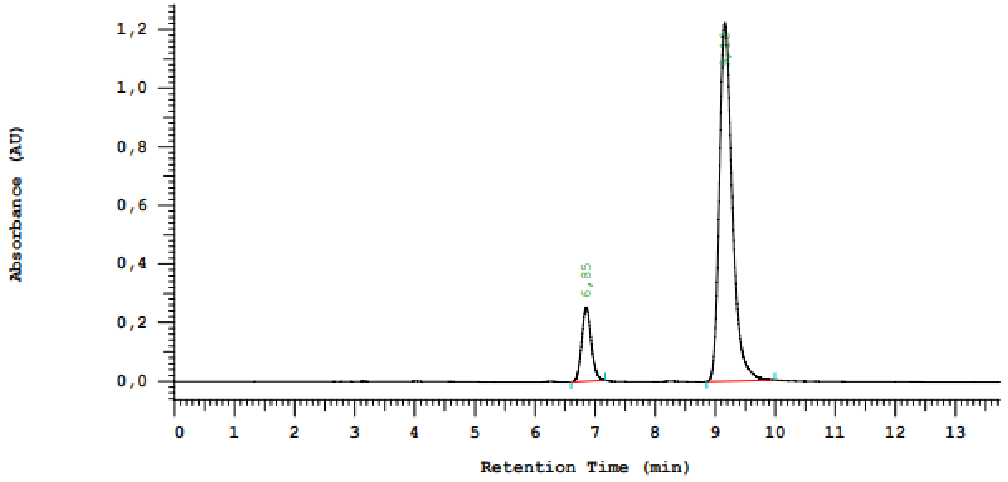

No.	RT	Area	Area %	Name
1	6,85	1391465	13,165	
2	9,16	9177670	86,835	
		10569135	100,000	

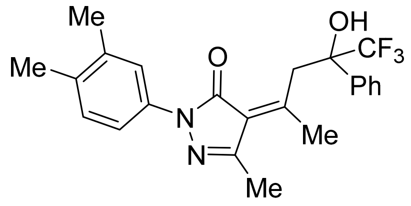

3ia

Sample Name: LC-837 ADH 9010 1mL   Vial Number: 1

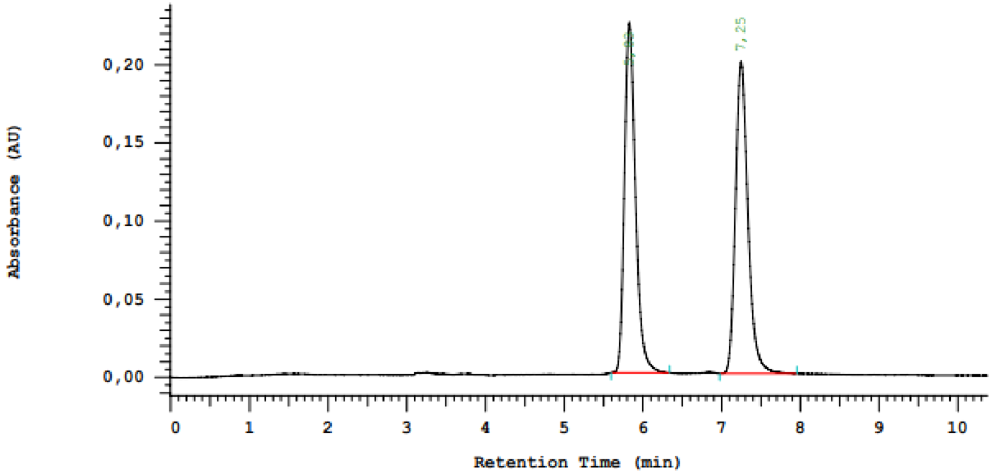

No.	RT	Area	Area %	Name
1	5,83	1095500	49,868	
2	7,25	1101290	50,132	
		2196790	100,000	

Sample Name: LC-851 ADH 9010 1mL   Vial Number: 1

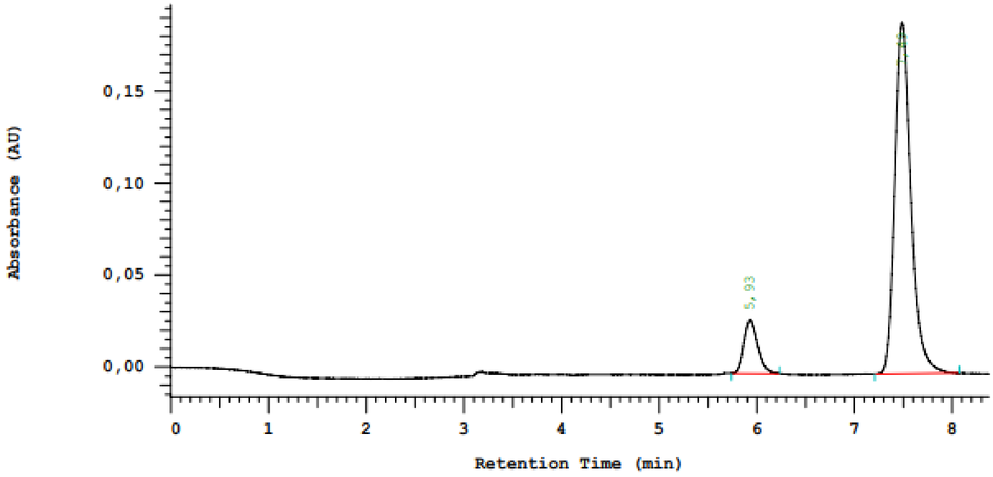

No.	RT	Area	Area %	Name
1	5,93	143120	11,520	
2	7,49	1099210	88,480	
		1242330	100,000	

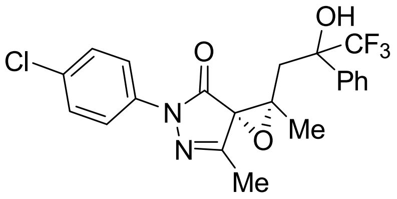

4 major

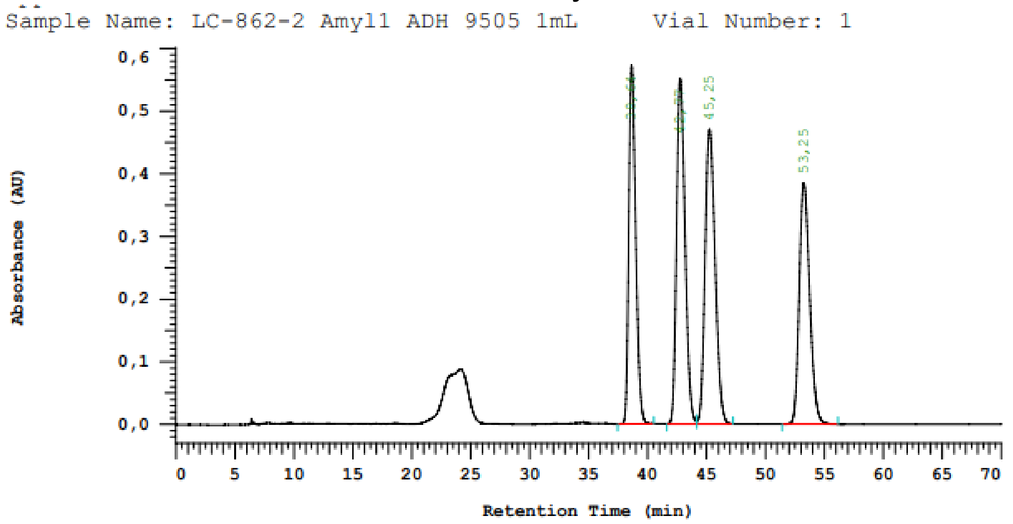

No.	RT	Area	Area %	Name
1	38,64	11722904	23,092	
2	42,77	13622099	26,833	
3	45,25	13660121	26,908	
4	53,25	11760884	23,167	
		50766008	100,000	

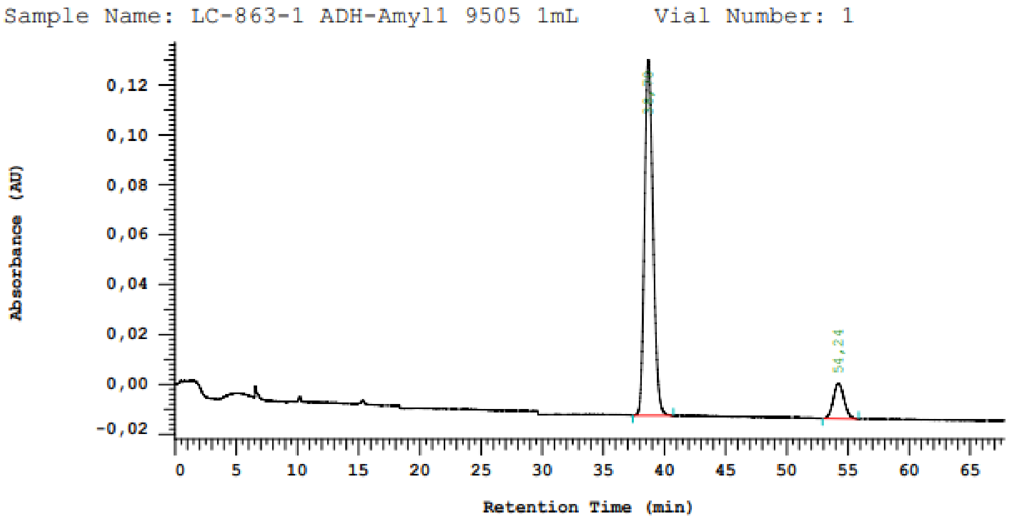

No.	RT	Area	Area %	Name
1	38,70	3374430	88,869	
2	54,24	422655	11,131	
		3797085	100,000	

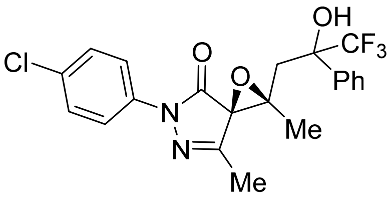

4 minor

Sample Name: LC-862-2 Amyl1 ADH 9505 1mL   Vial Number: 1

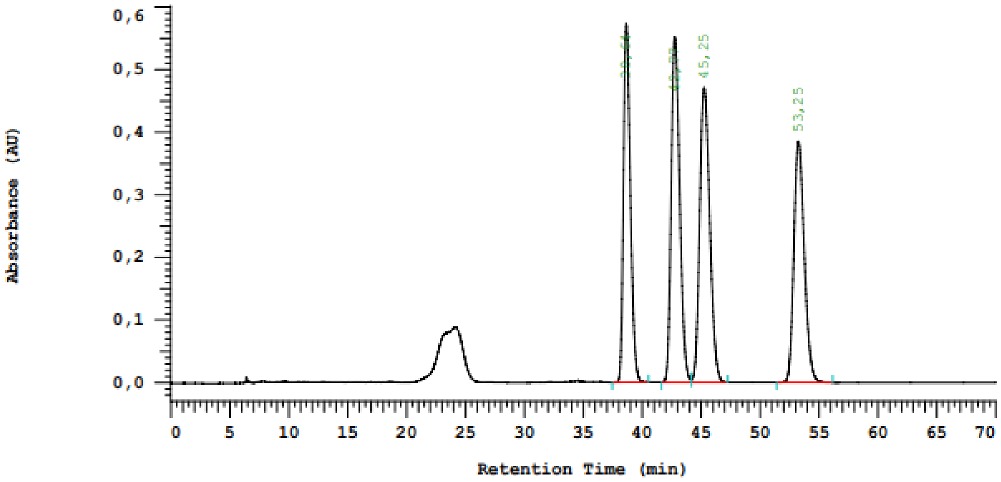

No.	RT	Area	Area %	Name
1	38,64	11722904	23,092	
2	42,77	13622099	26,833	
3	45,25	13660121	26,908	
4	53,25	11760884	23,167	
		50766008	100,000	

Sample Name: LC-863-2 ADH-Amylose1 9010 1mL Vial Number: 1

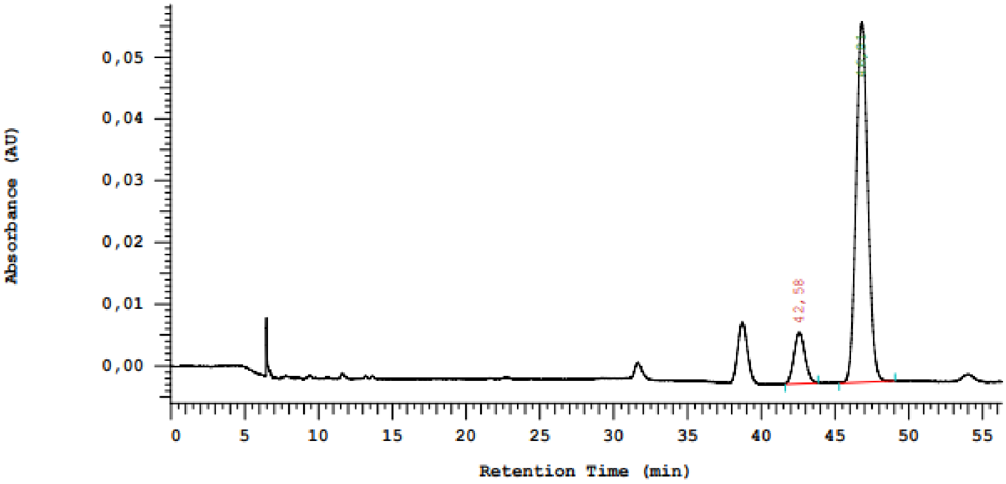

No.	RT	Area	Area %	Name
1	42,58	213720	11,800	
2	46,81	1597390	88,200	
		1811110	100,000	
